# Bioisostere-Driven Discovery of SePP: A Selenium-Containing Polypharmacological Agent Relevant to Fragile X Syndrome

**DOI:** 10.1021/acs.jmedchem.5c02700

**Published:** 2026-02-10

**Authors:** Jason Wallach, Sean Cameron, Michael Dybek, Branden Stanley, Christopher Orme, Nour Riad, Pierce Kavanagh, Simon D. Brandt, Adam Knapp, James Gamrat, Rebekah Jauhola-Straight, Adeboye Adejare, Alexander J. Rogier, Richa Tyagi, Clinton E. Canal

**Affiliations:** 1 Department of Pharmaceutical Sciences, Philadelphia College of Pharmacy. Saint Joseph’s University, 600 South 43rd St., Philadelphia, Pennsylvania 19104, United States; 2 Discipline of Pharmacology and Therapeutics, School of Medicine, Trinity Centre for Health Sciences, Saint James’s Hospital, Dublin, Ireland D08 W9RT; 3 The Alexander Shulgin Research Institute, 1483 Shulgin Road, Lafayette, California 94549, United States; 4 College of Pharmacy, Department of Pharmaceutical Sciences, 5225 Mercer University, 3001 Mercer University Drive, Atlanta, Georgia 30341, United States; 5 Department of Chemistry, Philadelphia College of Pharmacy. Saint Joseph’s University, 600 South 43rd St., Philadelphia, Pennsylvania 19104, United States

## Abstract

Diphenidine is a prototypical 1,2-diarylethylamine that functions as an uncompetitive *N*-methyl-d-aspartate receptor (NMDAR) antagonist and monoamine reuptake inhibitor. To examine the effects of phenyl-ring bioisosteric replacement within this scaffold, a series of diphenidine analogs incorporating chalcogen heterocycles (2-furan, 2-thiophene, 3-thiophene, and 2-selenophene) was synthesized. Compounds were evaluated for in vitro binding to rat forebrain NMDARs and inhibition of human DAT, NET, and SERT in cell-based assays, enabling assessment of polypharmacology. In silico analyses (molecular volume, tPSA, electrostatic surfaces, stockholder charges) and induced-fit docking were used to rationalize structure–activity relationships. The 2-selenophene analog SePP is notable given the underexplored role of selenium in medicinal chemistry. SePP exhibited favorable polypharmacology, good brain penetration in mouse pharmacokinetic studies, and prevented audiogenic seizures in *Fmr1* knockout mice (10 mg/kg, i.p.) without impairing motor coordination. These findings support further exploration of SePP for fragile X syndrome.

## Graphical abstract

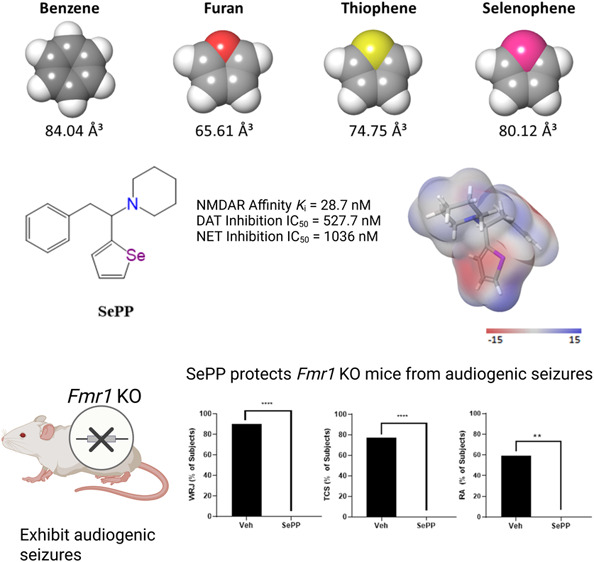

## Introduction

Bioisosterism is an important concept in medicinal chemistry and drug design.^1–3^ Bioisosterism refers to the substitution of atoms or functional groups with alternatives that maintain similar structural and physicochemical properties while modulating biological behavior. In practice, bioisosteric replacement is not intended to preserve biological effects wholesale, but rather to retain desired activity while attenuating liabilities such as off-target interactions, poor pharmacokinetics, or metabolic instability.^3,4^

Selenium-containing heterocycles, like selenophene, remain underrepresented in medicinal chemistry despite offering unique physicochemical and pharmacological properties relative to their sulfur and oxygen analogs.^5–8^ In relation to sulfur and oxygen atoms, selenium has a larger polarizable van der Waals (VDW) radius.^5^ As a result, selenophene, while overall similar in shape to O and S-heterocyclic analogs, furan and thiophene, respectively, possesses some distinct physicochemical properties^9^ including higher lipophilicity,^5^ altered surface electronics, and improved antioxidant activity.^5,10^ Selenium, and specifically selenophene, has been successfully incorporated in a number of preclinical drug discovery projects, including anticancer, antimicrobial, antidepressant, and anticonvulsant agents.^5,11–13^ Despite these encouraging findings, no organoselenium compound is an FDA-approved medication. This may soon change as ebselen is a selenium-containing molecule that has demonstrated promising pharmacological activity across numerous indications^14^ including stroke, bipolar disorder, tinnitus and sensorineural hearing loss.^15–17^ Additionally a benzoselenazole derivative, fluselenamyl (Figure 1), is being developed as a positron emission tomography (PET) imaging ligand for detecting beta-amyloid plaques in Alzheimer’s disease.^18–20^ These compounds underscore the promise of organoselenium compounds in medicinal chemistry. The development of novel drugs incorporating selenium would represent a significant advance in medicinal chemistry, as it expands the atomic palette available for molecular design, analogous to the transformative impact of fluorine and boron, which have enabled new physicochemical properties, bioisosteric strategies, and therapeutic modalities.^21–24^

**Figure 1:**
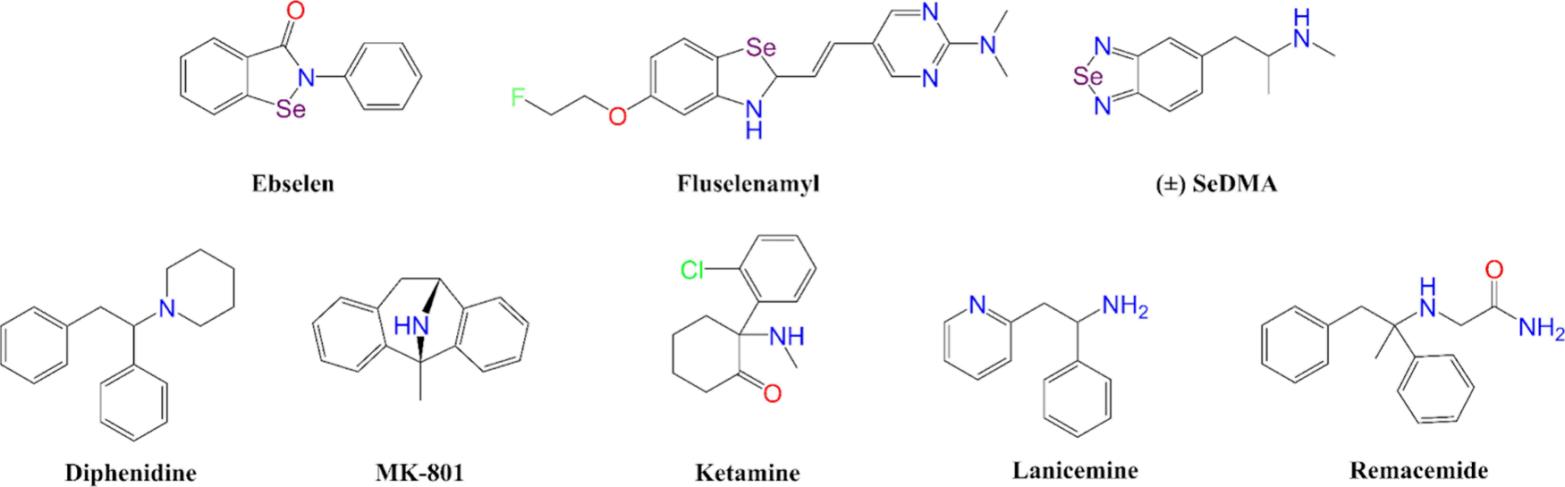
Investigational organoselenium containing compounds (top row), and representative NMDA receptor antagonists (bottom row).

Diphenidine appears to have been first synthesized in 1924.^25^ Diphenidine and many other 1,2-diarylethylamines (Figure 1), including lanicemine, and ephenidine, are uncompetitive *N*-methyl-d-aspartate receptor (NMDAR) antagonists that act by blocking the active state of NMDARs by binding to the phencyclidine (PCP) site located inside the receptor channel.^25^ NMDAR antagonists have shown broad therapeutic efficacy for several indications, including neurodegenerative disease, treatment resistant depression (TRD), major depressive disorder (MDD), epilepsy, suicidality, as well as in acute and chronic pain indications.^26–32^ A number of NMDAR antagonists are FDA-approved drugs, and a recent example, is the FDA approval of Spravato (esketamine) for TRD and MDD symptoms in suicidality, as well as Auvelity (dextromethorphan (DXM) and bupropion) approved for MDD. NMDAR antagonists are being extensively investigated clinically for their potential in other areas including tinnitus, post-traumatic stress disorder (PTSD), and alcohol use disorder.^33–36^ Two 1,2-diarylethylamine-based NMDAR antagonists (Figure 1) have advanced to Phase II clinical evaluation: lanicemine for major depressive disorder and remacemide for epilepsy, stroke, and multiple neurodegenerative disorders.^37–39^ However, despite some promise, neither progressed to phase III trials. Although clinical development of remacemide has largely abated, clinical interest in lanicemine continues, exemplified by a recent trial in patients with PTSD.^40–42^ Lanicemine remains a valuable research probe for human clinical pharmacology studies into NMDARs.^43–45^

Monoamine neurotransmitter reuptake transporters for dopamine (DAT), norepinephrine (NET), and serotonin (SERT) are members of the neurotransmitter sodium symporter family. These transporters share a conserved structural architecture, the LeuT fold, consisting of 12 transmembrane α-helices arranged in an inverted-repeat topology.^46^ The monoamine transporters regulate synaptic neurotransmitter levels and represent key pharmacological targets of psychoactive drugs, including psychostimulants, antidepressants, and analgesics.^46,47^ As we and others have previously reported, various 1,2-diarylethylamines, including diphenidine, bind to and inhibit DAT and NET with selectivity over SERT.^25^ Polypharmacology involving NMDAR and monoamine reuptake inhibition may have use in various psychiatric and neurological indications in which these targets are relevant including epilepsy, pain, neurodegenerative disease, and depression. For example, Auvelity, a treatment for MDD, is a combination product of DXM, an NMDAR antagonist and SERT inhibitor, and bupropion, a DAT and NET inhibitor and CYP2D6 inhibitor. Thus, 1,2-diarylethylamines represent a valuable scaffold that possesses favorable NMDAR antagonist and monoamine reuptake transporter inhibitor polypharmacology for next-generation therapeutics.^25^

To evaluate the impact of phenyl ring bioisostere substitution on 1,2-diarylethylamine receptor pharmacology, a series of phenyl-ring bioisostere analogs of diphenidine were designed (Figure 2) and synthesized using a two-step one-pot organozinc modified Mannich reaction (Scheme 1). The series was then pharmacologically characterized for binding affinities at the NMDAR PCP site as well as inhibition of monoamine reuptake activity at DAT, NET and SERT. In silico modeling and docking studies were then performed to provide insights into the observed structure–activity relationship (SAR).

**Figure 2:**
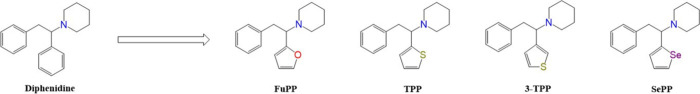
Design of 1,2-diarylethylamine phenyl ring bioisosteres series from diphenidine.

**Scheme 1:**
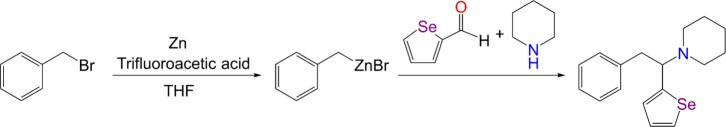
Representative Synthetic Scheme Showing the Two-Step One-Pot Organozinc-Modified Mannich Reaction Used to Synthesize SePP and Analogs

Finally, the selenophene analog, SePP, was selected for further in vivo testing in a mouse model of fragile X syndrome (FXS).^48^ FXS is a monogenic neurodevelopmental disorder caused by a mutation in the *FMR1* gene, leading to intellectual disability, neurobehavioral issues (including attention deficit hyperactivity disorder, anxiety, and sensory hypersensitivity), autistic symptoms, and seizures.^49^ *Fmr1* knockout (KO) mice serve as a construct-valid model of FXS, exhibiting audiogenic seizures (AGS) that reflect neuronal hyperexcitability inherent in FXS. The age-related prevalence of AGS in *Fmr1* KO mice (high in juveniles, low in adults) mirrors seizure patterns in FXS, where approximately 15% of individuals—especially children—experience seizures.^50^ In addition to modeling seizures, AGS may also model auditory hypersensitivity experienced by most individuals with FXS.^51–56^ NMDARs have been implicated in the pathophysiology of FXS.^57,58^ For example, inhibition of NR2A-containing NMDARs ameliorated synaptic plasticity dysfunction in an *Fmr1* KO mouse model, a dysfunction hypothesized to underlie sensory hypersensitivity in patients with FXS.^59^ In addition to demonstrating efficacy in various psychiatric and neurological conditions,^26,33–36^ uncompetitive NMDAR antagonists are known to have potent anticonvulsant effects.^60–65^ Given these considerations, the selenophene analog SePP was assessed for anticonvulsant activity in *Fmr1* KO mice. Because some NMDAR antagonists disrupt motor function, including coordination and balance, we also evaluated SePP’s effects on mouse performance in an elevated beam test. We hypothesized that the DAT/NET inhibition activity could counter the motor impairment typically seen with efficacious doses of NMDAR antagonists.

## Results and Discussion

The target compounds were synthesized using a two-step, one-pot organozinc-modified Mannich reaction (Scheme 1), which we have used previously to synthesize various 1,2-diarylethylamines.^66,67^ Briefly, benzyl zinc bromide is prepared from benzyl bromide and zinc dust (activated with trifluoroacetic acid) in tetrahydrofuran (THF). The aldehyde and amine were then added in quick succession to this solution and stirred overnight under an inert atmosphere. Yields ranged from 35.7–82.7%.

*N*-Methyl-d-aspartate receptors (NMDAR) are ligand and voltage-gated transmembrane cation channels. NMDARs are tetrameric receptors with 2-fold symmetry, composed of two NR1 subunits and two from either NR2 (A–D) or NR3 (A–B) subunits. The various combinations and stoichiometries of these subunits result in multiple distinct NMDAR subtypes.^68^ The physiological and pathological relevance of distinct NMDAR subtypes remains an active area of investigation.^69,70^ NMDARs have a number of modulatory sites for drug action. Compounds that bind to the PCP site, such as MK-801, ketamine, PCP, DXM, and diphenidine (Figure 1), act as use-dependent, noncompetitive NMDAR channel blockers, and are often referred to as uncompetitive antagonists or channel blockers.^25,71^ Recent X-ray diffraction and cryo-electron microscopy (cryo-EM) structures have confirmed that these uncompetitive antagonists bind to the PCP site within the NMDAR channel, physically occluding ion conduction.^72–75^ Within the PCP binding site of the NMDAR channel, residues are highly conserved across NR2 subunits.^74,76^ Notably, topologically equivalent residues in both NR1 and NR2 subunits are highly conserved, contributing to the formation of a 4-fold symmetric channel pore and binding site.^74^ Consistent with the high degree of NR2 subunit symmetry, uncompetitive NMDAR antagonists exhibit largely comparable in vitro potencies across investigated NMDAR subtypes^77^ except in the presence of Mg^2^^+^, which induces functional differences—most notably, an approximately 10-fold higher potency preference for NR2C-containing subtypes.^77–79^ PCP-site NMDAR binding studies performed in rat forebrain have been highly predictive of in vivo rank-order potencies in humans and rodents for various uncompetitive NMDAR antagonists.^25,61,71,80,81^ Rat forebrain NMDAR binding affinities (p*K*_i_ and *K*_i_) for the bioisostere series and MK-801, determined by [^3^H]-MK-801 competitive radioligand binding assays, are summarized in Table 1 with mean concentration–response curves (N = 3) shown in Figure 3. All but one compound in the bioisostere series exhibited potent binding affinities at rat forebrain NMDARs, i.e., in the low nanomolar (10^–8^ M) range. In fact, affinities for SePP (*K*_i_ = 28.7 nM), TPP (*K*_i_ = 25.4 nM), 3-TPP (*K*_i_ = 41.1 nM), and diphenidine (*K*_i_ = 27.7 nM) were nearly identical. The one exception to this trend was the furan analog FuPP (*K*_i_ = 128.8 nM), which exhibited an approximately 4-fold reduction in NMDAR affinity compared to the other compounds.

**Figure 3:**
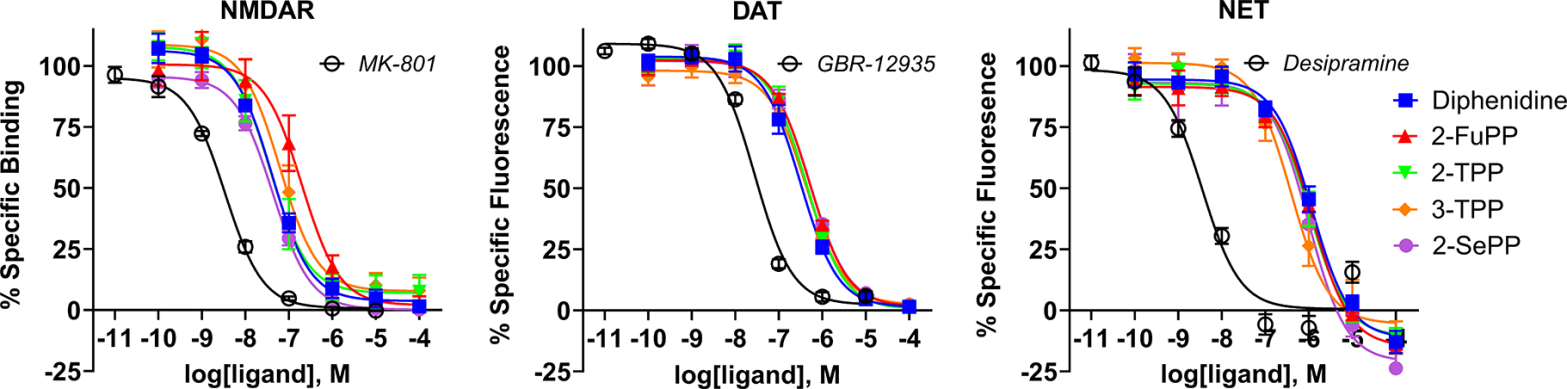
NMDAR binding and DAT and NET reuptake inhibition graphs for the 1,2-diarylethylamine bioisostere series and controls. Mean ± SEM (*N* = 3).

**Table 1:** NMDAR Binding Affinities (*K*_i_ and p*K*_i_) and Monoamine Reuptake Inhibition Potencies (IC_50_ and pIC_50_) at DAT, NET, and SERT^a^

	pharmacological targets							
	NMDAR		DAT		NET		SERT	
compound	*K*_i_ (nM)	p*K*_i_	IC_50_ (nM)	pIC_50_	IC_50_ (nM)	pIC_50_	IC_50_ (nM)	pIC_50_
MK-801	2.3 ± 0.5	8.657 ± 0.083						
GBR-12935			28.1 ± 3.03	7.557 ± 0.047				
desipramine					3.55 ± 0.1	8.451 ± 0.015		
citalopram							83.3 ± 8.3	7.095 ± 0.057
diphenidine	27.7 ± 5.4	7.576 ± 0.089	311.1 ± 48.9	6.517 ± 0.067	1206.1 ± 193.1	5.930 ± 0.072	>10,000	<5.00
FuPP	128.8 ± 26.5	6.910 ± 0.095	513.9 ± 23.7	6.290 ± 0.020	1193.0 ± 119.3	5.928 ± 0.043	>10,000	<5.00
TPP	25.4 ± 6.9	7.625 ± 0.110	428.8 ± 59.8	6.377 ± 0.063	1061.5 ± 243.5	6.000 ± 0.108	>10,000	<5.00
3-TPP	41.1 ± 12.6	7.424 ± 0.127	442.1 ± 61.6	6.362 ± 0.057	419.4 ± 117.6	6.408 ± 0.112	>10,000	<5.00
SePP	28.7 ± 6.3	7.561 ± 0.090	527.7 ± 50.5	6.281 ± 0.040	1036.0 ± 172.0	5.999 ± 0.081	>10,000	<5.00

a Mean ± SEM. N = 3, for p*K*_i_/pIC_50_ < 5.00, N = 2.

To provide insights into the binding mode of the 1,2-diarylethylamine series at the NMDAR, in silico docking studies were performed. One complexity of NMDAR docking studies that we have encountered is the fact that the PCP-site and related regions within the NR1/NR2 NMDAR channel pore, is highly symmetric, such that several rings of identical residues (one from each of the 4 subunits of the tetramer) are present, creating a pore formed by a series of conserved concentric intrapore residues including the threonine (Thr) ring, a leucine/valine (hydrophobic) ring, and asparagine (Asn) ring.^72,74^ Furthermore, ligand–receptor interactions are relatively weak, being devoid of any strongly anchoring ion−ion Coulombic interactions.^72,74^ These factors conspire to create an environment in which multiple distinct low-energy binding modes likely coexist in equilibrium, as evidenced by a cryo-EM study that found distinct binding poses for esketamine within the PCP site of a GluN1a-GluN2B NMDAR.^74^ For this reason, a docking constraint was implemented such that the ligand cationic amine makes an NH^+^ · · · hydrogen bond interaction with the carbonyl oxygen of asparagine N615 on one of the GluN2B subunits of the receptor (PDB: 7SAC). Interaction between the cationic amine and N615 is a highly conserved interaction supported by mutagenesis studies, molecular dynamics, X-ray diffraction (XRD) and cryo-EM structures with numerous distinct NMDAR channel blockers including esketamine, arketamine, memantine, PCP, and (+)-MK-801.^72,74,75,82^ This constraint returned poses with (+)-MK-801 and esketamine, that were consistent with experimental cryo-EM data, in contrast to runs without the constraint. For example, esketamine, the experimental ligand of the 7SAC structure, gave reasonable poses in which key ligand-side chain interactions were present. However, the best root-mean-square deviation of atomic positions (RMSD) was lower than ideal: 3.056 Å (Figure S2A, Supporting Information). The induced-fit docking (IFD) poses obtained for the conformationally constrained 1,2-diarylethylamine (+)-MK-801 were better relative to its experimental XRD pose in PDB: 5UOW (RMSD = 1.870 Å, Figure S2B, Supporting Information). Figure 4A and supplemental Figure S2A–O illustrate that the compounds of the bioisostere series can occupy a similar binding mode within the PCP-site of the NMDAR (PDB: 7SAC) relative to one another. Likewise, the binding interactions seen with the IFD of the 1,2-diarylethylamines show similarities to experimental poses of other NMDAR channel blockers including ketamine, PCP, and (+)-MK-801 (Figure S2C,D, Supporting Information) with respect to the orientation of the cationic amine and benzylamine aryl ring.

**Figure 4:**
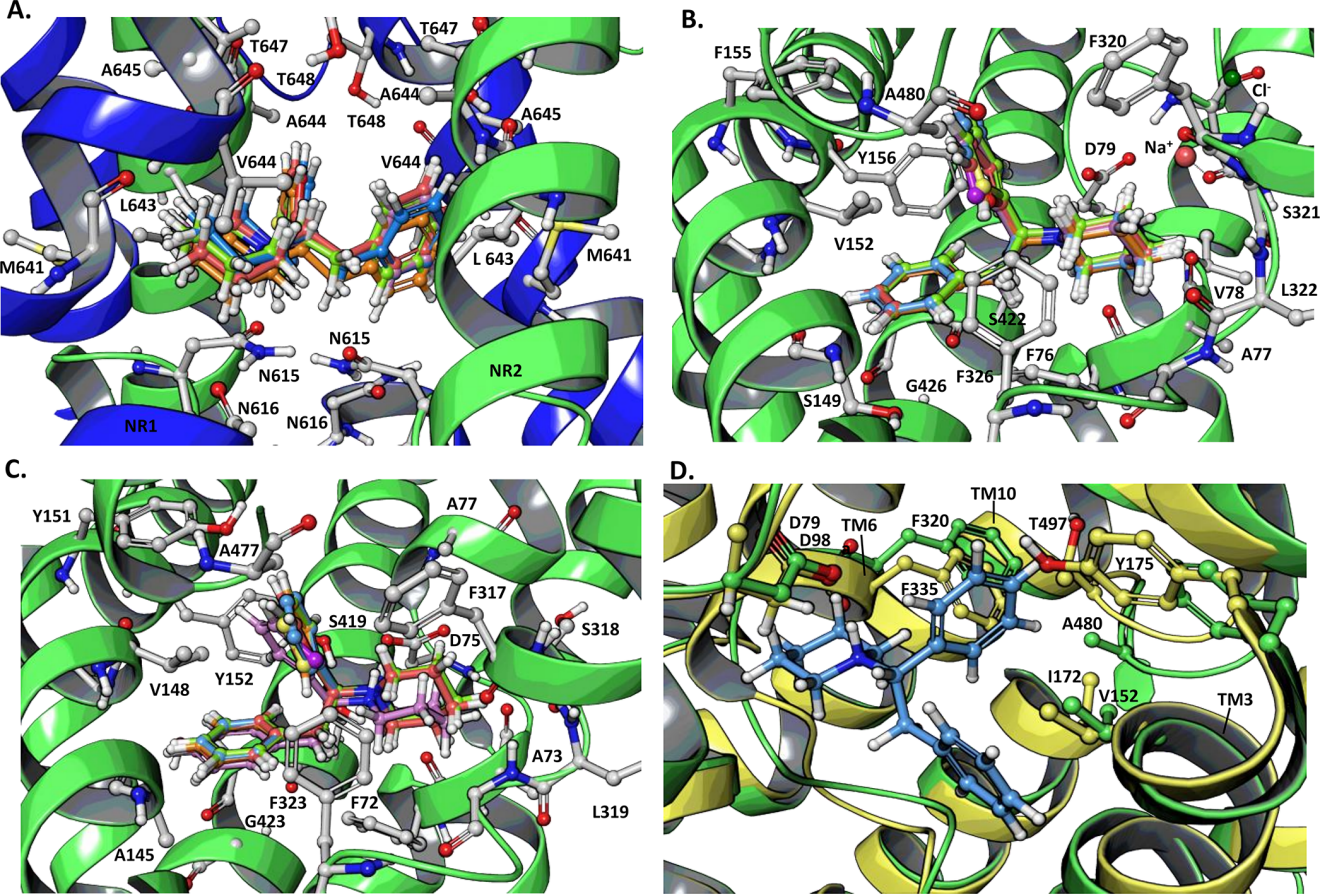
Induced fit docking overlays of 1,2-diarylethylamine bioisostere series. The *S*-enantiomers are shown here. Key binding site residues are displayed and labeled for each receptor. The side chain positions and protein backbone displayed are from the diphenidine IFD pose; side chains were similar across all structures. (A) NR1/NR2B NMDAR (PDB: 7SAC). (B) DAT (PDB: 8Y2G). (C) NET (PDB: 8Z1L). Color scheme: diphenidine (blue), FuPP (red), TPP (green), 3-TPP (orange), and SePP (purple). Results show the high degree of overlap in predicted binding poses of the series at NMDAR, DAT, and NET. (D) Structural comparison of (*S*)-diphenidine bound to the S1 site in DAT (green, PDB: 8Y2G) and SERT (yellow, PDB: 7LIA). Key binding site residues are displayed and labeled.

Overall, the IFD results support a similar binding mode among the compounds within the bioisostere series (Figure 4) suggesting the aryl bioisosteres can substitute well for one another. While molecular docking can inform SAR, it does not reliably predict receptor binding affinities.^83^ The IFD results fail to provide a clear explanation for the reduced affinity of FuPP compared to the other members of the series. Compared with the phenyl (diphenidine), thiophene (TPP and 3-TPP) and selenophene (SePP) analogs, the 4-fold reduced NMDAR affinity for FuPP is intriguing. To gain insight into the SAR, in silico predictors, including molecular volume, topological polar surface area (tPSA), electrostatic surface potential, and stockholder surface charge, were evaluated for the series (Figure 5 and Tables S3–S6, Supporting Information). Furan is unique in that it is the smallest ring system in the series, with a calculated molecular volume of 65.61 Å (Figure 5A) and has the highest surface charge density around the electronegative oxygen atom as illustrated by the stockholder charge (Figure 5B) and electrostatic surface potential (Figure 5C). One hypothesis is that the observed NMDAR binding affinity trend among the bioisostere series, where FuPP has the lowest NMDAR binding affinity, results from the smaller aryl ring volume (VDW radius) of furan relative to benzene, thiophene, and selenophene rings (Figure 5, and Table S3, Supporting Information). Aside from one cation-dipole interaction, between the cationic amine on the ligand and Asn ring residues of the NMDAR, all other intermolecular interactions between the ligands and receptor residues of the PCP binding site are VDW interactions (i.e., Keesom, Debye, and London dispersion) between uncharged (i.e., lacking a formal charge) portions of the ligand and uncharged polar and nonpolar residues of NMDARs. The smaller furan ring of FuPP could reduce these complementary VDW interactions, reducing the binding affinity. The fact that furan is also electronically distinct from the other rings is also notable and must be considered. tPSA (Table S3, Supporting Information), H, C and heteroatom stockholder charge calculations and electrostatic surface potentials (Figure 5B,C and Tables S4–S6, Supporting Information) clearly illustrate that furan is significantly more polar than the other aryl rings, with a higher electron charge density and a negative electrostatic potential energy, consistent with the higher electronegativity of the oxygen atom (Table S8, Supporting Information). In addition to impacting the strength of VDW interactions within the PCP binding site, the higher partial charge density on the furan ring and the strong H-bond acceptor nature of the oxygen could lead to an increased thermodynamic desolvation penalty for FuPP relative to the other compounds, that is responsible for a reduction in its binding affinity.^84,85^ These hypotheses could be probed by measuring differences in *K*_on_ and *K*_off_ NMDAR binding kinetics, coupled with molecular dynamics simulations, among the series.

**Figure 5:**
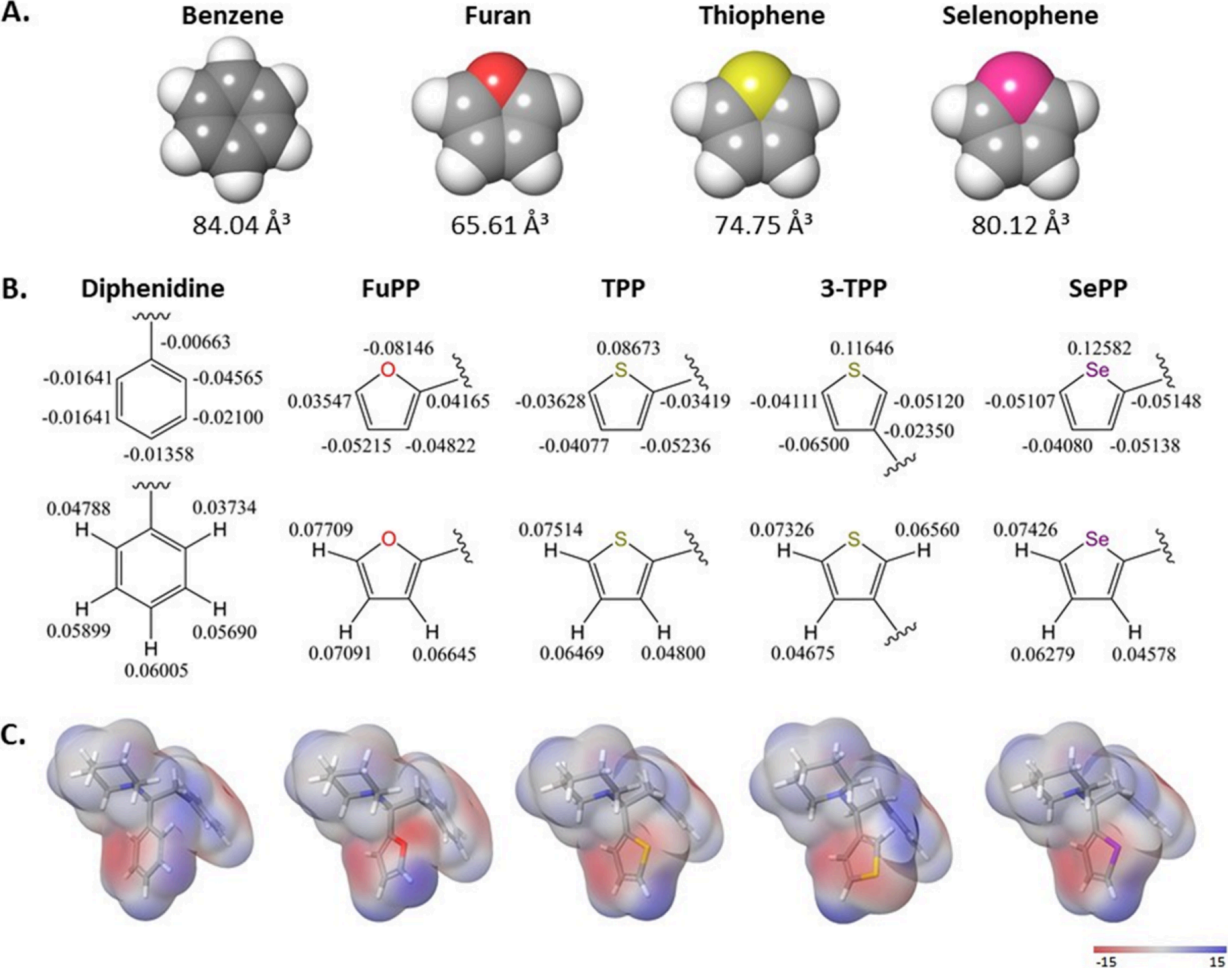
1,2-Diarlyethylamine bioisostere series in silico predictors. (A) Representative ring volumes for phenyl and 5-membered heteroaromatic bioisosteres. (B) Stockholder partial charges for each aryl ring portion of the full 1,2-diarylethylamine bioisostere. (C) Electrostatic surface potentials for 1,2-diarylethylamine bioisostere series. Note the smaller volume and higher negative charge on the furan oxygen in furan and FuPP which may explain its reduced NMDAR affinity.

We and others have previously reported that diphenidine and related 1,2-diarylethylamines (e.g., 2-MXP, ephenidine, and fluorolintane) act as DAT and NET inhibitors.^67^ Thus, the 1,2-diarylethylamine scaffold is useful to explore the impact of the ring bioisostere substitution on polypharmacology by exploring the effect across multiple pharmacological targets. To explore this, the impact of bioisosteric substitution was tested on reuptake inhibition activity at human DAT, NET, and SERT using an APP^+^ fluorescence uptake assay in stably transfected recombinant cells. With respect to inhibition of monoamine reuptake transporters, the series was found to inhibit DAT and NET at physiologically relevant concentrations (500–1000 nM range) but not SERT (>10,000 nM) (Table 1, Figure S7, and Table S9, Supporting Information). This selectivity profile is consistent with previous receptor binding and functional uptake studies for diphenidine and other 1,2-diarylethylamines.^67,86^ Relative to each other, the compounds exhibited similar potencies (IC_50_ estimates), within a half-order of magnitude, for both DAT and NET reuptake inhibition, showing ∼2-fold higher potencies for DAT over NET. The one notable exception was 3-TPP, which had approximately equipotent inhibition potencies for DAT and NET. To provide insights into possible binding modes of the compounds at DAT and NET, induced fit docking (IFD) studies were performed using the outward facing state of DAT and NET transporters (PDB: 8Y2G DAT, 8Z1L NET). The outward-facing conformation exposes the central binding site (S1) to the extracellular space and is the state bound by many known DAT inhibitors, including cocaine, GBR12909 and benzotropine^87^ and NET inhibitors including amitriptyline, nisoxetine, atomoxetine, nomifensine.^88^ As shown in Figure 4B,C (Figures S3A–M and S4A–M, Supporting Information), the IFD results suggest the compounds of the bioisostere series were able to adopt similar binding modes to one another within the S1 binding-site of the DAT and NET and make favorable intermolecular interactions with transporter S1 binding pocket amino acid residues implicated in reuptake inhibitor binding. The residues within the S1 binding sites of the DAT and NET are highly conserved, although some differences exist, with a notable difference being the substitution of a phenylalanine on TM3 (F155^TM3^) in the DAT for a tyrosine (Y151^TM3^) in NET.^87^ Many of the interactions observed are consistent with those seen in cryo-EM structures of known inhibitors that stabilize the transporter in the outward-facing state (e.g., methylphenidate, atomoxetine).^87^ An overlay of (*S*)-diphenidine with methylphenidate in DAT (PDB: 8Y2G) is present in Figure S3B, SI, and a corresponding overlay with atomoxetine in NET (PDB: 8ZIl) are presented in Figure S4B, Supporting Information. In the DAT IFD poses (Figure 4B), interactions include an ionic “salt bridge” between the protonated ammonium groups of the ligand and D79^TM1^, and aryl–aryl with F326^TM6b^, and Y156^TM3^, and F326^TM6b^ makes offset edge-to-face aryl–aryl interactions with the CH of both aryl rings. The aryl ring bioisosteres make an aryl–aryl offset interaction with phenylalanine DAT F335^TM6a^ and an edge-to-face type contact with the conserved TM3 tyrosine residue Y156^TM3^ with a bioisostere aryl ring CH projecting toward the tyrosine residue oxygen potentially forming an inappropriately termed “atypical” CH–O hydrogen bond.^89,90^ Comparable interactions are observed in the case of NET (Figure 4C) including D75^TM1^, F317^TM6a^, F323^TM6^, and Y152^TM3^, consistent with the high degree of conservation in the S1 binding sites between the DAT and NET.

SERT, like DAT and NET, is a member of the neurotransmitter sodium symporter solute carrier 6 (SLC6) family.^46^ The reason underlying the observed selectivity of the 1,2-diarylethylamines for DAT and NET over SERT is unknown.^91^ Many other 1,2-diarylethylamines lack physiological relevant binding affinity (>10,000 nM) at SERT or show very weak micromolar affinity binding affinities at SERT, despite higher submicromolar affinities for DAT and NET.^91^ Understanding the molecular basis for this selectivity could provide insight for designing selective reuptake inhibitors as well as insights for rationale polypharmacological drug design. As with DAT and NET, the S1 binding site in SERT contains an overall comparable architecture with many conserved residues; however, its amino acid composition is more divergent compared to DAT and NET, resulting in a distinct S1 binding site environment. Comparison of the S1 binding sites of DAT and SERT reveals several key amino acid differences. Given many of these residues are implicated in ligand binding these differences may explain the distinct selectivity profiles of the 1,2-diarylethylamine series; V152^TM3^, F155^TM3^, and A480^TM10^ in DAT are substituted by residues I172^TM3^, Y175^TM3^, and T497^TM10^ in the SERT (Figure 4D). A recent study on the DAT/NET selective reuptake inhibitor methylphenidate, suggests that steric clashing between SERT S1 binding site residues on TM3, TM6a, TM10 and the methylphenidate molecule contributes to its relatively poor SERT activity.^87^ Given that the current bioisostere series exhibited similar IFD ligand–side chain interactions with DAT as seen with methylphenidate (Figure S3B, Supporting Information), we asked whether diphenidine—and, by extension, the other analogs—might encounter similar steric clashes. As illustrated in Figure 4D, overlaying the SERT protein structure (PDB: 7LIA) with the IFD pose of (*S*)-diphenidine at DAT; reveals steric clashes between the 1,2-diaryl moiety and the backbone regions of TM3 and TM6a of SERT, resembling those observed with methylphenidate.^87^ While suggestive, additional experimental data are needed to elucidate the structural basis of monoamine transporter selectivity observed with 1,2-diarylethylamines and other DAT/NET-selective reuptake inhibitors. In fact, mutagenesis of V152I and A480T in DAT failed to alter methylphenidate reuptake inhibition;^87^ however, multiple residues may act together or other residue differences outside the S1 binding site may alter the pocket structure between SERT and DAT and NET and influence ligand selectivity.

The fact that FuPP had reduced affinity for NMDAR binding relative to diphenidine but not for reuptake inhibition of DAT or NET is notable. These differences highlight the utility of bioisosteres in medicinal chemistry to retain a particular pharmacodynamic activity while changing another and exemplify the utility of phenyl ring bioisosteres for polypharmacological drug design. FuPP is notable given its relatively balanced pharmacological profile for NMDAR, DAT and NET and will be the subject of future investigations. Future studies will also explore the pharmacology of the individual enantiomers of these compounds. Based on existing SAR on diphenidine at NMDAR and phenylisopropylamines (e.g., amphetamine and methamphetamine) for DAT and NET reuptake inhibition, we anticipate the (*S*)-enantiomer is the eutomer with higher potency for both NMDAR and monoamine transporters.^25,92,93^ Understanding why furan for phenyl substitution reduced NMDAR, and not DAT or NET potency, could help inform future rational polypharmacological drug design approaches. In DAT and NET, the S1 binding site contains two aromatic residues (e.g., DAT: Y156^TM3^, F326^TM6b^; NET: Y152^TM3^, F323^TM6b^) which, based on the IFD studies, can make aryl–aryl interactions with the furan ring of FuPP and corresponding aryl rings on the bioisostere series (Figure 4B,C). In contrast, in the PCP binding site of NMDAR, no aromatic amino acid residues are present, and thus the bioisostere aryl rings do not make aryl–aryl interactions, but rather only weak VDW interactions with nonpolar residues (e.g., V644, A644, Figure 4A). One possibility to investigate is that the aryl–aryl interactions in DAT and NET are tolerant of the difference in ring polarity of the furan, whereas in NMDAR the increased polarity of the furan ring results in less favorable interactions with binding site residues. Consistent with this it is notable that in the NMDAR binding mode for FuPP, the oxygen of the furan projects up toward the extracellular region and is in the vicinity of the polar Thr-ring threonine residues (Figure S2F,L Supporting Information). A similar orientation is seen with TPP and SePP (see Figure S2G,I), however, the weaker electrostatic charge (weakly positive electrostatics rather than negative with O) and greater polarizability of the S and Se heteroatoms likely lead to more favorable interactions that more closely mimic those of the phenyl ring as in diphenidine. Potential differences in FuPP desolvation penalties between its binding at NMDAR vs the monoamine transporters are also worth investigating.

NMDAR antagonists have shown widespread clinical potential in a number of indications, including depression, neurodegenerative disease, acute and chronic pain, obsessive compulsive disorder, and epilepsies.^26–29,94^ We have previously observed that polypharmacology involving monoamine transporters, including NET, could improve NMDAR antagonist tolerability and, in some cases, enhance therapeutic efficacy.^25,61,71^ Because FXS is a neurodevelopmental disorder characterized by neuronal hyperexcitability, we considered FXS as a putative indication for the novel compounds.^50^ *Fmr1* KO mice exhibit AGS, which models seizure susceptibility and auditory hypersensitivity as well as neuronal hyperexcitability.^51,95^ Demonstrating the face validity of this model, most individuals with FXS exhibit auditory hypersensitivity, and seizures occur in ∼15% of individuals with FXS, and are more common in children than adults with FXS,^50^ mirroring the prevalence of AGS in *Fmr1* KO mice across their lifespan.^96,97^ We were also encouraged by the fact that NMDAR antagonists have demonstrated potent anticonvulsant activities in many preclinical in vivo models.^60–65^ Likewise, the NMDAR antagonist ketamine is routinely used off-label for control of various types of seizures including refractory status epilepticus.^30,31,98,99^ Consistent with enhanced network excitability, *Fmr1* KO mice were found to exhibit accelerated kindling development and prolonged electrographic seizures during amygdala kindling, and pharmacological inhibition of NMDARs with MK-801 attenuated the accelerated kindling rate.^100^ MK-801 and ketamine also reversed stereotyped behavior and social interaction deficits in another mouse model of autism.^101^ Additionally, DAT/NET inhibitors, including methylphenidate, are routinely used to treat attention deficits and impulsivity common in FXS.^102^ For these reasons, and our interest in the biological activity of the selenium analog, we tested SePP for its ability to prevent AGS in juvenile *Fmr1* KO mice. Although comparative in vivo evaluation across the bioisostere series would further inform relative advantages, the present studies were intentionally focused on SePP to establish proof of concept for a selenium-containing 1,2-diarylethylamine. As far as we are aware, this is the first experiment evaluating an uncompetitive NMDAR antagonist in AGS in *Fmr1* KO mice.

As shown in Figure 6, relative to vehicle, SePP, 10 mg/kg (N = 8, four male and four female mice), administered intraperitoneal (i.p.) prevented AGS in *Fmr1* KO mice, as measured by the abolishment of wild running and jumping (WRJ), tonic-clonic seizure (TCS), and lethality/respiratory arrest (RA) demonstrated by vehicle-treated mice (*P* < 0.0001 for WRJ and TCS, and P = 0.0013 for SC). SePP-treated mice showed normal behavior, like that of mice unexposed to the AGS stimulus, including exploring the cage, rearing, grooming, and interacting with cage-mates during testing. Results for the AGS test were analyzed by two-way Fisher’s exact tests. For ethical and statistical reasons, reported data from vehicle-treated subjects (N = 91) included historical and new observations (N = 3).^103^ All three vehicle-treated mice tested from the same litter as the subjects treated with SePP showed AGS, consistent with the historical data. Notably, we have observed no changes in AGS prevalence across time of day, season, or specific postnatal day among subjects aged *P*23–P25.

**Figure 6:**
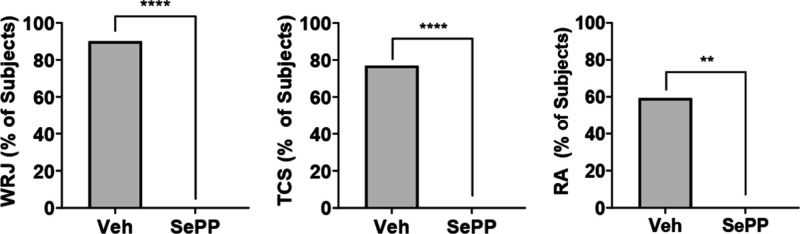
SePP, 10 mg/kg, protects *Fmr1* KO mice from audiogenic seizures. ** *p* < 0.01, **** *p* < 0.0001; WRJ = wild running and jumping; TCS = tonic-clonic seizure; RA = respiratory arrest.

These results demonstrate that SePP is protective against AGS in *Fmr1* KO mice and provide evidence that SePP could have clinical utility in FXS. Neuronal hyperexcitability is an overarching phenotype in the brains of *Fmr1* KO mice and patients with FXS^97,104^ Glutamate receptor inhibitors, specifically mGluR5 negative allosteric modulators (NAMs), have been investigated clinically for treating various symptoms of FXS. Unfortunately, none of the large clinical studies met therapeutic end points,^105,106^ though, a recent study showed that the mGluR5 NAM, mavoglurant, improved eye gaze behavior and impacted sympathetic nervous system reactivity to faces in patients with FXS.^107^ Although the clinical relevance of NMDAR antagonism in FXS remains unclear, our findings, together with existing evidence implicating NMDARs^57–59^ support continued investigation.

A well-known challenge with the clinical use of NMDAR antagonists is the occurrence of dissociative and motor side effects that can occur at therapeutic doses.^62^ We have postulated that reuptake inhibition of monoamine transporters, such as those seen with SePP, counters motor impairment affording greater tolerability profiles at therapeutic doses.^61^ To assess the potential for SePP to induce motor impairment at the antiepileptic dose, an elevated beam test was used. Results for the elevated beam test (scoring criteria shown in Table S10, Supporting Information) were analyzed by multiple, two-tailed Mann–Whitney tests. As shown in Figure 7, relative to vehicle (N = 8, four male and four female mice), SePP at 10 mg/kg i.p. (N = 8, four male and four female mice) did not significantly alter the number of slips (P = 0.27) or falls (*P* > 0.99), and did not significantly alter overall motor performance (P = 0.25) relative to vehicle-treated controls. These findings demonstrate that SePP prevents AGS in *Fmr1* KO mice at a dose devoid of deleterious effects on motor performance. Together, these results support the therapeutic potential of SePP in FXS syndrome and warrant further investigation; while a comprehensive dose response and safety assessment was beyond the scope of the present study, SePP was well tolerated at the efficacious dose tested, with no overt adverse effects observed.

**Figure 7:**
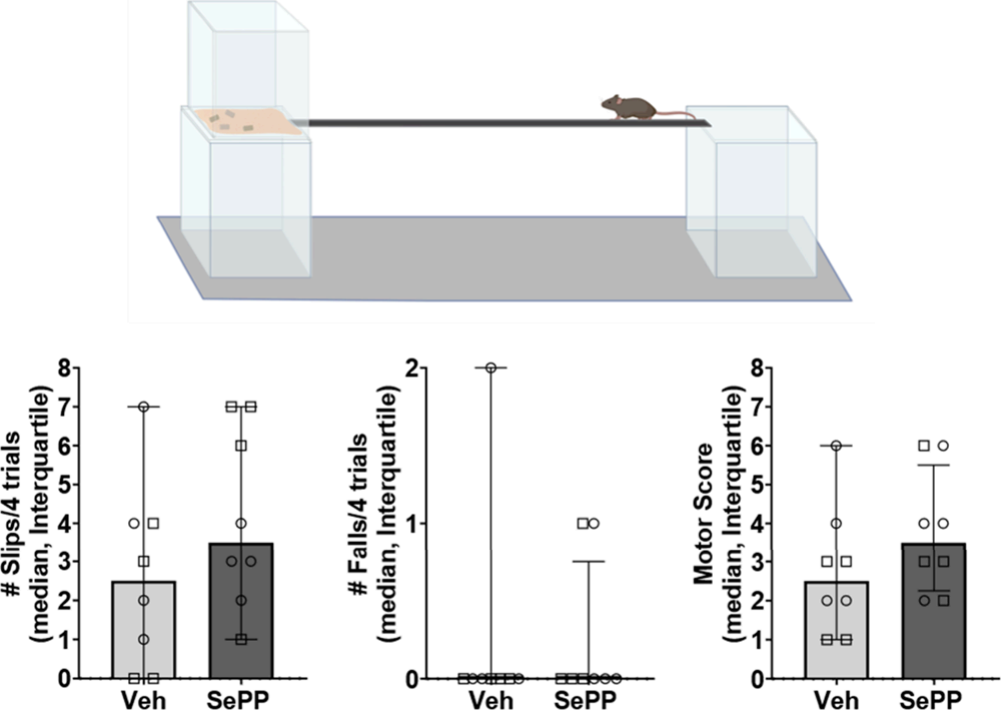
AGS anticonvulsant dose of SePP, 10 mg/kg, did not cause motor impairments in the elevated beam test in mice. Circles and squares represent female and male subjects, respectively.

To gain insight into the in vivo pharmacokinetics of SePP, a preliminary PK study was conducted in male and female mice. SePP concentrations were quantified in plasma and whole brain at 10 and 120 min post i.p. injection. The 10 min time point was selected because the auditory stimulus used to elicit audiogenic seizures lasts 5 min, resulting in a total interval of 10 min from injection to the end of behavioral testing. Individual animal data and summary statistics (mean ± SD) are reported in Table 2. Mean plasma concentrations of SePP were 387.5 and 33.8 ng/mL at 10 and 120 min, respectively, while mean whole-brain concentrations were 2,219.5 and 137.0 ng/mL at the corresponding time points. The brain and plasma concentrations measured at 10 min (mean plasma molar concentration at 10 min = 1.22 μM; see Tables S12 and S13, Supporting Information) are consistent with concentrations expected for pharmacological engagement of target NMDARs, DAT, and NET. Although a more comprehensive study incorporating additional time points will be required to determine full pharmacokinetic parameters (e.g., AUC, *C*_max_, *T*_max_, and elimination half-life, receptor occupancies, etc.), these data demonstrate fast absorption following i.p. administration with rapid systemic and brain exposure, followed by a >10-fold decline in both plasma and brain concentrations by 120 min. Like the AGS and elevated beam tests, the limited sample size (4 mice/time point) precluded reliable assessment of sex-dependent differences.

**Table 2:** In Vivo SePP Plasma and Whole-Brain Concentrations and K_p,brain_ in Mice at 10 and 120 min, Showing Individual Values and Mean ± SD

sample	plasma concentration (ng/mL)	whole brain concentration (ng/g)	K_p, brain_
mouse 1–10 min (male)	379.1	2166.4	5.7
mouse 2–10 min (male)	174.6	490.4	2.8
mouse 3–10 min (male)	358.7	2223.0	6.2
mouse 4–10 min (female)	637.7	3998.5	6.3
mean ± SD	387.5 ± 164.9	2219.5 ± 1240.7	5.3 ± 1.4
mouse 1–120 min (male)	30.3	116.1	3.8
mouse 2–120 min (male)	58.1	242.3	4.2
mouse 3–120 min (female)	21.4	95.7	4.5
mouse 4–120 min (female)	25.4	93.8	3.7
mean ± SD	33.8 ± 14.4	137.0 ± 61.5	4.1 ± 0.3

SePP exhibited robust brain penetration, with mean K_p,brain_ values of 5.25 and 4.04 at 10 and 120 min, respectively, indicating preferential partitioning into the brain relative to plasma. The observation of high whole-brain SePP levels at 10 min post-i.p administration is consistent with a central mechanism underlying the observed in vivo anti-AGS activity of SePP. The persistence of K_p,brain_ values >4 at both time points suggests that brain exposure is not solely driven by transient plasma levels and may reflect favorable physicochemical properties or CNS retention. Although total K_p,brain_ provides a practical index of brain partitioning, generally a limitation of these data is that K_p,uu,brain_, rather than total K_p,brain_, is the more mechanistically informative metric for BBB transport and CNS target exposure, as total K_p,brain_ is influenced by nonspecific plasma and brain binding.^108^ Unfortunately, comparator data on diphenidine is unavailable from literature, but a publicly available project report states that diphenidine was detected in rat brain dialysate following i.p. injection, with highest concentrations seen at 30 min.^109^ The 2-methoxy analog of diphenidine, 2-methoxyphenidine (2-MXP) was also evaluated in rats and showed similar high brain penetration with a max concentration seen at 30 min and an elimination half-life of 2.15 h.^110^ Future studies may compare the pharmacokinetic profile of SePP with that of diphenidine and other analogs to further contextualize brain exposure and clearance behavior within this structural class. Likewise, evaluating the in vitro (e.g., liver microsomes) and in vivo metabolism of SePP would further inform the metabolic behavior of selenophene-based compounds.

## Conclusions

A series of 1,2-diarylethylamine phenyl ring bioisosteres were designed, synthesized, and pharmacologically evaluated as NMDAR antagonists and monoamine transporter reuptake inhibitors. Generally, the series exhibited comparable potencies at these targets suggesting the 5-membered heteroaryl rings serve as phenyl ring bioisosteres within the scaffold for all target proteins. Notably, FuPP showed a ∼4-fold reduction in NMDAR affinity which could be due to a distinct electrostatic surface and/or the relatively smaller ring volume impacting binding. In silico docking studies suggest that the compounds engage the target protein binding sites in a broadly similar manner, forming conserved interactions with key residues of DAT, NET, and NMDAR that are known to contribute to inhibitor binding. Given both the interest in incorporating nontraditional atoms like selenium into drug design and the emerging role of NMDAR hyperactivity in FXS pathology, we evaluated a selenium-containing NMDAR antagonist/DAT and NET reuptake inhibitor, SePP in vivo. In *Fmr1* KO mice, a model of FXS, SePP demonstrated potent anti-AGS activity at a dose that did not impair motor performance in the elevated beam test. Overall, these findings identify several novel 1,2-diarylethylamines incorporating phenyl ring bioisosteres and provide new insights that advance structure–activity relationship (SAR) development for this chemical class at NMDAR, DAT, NET and SERT and support further investigations into the therapeutic potential of SePP in the treatment of auditory hypersensitivity and seizures in FXS patients.

## Experimental Section

### Reagents

Starting materials, reagents and solvents used for synthesis were generally 95% pure or greater and were obtained from Sigma-Aldrich (St Louis, MO, USA), Alfa Aesar (Tewksbury, MA, USA), Oakwood Chemical (Estill, SC, USA), 1PlusChem (San Diego, CA, USA), 1ClickChemistry Inc (Allen, TX, USA), and VWR (Radnor, PA, USA). APP^+^, (+)-MK-801 hydrogen maleate, citalopram HBr, desipramine HCl, and GBR-12935 di-HCl were obtained from MedChem Express (USA) in >99% purity.

### Melting Point Determination

Melting points (uncorrected) were determined using a Digimelt A160 SRS melting point apparatus (Stanford Research Systems, Sunnyvale, CA, USA) at a ramp rate of 2 °C/min.

### Nuclear Magnetic Resonance Spectroscopy

^1^H NMR (400 MHz) and ^13^C (101 MHz) spectra were obtained on 20 mg/mL solutions of the hydrochloride salts in anhydrous *d*_6_-DMSO (>99.9% D, Sigma-Aldrich) on a Bruker Ultrashield 400 plus spectrometer with a 5 mm BSO S1 (Z gradient plus) probe at ambient temperature.

### High-Performance Liquid Chromatography (HPLC)

An Agilent 1260 Infinity system was used that is comprised of a 1260 quaternary pump VL, a 1260 ALS autosampler, a 1260 Thermostated Column Compartment, and a 1200 DAD Multiple Wavelength Detector (Agilent Technologies, CA, USA). A detection wavelength of 220 nm was used to estimate purity. A Zorbax Eclipse Plus-C18 analytical column (5 μm, 4.6 × 150 mm) from Agilent (Agilent Technologies, Santa Clara, CA, USA) was used for chromatographic separation of the compounds. Mobile phase A consisted of a 10 mM aqueous ammonium formate buffer which was prepared in HPLC-grade water titrated to pH 4.5 (using a 10 mM formic acid solution). Mobile phase B consisted of HPLC-grade acetonitrile (ACN). Compounds were prepared at 2.0 mg/mL in the mobile phase they were run in. An injection volume of 40 μL was used, flow rate was 1.0 mL/min, and column temperature was set to 25 °C.

### High-Resolution Mass Spectrometry (HRMS)

HRMS experiments were performed on a Thermo Orbitrap Exactive Mass Spectrometer with an Orbitrap mass analyzer, calibrated using electrospray ionization with Pierce LTQ ESI Positive Ion Calibration Solution (ThermoFisher Scientific, USA). The following measurement parameters were used: Aux gas flow rate-8, Spray Voltage-3.50 kV, Capillary temperature 275 °C, Capillary Voltage 25.00 V, Tube Lens Voltage 65.00 V, Skimmer Voltage 14.00 V, Heater Temperature 100 °C. Samples (HCl salts) were analyzed via an Atmospheric Solids Analysis Probe (ASAP) source.

### Biotage Flash System

Flash column chromatography purifications were performed using a Biotage Isolera One Flash Chromatograph with Spektra UV detection (254 and 280 nM). KP-Sil 50 and 100 g cartridges were manually packed using 230–400 mesh, 60 Å silica gel (Sigma-Aldrich, St Louis, MO).

### Pharmacology

#### NMDAR Competitive Radioligand Binding Studies

Rat forebrain homogenate from 7 to 8 week old Sprague–Dawley rat brain tissue (BioChemed, USA) were prepared as described previously.^111,112^ Rat forebrains were homogenized in 10 mM HEPES and 1 mM EDTA buffer (pH 7.4) using a mechanical homogenizer (Janke-Kunkel Ultra-Turrax T25). The brain homogenate was centrifuged (15 min at 20,000 rpm and 4 °C). The supernatant was removed and the pellet was resuspended in 10 mM HEPES and 1 mM EDTA buffer (pH 7.4) and this was repeated 5 times. After the fifth centrifugation, the pellet was resuspended in 10 mM HEPES (pH 7.4) and incubated in a 37 °C water bath in the dark. Centrifugation and resuspension was repeated 3 more times. The final resuspended homogenate was aliquoted and stored in a −80 °C freezer until needed. Protein concentration was quantified via the Bradford method using Coomassie Protein Assay Reagent (Sigma, USA) with bovine serum albumin (Sigma, USA) as standard.

To each well of a 96 DeepWell Plate (Thermo Scientific, USA) was added glutamate (100 μM) and glycine (10 μM), followed by test compound, NSB control (30 μM MK-801) or TB control (buffer), (+)-[^3^H]-MK-801 (PerkinElmer NET972250UC) (1 nM final concentration) and finally protein homogenate (100 μg/mL) in 10 mM HEPES buffer (pH = 7.4). Total volume of 1 mL. The plates were incubated at 25 °C, covered with aluminum foil, and placed on a mechanical shaker (250 rpm) for 2 h. After 2 h, homogenate with bound (+)-[^3^H]-MK-801 was collected by vacuum filtration using a Unifilter-96 Cell Harvester (PerkinElmer) over prewet UniFilter-96 GF/C P Microplates (PerkinElmer) and filters were washed with room temperature 10 mM HEPES buffer (pH 7.4) (3 × 1 mL). Filter plates were dried overnight in a fume hood and the next day, MicroScint-O (PerkinElmer) was added and tritium counted via liquid scintillation counting using a MicroBeta2 Plate Reader with 6-detectors scintillation counter (PerkinElmer) at 55% efficiency. IC_50_ value estimates were determined in GraphPad Prism 9.3.1 using nonlinear regression (single site fit) with log-concentration plotted against percent specific binding. K_i_ values were calculated using the equation of Cheng and Prusoff. The K_d_ for (+)-MK-801 (1.747 nM), was determined previously via homologous binding experiments.^67^ Binding experiments were performed in duplicate and repeated 3 times. Each plate contained a full (+)-MK-801 concentration response experiment as a control.

#### Fluorescent Monoamine Transporter Inhibition Functional Studies

Cell lines stably expressing Serotonin (SERT), Dopamine (DAT), and Norepinephrine (NET) human monoamine transporter proteins, were purchased from PerkinElmer (SERT: HEK293 Cat: RBHSTM-K and DAT: CHO-K1 Cat: RBHDATM-K) and BPS Biosciences (NET: CHO-K1 60557). Cells were cultured in 150 mm Tissue Culture dishes (Cell Treat, USA) at 37 °C and 5% CO_2_ according to the manufacturer’s recommendations.

On day 0, cells were detached from their growth dishes using Trypsin-EDTA (0.25%) (Gibco 25200–056) at 70–80% confluency, and seeded at 60,000 cells/well into clear bottom, black-walled 96 well plates (Corning, USA). For the HEK293 SERT cells, wells were coated with poly-d-lysine (Gibco, USA) for adherence. Krebs-Ringer HEPES (KRH) Buffer at pH 7.4 was prepared fresh. This included 10 mM HEPES, 130 mM NaCl, 1.3 mM KCl, 2.2 mM CaCl_2_, 1.2 mM MgSO_4_, 1.2 mM KH_2_PO_4_. pH was adjusted using 6N NaOH (Sigma, USA).

DMSO stock solutions of APP^+^, and test compounds were diluted using a 10-fold serial dilution format in KRH buffer containing a consistent amount of DMSO (1% in 4X stock, final % in assay: 0.25%). Test compounds were evaluated at 7 concentrations from 10^–10^ to 10^–4^. For each transporter an excess concentration of the corresponding known inhibitor (400 μM final concentration) served as a nonspecific (NS) control representing background and any nonspecific fluorescence present with maximal transporter inhibition. These were citalopram: SERT, Desipramine: NET, and GBR-12935: DAT. KRH buffer served as a total fluorescence control to represent the maximum amount (100%) of substrate uptake without transporter inhibition.

For the assay, media was removed from the wells via aspiration and 100 μL of KRH buffer added to all wells. Solutions of controls, test compounds, nonspecific and total fluorescence as described above, were then added to the plate (50 μL of 4X stock) in triplicate. To each well was added 50 μL of 40 μM APP^+^ in KRH buffer, bringing the total well volume to 200 μL. Wells were protected from light exposure and incubated in the 37 °C incubator (5% CO_2_) for 1 h. Then to quench extracellular APP^+^ fluorescence, 20 μL of 330 μM of a Trypan Blue (Sigma, USA) in KRH solution, was added to each well. Fluorescence (excitation 430 nm and emission at 510 nm) was read (bottom read) using a microplate reader (BioTek Synergy Neo 2) set to 37 °C. A brief orbital shake was used prior to reading the plate. Data were analyzed using GraphPad Prism 9.3.1 via nonlinear regression with log-concentration plotted against percent specific fluorescence (total - NS) and half-maximal inhibitory constants, (IC_50_) estimates calculated. Concentration response experiments were repeated three times to calculate a mean ± SEM of the IC_50_ estimate. Full dose response curves were generally not performed for test compounds that failed to show >50% inhibition at 10 μM concentration (N = 2). Each assay plate contained a control concentration response with a known inhibitor. Citalopram: SERT, Desipramine: NET, and GBR-12935: DAT.

#### Audiogenic Seizures (AGS)

All in vivo experimental activities were approved by Mercer University’s IACUC after a review assessing the safety and humane use of study subjects (IACUC protocol number A2305003) and were performed in accordance with the Guide for the Care and Use of Laboratory Animals (eighth edition). Experiments testing the induction of AGS in male and female *Fmr1* KO mice (FVB.129P2-*Pde6b* ^*+*^ *Tyr* ^*c‑ch*^ *Fmr1* ^*tm1Cgr*^/J, stock #004624, Jackson Laboratory) were conducted as previously described.^96,113^ Mice were housed in groups of 3–5 in clear polycarbonate cages with mesh wire lids and corn cob bedding (Bed-o’Cobs 1/8″, Stewart’s feed service, Inc.). The colony rooms were temperature and humidity-controlled (20–25 °C and 45–55%, respectively) with the background noise level of 50–55 dB. Mice were exposed to a 12h light/dark cycle with ad libitum access to food (Rodent Diet 5001, Stewart’s feed service, Inc.) and water. On the day of the experiment, test subjects, aged *P*23–P25, were acclimated to the test room for >30 min in their home cages. Mice were then injected intraperitoneally (i.p.) with vehicle or SePP 10 mg/kg (10 mL/kg). All subjects were returned to their cages and tested 5 min after injection. Mice were placed in a clear, polycarbonate test box (46 cm × 20 cm × 20 cm) covered with a perforated, clear, polycarbonate lid 1 min before being exposed to an alarm (RadioShack Kit #49–1010, doorstop alarm). The alarm, at ∼115 dB, was held by hand ∼10 cm from the test box, and the exposure duration was 5 min. A sound level meter/data logger (REED Model SD-4023) was placed ∼20 cm from the alarm and read during testing to ensure a uniform sound pressure level in each experiment. Up to 4 mice (2 per test box) were observed simultaneously.^114^ The average (±standard deviation (SD)) baseline sound pressure in the testing room was 55 ± 9 dB, and the average alarm sound pressure was 115 ± 4 dB.

Behavioral responses, including normal behavior, WRJ, TCS, and RA were documented during AGS testing. Normal behavior was defined as coordinated locomotion, alertness, exploring, sniffing, sitting, rearing, grooming, socializing, startle and eye squinting. The full sequelae of AGS was a startle response and eye squinting, followed by WRJ phase(s), brief opisthotonos, a clonic phase with the mouse lying on either side of its body with head, neck, trunk, and limbs ventro-flexed (muscle jerking and twitching with rigidity), a short (∼5 s) tonic seizure phase with full extension of extremities (muscle stiffening) and finally RA. In the case of recovery from the TCS phase, mice exhibited a second round of WRJ, Straub tail, a full body vibrating shudder, and tremors which finally ended with either freezing or a transition to normal behavior. Behaviors were video-recorded using a high-definition camcorder (Vixia HF R800, Canon) for data analyses after testing.^114^

#### Elevated Beam Test (EBT)

The EBT^115^ is a behavioral test used to assess motor ability and coordination preclinically and is amenable to evaluating motor effects of xenobiotics. We used the EBT to evaluate the motor effects of SePP compared to vehicle in adult male and female WT mice (controls for *Fmr1* KO mice, FVB.129P2-*Pde6b* ^*+*^ *Tyr* ^*c‑ch*^/AntJ, stock #004828, Jackson Laboratory). An elevated beam (1 cm wide, 1-m-long steel bar) was placed 22 cm above the ground, between two clean, standard rat boxes (43 cm by 20 cm). An additional rat box was placed sideways at the end of the beam and contained food and bedding from the subject’s home cage. See Figure 7 for a representation of the setup. On the training day and testing day, subjects were brought to the procedure room and were acclimated there for 60 min in their home cages. Subjects were then acclimated in the start box for three min and then trained to traverse the beam three times from three different starting points in this order: near the end (5 cm), in the center (50 cm), and at the beginning of the beam. During training, after subjects crossed the beam into the end box, they were allowed to explore it for 60 s. If subjects fell during training, they were placed at the end cage for an additional minute before resuming training from where they fell. Each training trial lasted a maximum of 15 min, and all subjects learned to cross the beam in less than 5 trials from each of the 3 starting points. After training, mice were returned to their home cages. Twenty-4 h later, mice were returned to the procedure room, weighed, and randomly assigned to receive an i.p. injection of either vehicle or SePP 10 mg/kg (10 mL/kg). Five min after injection, mice were tested for their performance on the EBT. Each mouse was tested on four consecutive trials, and the number of paw slips and falls were recorded. A foot slip was defined as paw misplacement where digits slipped off or entirely missed the ledge of the beam, causing a slouch in the body posture of the mouse. Overall motor performance was scored based on the scale presented in Table S10, Supporting Information.

### LC-MS/MS Instrument

The LC-MS/MS system consists of an Agilent 1100 Series HPLC system (Agilent, Santa Clara, CA, USA) with a G1312A binary pump, G1313A autosampler (ALS), and G1316A thermostated column compartment (COLCOM). The LC system is coupled to a Thermo Scientific TSQ Quantum Access triple quadrupole mass spectrometer (Thermo Scientific, Waltham, MA, USA) equipped with a heated electrospray ionization (HESI) source. The LC and MS components were controlled using Thermo Xcalibur software (version 4.0.27.42, Thermo Scientific).

#### Pharmacokinetic Study in Mice

Adult male and female FVB.129P2-*Pde6b* ^*+*^ *Tyr* ^*c‑ch*^/AntJ Tyrc-ch/AntJ mice (wild-type, JAX stock #004828),were brought in their home cages to a procedure room and acclimated for >30 min. Following acclimation, subjects were injected intraperitoneal (i.p.) with vehicle (Milli-Q water for blank measurements) or SePP 10 mg/kg (dissolved in Milli-Q water). At specified time points after injection (5 min for vehicle, 10 and 120 min for SePP), subjects were anesthetized with isoflurane, then decapitated. Trunk blood (∼500 μL) was collected in K2-EDTA-containing vials (BD Microcontainer) prechilled on ice, and brains were rapidly dissected and flash frozen in isopentane on dry ice. Plasma was isolated from whole blood by centrifugation at 2,900 rpm for 20 min at 4 °C. After collection, brains and plasma were stored at −80 °C.

### Sample Preparation and Analysis

Whole mouse brains were weighed, then homogenized in four volumes (w/v) of 10 mM ammonium formate buffer (pH 4.5), aliquoted, and refrozen. Twenty μL of thawed plasma or brain homogenate was diluted 1:5 (v/v) in MeOH containing IS (625 ng/mL) in a 1.5 mL low retention Eppendorf tube, vortexed (40 s) and then spun down at 13,000 g for 15 min. The supernatant (50 μL) was diluted 1:5 (v/v) in mobile phase, and 10 μL was injected for analysis. Analyte samples were prepared as technical replicates and averaged. SePP standards were prepared via serial dilutions in plasma or whole brain matrix from vehicle-treated mice. Additional details on standards, including concentrations, are provided in the supplemental document (Tables S11–S14). Calibration curves were generated by weighted (1/x) linear regression of analyte-to-internal standard peak area ratios versus concentration. Double blank and zero blanks were included. Data was analyzed using GraphPad prism (version 10.2.0). K_p,brain_ was calculated by dividing the whole brain (ng/g) by the plasma concentration (ng/mL).

### LC-MS/MS Conditions

LC-MS/MS analyses were performed on a Zorbax Eclipse XDB-C18 analytical column (5 μm, 4.6 × 150 mm, Agilent, Santa Clara, CA, USA) equipped with an Eclipse XDB-C18 analytical guard column (5 μm, 4.6 × 12.5 mm, Agilent, Santa Clara, CA, USA). Mobile phase A consisted of 10 mM ammonium formate buffer at pH 4.5 and mobile phase B consisted of LC-MS/MS grade MeOH (Sigma-Aldrich, St. Louis, MO, USA). Samples were run isocratic using A:B of 15:85, with a flow rate of 0.250 mL/min, a column temperature of 27 °C and a 20 min run time. Data was acquired in selected reaction monitoring (SRM) mode in positive mode. Source parameters were: spray voltage (3.9 kV), dwell time (100 ms), vaporizer temperature (300 °C), capillary temperature (350 °C), sheath gas pressure (35 units), auxiliary gas pressure (10 units), and argon collision gas pressure (1.5 mTorr). Mass resolutions of 0.7 for peak width were used in Q1 and Q3. Analyte and diphenidine internal standard (IS) specific parameters are listed in Table S15.

### Syntheses of Target Compounds

The 5-membered heterocycle-containing target compounds were synthesized via a two-step one-pot organozinc modified Mannich reaction which was adapted from literature.^116^ The major deviation was implementation of a preformation step for the benzyl zinc bromide nucleophilic species (e.g., non-Barbier conditions), as well as optimization of work up conditions.^66^ These modifications have been observed to consistently improve yields in our hands.^66,67^ For the pharmacological characterizations compounds were obtained as the hydrochloride salts in >95% purity determined using ^1^H NMR and HPLC.

#### Preparation of 1-[1-(Furan-2-yl)-2-phenylethyl]­piperidine (FuPP)

To a dry vessel under argon was added 40 mL of dry (3Å molecular sieves) THF, zinc dust (<10 μm, 3.53 g, 54.0 × 10^–3^ mol), and trifluoroacetic acid (450 μL, 5.9 × 10^–3^ mol) while stirring. This was allowed to react for 5 min under argon. Next, benzyl bromide (8.04 g, 5.58 mL, 47.0 × 10^–3^ mol) was carefully (exothermic reaction) added with vigorous stirring. The resulting reaction was kept under argon flow to allow pressure venting for 15 min, at which point it recovered to ambient temp. Next, piperidine (2.20 g, 2.6 mL, 25.9 × 10^–3^ mol) and furfural (2.26 g, 2.0 mL, 23.5 × 10^–3^ mol) were added in rapid succession and the reaction sealed under argon and stirred overnight. The reaction was then carefully poured into 300 mL of 1 M HCl solution and washed with EtOAc (2 × 75 mL). The organic washes were pooled and extracted with 1 M HCl (3 × 75 mL). Acidic aqueous phases were pooled and made basic with 28% NH_4_OH to pH > 10 and extracted with EtOAc (3 × 100 mL). The pooled organic phases were washed with brine (∼30 mL), pooled, dried over anhydrous Na_2_SO_4_ and the solvent removed under vacuum to yield 3.78 g of the crude freebase, as a dark brown oil. The crude freebase material was purified via flash column chromatography (silica gel, hexanes containing 1% TEA) to yield FuPP as a light brown-tinted transparent oil (3.73 g, 14.6 × 10^–3^ mol, 62.1% yield). This material was converted to the HCl salt from concentrated HCl (1.1 mol equiv) in acetone. The solvent was then evaporated under a stream of warm air. Additional acetone was added and the cycle repeated (3X) to drive off excess HCl and moisture, ultimately yielding off-white crystalline solids. These solids were crystallized (3X) by dissolving in 50 mL boiling acetone, adding 50 mL Et_2_O and storing overnight in a −20 °C freezer. After washing and drying solids were further dried via high vacuum to yield FuPP HCl as thin transparent-crystalline needles (mp 221.2–221.9 °C). ^1^H NMR (400 MHz, *d*_6_-DMSO) δ 11.23 (s, 1NH^+^), 7.78 (dd, *J* = 1.9, 0.8 Hz, 1H), 7.21 (att, *J* = 7.1, 1.5 Hz, 2H), 7.18–7.13 (m, 1H), 7.11 (ad, *J* = 7.7 Hz, 2H), 6.71 (d, *J* = 3.0 Hz, 1H), 6.48 (dd, *J* = 3.3, 1.9 Hz, 1H), 4.69 (dd, *J* = 12.2, 1.7 Hz, 1H), 3.69 (dd, *J* = 12.8, 2.6 Hz, 1H), 3.55 (d, *J* = 11.3 Hz, 2H), 3.43 (t, *J* = 12.7 Hz, 1H), 2.66–2.52 (m, 2H), 2.06–1.85 (m, 2H), 1.84–1.73 (m, 2H), 1.66 (d, *J* = 12.4 Hz, 1H), 1.37–1.21 (m, 1H). ^13^C NMR (101 MHz, *d*_6_-DMSO) δ 145.0 (1C), 145.0 (1C), 136.3 (1C), 128.9 (2C), 128.4 (2C), 126.8 (1C), 114.7 (1C), 111.0 (1C), 63.2 (1C), 51.8 (1C), 47.7 (1C), 33.8 (1C), 22.5 (2C), 21.5 (1C). HRMS (ASAP): [M + H]^+^ theoretical for C_17_H_22_NO^+^ *m*/*z* 256.1696, observed 256.1688, Δppm = −3.1. HPLC Purity (7:3 Buffer:ACN, 220 nm): 99.0%, RT: 3.83 min.

#### Preparation of 1-(1,2-Diphenylethyl)­piperidine (Diphenidine)

Diphenidine was prepared as described above for FuPP using benzaldehyde (0.93 g, 0.90 mL, 8.77 × 10^–3^ mol), piperidine (0.82 g, 0.95 mL, 9.65 × 10^–3^ mol), benzyl bromide (3.00 g, 2.09 mL, 17.5 × 10^–3^ mol), zinc dust (1.15 g, 17.5 × 10^–3^ mol), and trifluoroacetic acid (168 uL, 2.19 × 10^–3^ mol) in 30 mL of THF as the starting materials. The crude free base (yellow oil) was purified via flash column chromatography using a gradient of hexanes:EtOAc (6:1 to 3:1) containing 1% triethylamine (TEA) to give diphenidine (1.93 g, 7.25 × 10^–3^ mol, 82.7%) as a clear oil. The HCl salt was prepared as described for FuPP as white crystalline solids (mp 208.6–210.4 °C). ^1^H NMR (400 MHz, *d*_6_-DMSO) δ 11.27 (s, 1NH^+^), 7.62–7.50 (m, 2H), 7.41–7.31 (m, 3H), 7.14 (at, *J* = 7.5 Hz, 2H), 7.10–7.06 (m, 1H), 7.04 (ad, *J* = 7.4 Hz, 2H), 4.62 (dt, *J* = 12.1, 3.5 Hz, 1H), 3.80 (d, *J* = 12.9 Hz, 1H), 3.73 (d, *J* = 11.8 Hz, 1H), 3.51 (t, *J* = 12.6 Hz, 1H), 3.39 (d, *J* = 12.1 Hz, 1H)*, 2.68–2.52 (m, 2H)**, 2.13–1.96 (m, 1H), 1.96–1.81 (m, 1H), 1.81–1.71 (m, 2H), 1.65 (d, *J* = 13.0 Hz, 1H), 1.26 (qt, *J* = 13.0, 3.8 Hz, 1H). *coalescence with H_2_O, **coalescence with DMSO. ^13^C NMR (101 MHz, *d*_6_-DMSO) δ 136.6 (s, 1C), 131.5 (s, 1C), 130.6 (s, 2C), 129.4 (s, 1C), 129.1 (s, 2C), 128.6 (s, 2C), 128.2 (s, 2C), 126.4 (s, 1C), 70.0 (s, 1C), 51.9 (s, 1C), 48.4 (s, 1C), 35.0 (s, 1C), 22.3 (s, 2C), 21.6 (s, 1C). HRMS (ASAP): [M + H]^+^ theoretical for C_19_H_24_N^+^ *m*/*z* 266.1903, observed *m*/*z* 266.1905, Δppm = 0.8 ppm. HPLC Purity (7:3 Buffer:ACN, 220 nm): 100%, RT: 5.19 min.

#### Preparation of 1-[2-Phenyl-1-(thiophen-2-yl)­ethyl]­piperidine (TPP)

TPP was prepared as described above for FuPP using 2-thiophenecarboxaldehyde (2.36 g, 1.96 mL, 21.0 × 10^–3^ mol), piperidine (1.97 g, 2.29 mL, 23.1 × 10^–3^ mol), benzyl bromide (7.18 g, 4.99 mL, 42.0 × 10^–3^ mol), zinc dust (3.16 g, 48.3 × 10^–3^ mol), and trifluoroacetic acid (402 μL, 5.3 × 10^–3^ mol) in 40 mL of THF as the starting materials. The crude freebase (viscous dark yellow oil) was purified via flash column chromatography using a gradient of hexanes:EtOAc (19:1 to 5:1) containing 1% triethylamine (TEA) to give TPP (4.70 g, 17.3 × 10^–3^ mol, 82.4% yield) as a golden yellow transparent oil. The HCl salt was prepared as described for FuPP as transparent thin crystalline needles (mp 181.9–184.8 °C). ^1^H NMR (400 MHz, *d*_6_-DMSO) δ 11.46 (s, 1NH^+^), 7.65 (dd, *J* = 5.1, 1.0 Hz, 1H), 7.37 (dd, *J* = 3.5, 1.0 Hz, 1H), 7.23–7.16 (m, 2H), 7.16–7.10 (m, 1H), 7.14 (ad, *J* = 6.9 Hz, 2H), 7.07 (dd, *J* = 5.1, 3.6 Hz, 1H), 4.97 (dt, *J* = 12.2, 2.8 Hz, 1H), 3.87 (dd, *J* = 13.0, 2.3 Hz, 1H), 3.63 (d, *J* = 11.6 Hz, 1H), 3.54 (d, *J* = 11.6 Hz, 1H), 3.37 (t, *J* = 12.6 Hz, 1H), 2.68 (qd, *J* = 11.5, 2.9 Hz, 1H), 2.58 (dq, *J* = 11.4, 2.6 Hz, 1H), 2.10–1.86 (m, 2H), 1.86–1.72 (m, 2H), 1.66 (ad, *J* = 13.2 Hz, 1H), 1.27 (qt, *J* = 13.1, 3.8 Hz, 1H). ^13^C NMR (101 MHz, *d*_6_-DMSO) δ 136.5 (1C), 133.1 (1C), 131.8 (1C), 129.0 (2C), 128.9 (1C), 128.3 (2C), 127.4 (1C), 126.6 (1C), 64.7 (1C), 51.9 (1C), 47.2 (1C), 37.1 (1C), 22.5 (2C), 21.6 (1C). HRMS (ASAP): [M + H]^+^ theoretical for C_17_H_22_NS^+^ *m*/*z* 272.1467, observed *m*/*z* 272.1458, Δppm = −3.3 ppm. HPLC Purity (7:3 Buffer:ACN, 220 nm): 97.4%, RT: 5.06 min.

#### Preparation of 1-[2-Phenyl-1-(thiophen-3-yl)­ethyl]­piperidine (3-TPP)

3-TPP was prepared as described above for FuPP using 3-thiophenecarboxaldehyde (0.41 g, 0.32 mL, 3.57 × 10^–3^ mol), piperidine (0.34 g, 0.39 mL, 3.93 × 10^–3^ mol), benzyl bromide (1.22 g, 0.85 mL, 7.14 × 10^–3^ mol), zinc dust (0.47 g, 7.14 × 10^–3^ mol), and trifluoroacetic acid (70 μL, 0.89 × 10^–3^ mol) in 80 mL of ACN as the starting materials. This crude freebase material (slight yellow-tinted transparent oil) was purified utilizing column chromatography with silica gel and eluted with a mixture of hexanes and EtOAc (5:1) containing 1% TEA to give a transparent colorless oil as the pure product, 3-TPP (0.55 g, 2.02 × 10^–3^ mol, 56.5% yield). The HCl salt was prepared as described for FuPP and collected as an off-white crystalline solid (mp 176.7–177.4 °C). ^1^H NMR (400 MHz, *d*_6_-DMSO) δ 11.19 (s, 1NH^+^), 7.72 (dd, *J* = 2.9 Hz, 1.1 Hz, 1H), 7.60 (dd, *J* = 5.0, 2.9 Hz, 1H), 7.40 (dd, *J* = 5.0, 1.2 Hz, 1H), 7.17 (att, *J* = 7.1, 1.4 Hz, 2H), 7.14–7.10 (m, 1H), 7.09 (ad, *J* = 7.7 Hz, 2H), 4.70 (dt, *J* = 12.2, 2.9 Hz, 1H), 3.73 (dd, *J* = 12.8, 2.5 Hz, 1H), 3.62 (d, *J* = 11.7 Hz, 1H), 3.46 (t, *J* = 12.6 Hz, 1H)*, 3.53–3.38 (m, 2H)*, 2.62–2.51 (m, 2H), 2.00 (qt, *J* = 13.5, 3.9 Hz, 1H), 1.89 (qt, *J* = 13.4, 3.6 Hz, 1H), 1.83–1.72 (m, 2H), 1.71–1.60 (m, 1H), 1.25 (qt, *J* = 12.9, 3.8 Hz, 1H). *Coalescing. ^13^C NMR (101 MHz, *d*_6_-DMSO) δ 136.8 (1C), 132.3 (1C), 129.1 (2C), 128.9 (1C), 128.7 (1C), 128.2 (2C), 127.1 (1C), 126.5 (1C), 65.0 (1C), 51.8 (1C), 47.7 (1C), 35.6 (1C), 22.5 (2C), 21.6 (1C). HRMS (ASAP): [M + H]^+^ theoretical for C_17_H_22_NS^+^ *m*/*z* 272.1467, observed *m*/*z* 272.1457, Δppm = −4.0 ppm. HPLC Purity (7:3 Buffer:ACN, 220 nm): 99.1%, RT: 4.51 min.

#### Preparation of 1-[2-Phenyl-1-(selenophen-2-yl)­ethyl]­piperidine (SePP)

SePP was prepared as described above for FuPP using 2-formylselenophene (1.00 g, 6.30 × 10^–3^ mol), piperidine (0.682 mL, 6.88 × 10^–3^ mol), benzyl bromide (2.14 g, 1.50 mL, 12.5 × 10^–3^ mol), zinc dust (0.940 g, 14.38 × 10^–3^ mol), and trifluoroacetic acid (120 μL, 1.56 × 10^–3^ mol) in 40 mL of dry THF. The crude freebase was obtained as a viscous amber oil (1.53 g) and purified utilizing column chromatography with silica gel and eluted with a mixture of hexanes with 3% EtOAc containing 1% TEA and then converted to the HCl salt as described for FuPP and collected as clusters of light yellow transparent needles (0.810 g, 2.29 × 10^–3^ mol, 36.3% yield) (mp 177.3–179.0 °C). [HCl] ^1^H NMR (400 MHz, *d*_6_-DMSO) δ 11.41 (s, 1NH^+^), 8.29 (dd, *J* = 5.6, 1.2 Hz, 1H), 7.45 (dd, *J* = 3.8, 1.2 Hz, 1H), 7.22 (dd, *J* = 5.4, 3.7 Hz, 1H), 7.21–7.10 (m, 5H), 5.01 (dt, *J* = 12.2, 3.2 Hz, 1H), 3.87 (dd, *J* = 13.2, 2.9 Hz, 1H), 3.66 (d, *J* = 12.2 Hz, 1H), 3.52 (d, *J* = 12.1 Hz, 1H), 3.29 (t, *J* = 12.5 Hz, 1H), 2.75 (qd, *J* = 9.2, 3.2 Hz, 1H), 2.65 (qd, *J* = 9.3, 3.2 Hz,, 1H), 2.11–1.89 (m, 2H), 1.88–1.74 (m, 2H), 1.68 (d, *J* = 13.2 Hz, 1H), 1.29 (qt, *J* = 13.0, 3.8 Hz, 1H). ^13^C NMR (101 MHz, *d*_6_-DMSO) δ 140.1 (1C), 136.5 (1C), 135.5 (1C), 134.4 (1C), 129.2 (1C), 129.0 (2C), 128.2 (2C), 126.6 (1C), 66.6 (1C), 51.7 (1C), 47.4 (1C), 38.2 (1C), 22.5 (2C), 21.6 (1C). HRMS (ASAP): [M + H]^+^ theoretical for C_17_H_22_NSe^+^ *m*/*z* 320.0912, observed *m*/*z* 320.0914, Δppm = 0.6 ppm. HPLC Purity (7:3 Buffer:ACN, 220 nm): 98.6%, RT: 5.64 min.

### Induced Fit Docking

In silico docking was performed on the 1,2-diarylethylamine bioisostere series using the Induced Fit Docking (IFD) module of the Schrödinger 13.6/14.3 (release 2023-2/2025-1) Maestro modeling suite. The following PDB structures were utilized: NMDAR: 7SAC, DAT: 8Y2G, NET: 8Z1L. Protein structures were prepared using the Protein Preparation Wizard, with standard settings at pH 7.4 ± 2.0. Prior to protein preparation, the 7SAC protein structure was truncated by deleting residues over 30 Å beyond the PCP-binding site. All other receptors were prepared without modification. Ligands were prepared using LigPrep with standard settings (pH = 7.4 ± 2.0). The structures of the protonated amine species were carried forward for docking. Experimental ligands from each PDB structure were also included in the experiments to serve as a control. (+)-MK-801 was also used as a control in 7SAC. Schrodinger IFD was then employed with extended sampling. Given the symmetry of the PCP-binding site and lack of strong ionic interactions, a constraint was used for NMDAR (7SAC) such that the ligand cationic amine makes a NH^+^ H bond interaction with GluN2B Asn615. For analysis, poses were sorted by clustering with respect to the volume overlap of the ligand poses using the Clustering Based on Volume Overlap Panel with standard settings and selected based on making key contacts (e.g., ionic interactions involving the ammonium group, aryl–aryl interactions, etc.) similar to experimental ligands. Binding poses for figures were prepared in Maestro (Schrödinger 13.6). The Glide’s ptype.def file had to be modified to allow for incorporation of selenium atoms in Glide docking as outlined in the Schrödinger knowledge base.^117^

### In Silico Predictors

Molecular volume (Å^3^) and topological polar surface area (tPSA) parameters were calculated for aryl rings and the 1,2-diarylethylamine bioisostere series using Molinspiration Cheminformatics free web services. For the 1,2-diarylethylamine series in silico predictors were calculated on both the unprotonated and protonated forms. Given the p*K*a of the aliphatic amine present (predicted p*K*a range: 8.1–9.14, see SI Table S9), the protonated ammonium species is the predominant form at physiological pH and is believed to be the form that binds to the pharmacological targets. Stockholder (Hirshfeld) charges were calculated using the Schrödinger suite software (release 2025–2). Structures of the (S)-enantiomer were processed using LigPrep and geometry optimizations were performed using density functional theory (DFT) in the Jaguar module of the Schrödinger Suite, employing the B3LYP-D3 functional with the 6–31G basis set. To be thorough, stockholder charges were computed on both the ionized and un-ionized species as well as the corresponding aromatic rings. For the electrostatic surface potentials, the ligand structures were processed using the LigPrep tool in the Schrödinger suite (release 2025–2). Geometry optimization of the un-ionized ligand structures was performed using density functional theory (DFT) in the Jaguar tool with the B3LYP-D3 functional and the 6–31G** basis set. The un-ionized structures were selected to display electrostatic potential surfaces (ESPs), as it was found that the protonated FuPP molecule exhibited a unique energy minimized conformational state, that occluded visibility of the furan ring ESP. The protonation should not dramatically impact the ESP of the bioisostere ring portions of interest. Once computed the electrostatic surface was mapped from volume onto an isodensity surface derived from electron density at 0.001 e/bohr. The surface was visualized at 30% transparency and displayed in red-white-blue color scheme at the range of −15 to 15 kcal/mol.

## Supplementary Material





## Notes

Target compound SMILES (CSV)
Supplementary figures and tables including molecular numbering schemes; induced-fit docking poses at NMDAR, DAT, and NET; HPLC traces; ^1^H/^13^C NMR spectra and chemical shift assignment tables; molecular descriptors (volume, tPSA, electronegativity); stockholder charge analyses for ionized and un-ionized species; predicted p*K*_a_ values; SERT binding inhibition and concentration–response data; LC-MS/MS calibration curves and compound-specific parameters; plasma and brain homogenate quantification of SePP; individual mouse PK data at 10 and 120 min; and behavioral beam test scoring (PDF)

## Abbreviations

AGS: audiogenic seizures
DAT: dopamine reuptake transporter
FXS: fragile X syndrome
i.p.: intraperitoneal
MDD: major depressive disorder
NET: norepinephrine reuptake transporter
NMDAR: *N*-methyl-d-aspartate receptor
PCP: phencyclidine
PTSD: post-traumatic stress disorder
RPM: revolutions per minute
SERT: serotonin reuptake transporter
tPSA: topological polar surface area
TRD: treatment resistant depression
