# Supplementary material for: Bioisostere-Driven Discovery of SePP: A Selenium-Containing Polypharmacological Agent Relevant to Fragile X Syndrome
Source: J Med Chem. 2026 Feb 10;69(4):4020–36. doi: 10.1021/acs.jmedchem.5c02700 (PMC12951560; doi:10.1021/acs.jmedchem.5c02700)
Supplement: Supplementary file 2 [file jm5c02700_si_002.pdf]

Supporting Information

for

Bioisostere-Driven Discovery of SePP: A Selenium-Containing Polypharmacological Agent Relevant to Fragile X Syndrome

Jason Wallach[1*], Sean Cameron[1], Michael Dybek[1], Branden Stanley[2], Christopher Orme[1], Nour Riad[1], Pierce Kavanagh[3], Simon Brandt[4], Adam Knapp[1], James Gamrat[1], Rebekah Jauhola-Straight[1], Adeboye Adejare[1,2], Alexander J. Rogier[5], Richa Tyagi[5], Clinton E. Canal[5]

[1] - Philadelphia College of Pharmacy. Saint Joseph's University. Department of Pharmaceutical Sciences. 600 South 43rd St. Philadelphia PA 19104, United States
[2] - Philadelphia College of Pharmacy. Saint Joseph's University. Department of Chemistry. 600 South 43rd St. Philadelphia PA 19104, United States
[3] Discipline of Pharmacology and Therapeutics, School of Medicine, Trinity College Dublin, Dublin 2, Ireland
[4] The Alexander Shulgin Research Institute, 1483 Shulgin Road, Lafayette, CA 94549, United States
[5] College of Pharmacy, Department of Pharmaceutical Sciences, Mercer University, 3001 Mercer University Drive, Atlanta, Georgia 30341, United States

Corresponding Author

Jason Wallach, Email: jwallach@sju.edu

Supporting Information Table of Contents

Supporting Information Figures

Figure S1. Numbering Scheme for 1,2-Diarylethylamine Bioisostere Series - S3

Figure S2A-O. NMDAR Induced Fit Docking Poses - S3-S17

Figure S3A-M. DAT Induced Fit Docking Poses - S18-S30

Figure S4A-M. NET Induced Fit Docking Poses – S31-S43

Figure S5A-E. HPLC Traces – S44-S45

Figure S6A-J ^{1}H and ^{13}C NMR Spectra – S45-50

Figure S7. SERT Citalopram, DPP and TPP Concentration Response Curves – S50

Figure S8. SePP Plasma Calibration Curve – S51

Figure S9. SePP Mouse Brain Homogenate Calibration Curve – S52

Supporting Information Tables

Table S1. ^{1}H NMR Chemical Shift Assignment Table - S53-54

Table S2. ^{13}C NMR Chemical Shift Assignment Table – S55

Table S3. Molecular Volume and Topological Polar Surface Area (tPSA) Values – S56

Table S4. 1,2-Diarylethylamine Bioisostere Series Stockholder Charges for NH+ Compounds – S56-58

Table S5.1,2-Diarylethylamine Bioisostere Series Stockholder Charges for Unionized Compounds – S58-59

Table S6. Aromatic Ring Stockholder Charges – S60

Table S7. Predicted (Jaguar) p*K*a for 1,2-Diarylethylamine Bioisosteres – S60

Table S8. Pauling Scale Electronegativity values for C, O, S, and Se – S61

Table S9. % Inhibition of [^{3}H]-Citalopram SERT Binding 10 μM Screening – S61

Table S10. Beam Test Scoring – S61

Table S11. Concentration of SePP versus SePP/IS peak area ratio in Plasma – S62

Table S12. Plasma concentrations of SePP in individual mice at the 10-min – S63

Table S13. Plasma concentrations of SePP in individual mice at the 120-min – S63

Table S14. Concentration of SePP versus SePP/IS peak area ratio in Mouse Brain Homogenate – S64

Table S15. LC-MS/MS compound dependent parameters – S64

References – S64

Supporting Information Figures

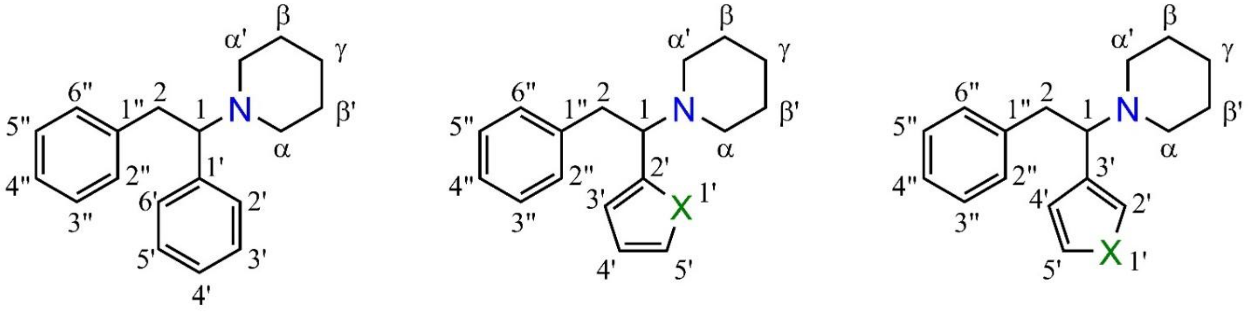

Figure S1. Compound Numbering Scheme for 1,2-Diarylethylamine Bioisostere Series.

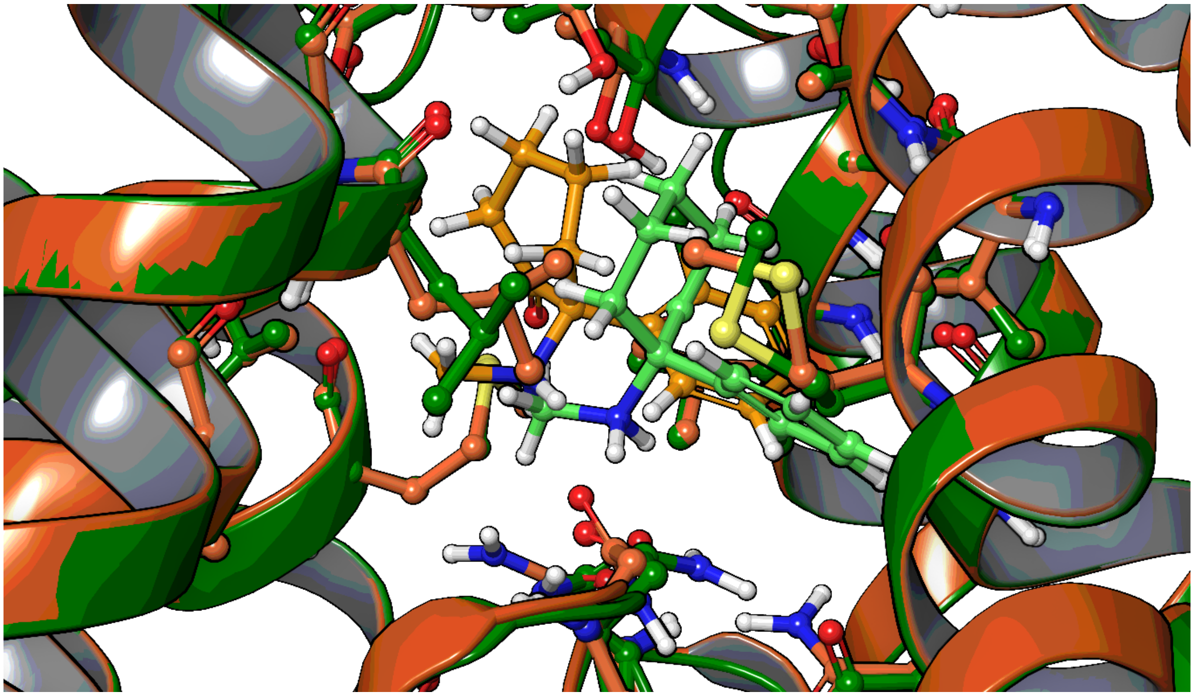

Figure S2A. Induced Fit Docking Pose For (S)-ketamine (green) at NR1/NR2B NMDAR (PDB: 7SAC) Overlaid with its Experimental Structure (orange), RMSD = 3.056 Å.

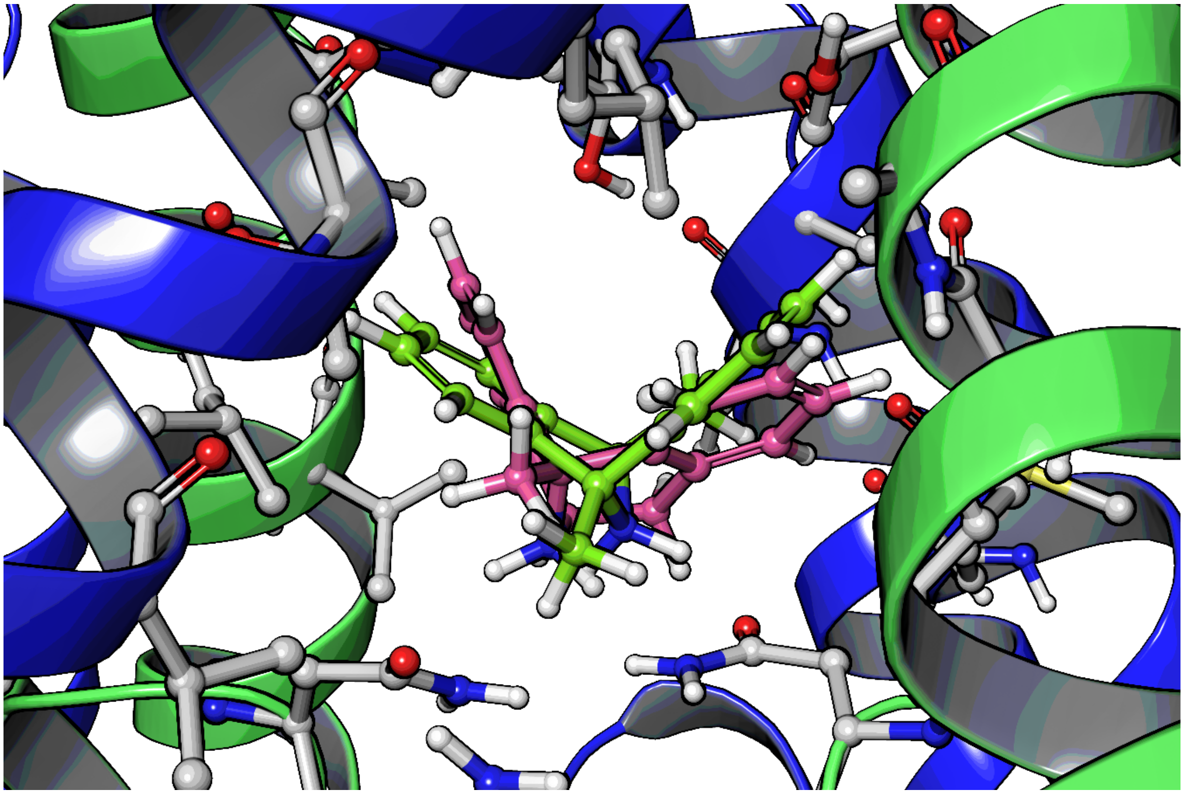

Figure S2B. Induced Fit Docking Pose For (+)-MK-801 (pink) Overlaid with (+)-MK-801 (green) Bound at Experimental NR1/NR2A/NR2B NMDAR Structure (PDB: 5UOW) at NR1/NR2B NMDAR (PDB: 7SAC), RMSD = 1.870.

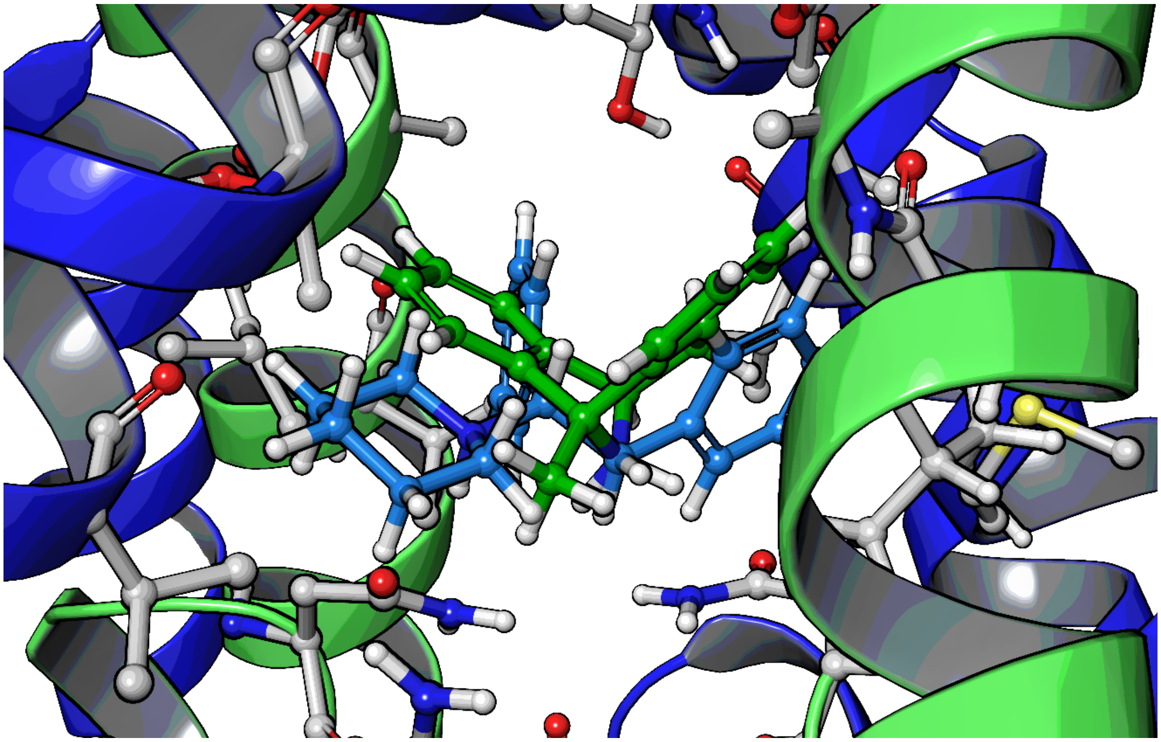

Figure S2C. Induced Fit Docking Pose For (S)-Diphenidine (blue) at NR1/NR2B NMDAR (PDB: 7SAC) Overlaid with (+)-MK-801 (green) Bound at Experimental NR1/NR2A/NR2B NMDAR Structure (PDB: 5UOW).

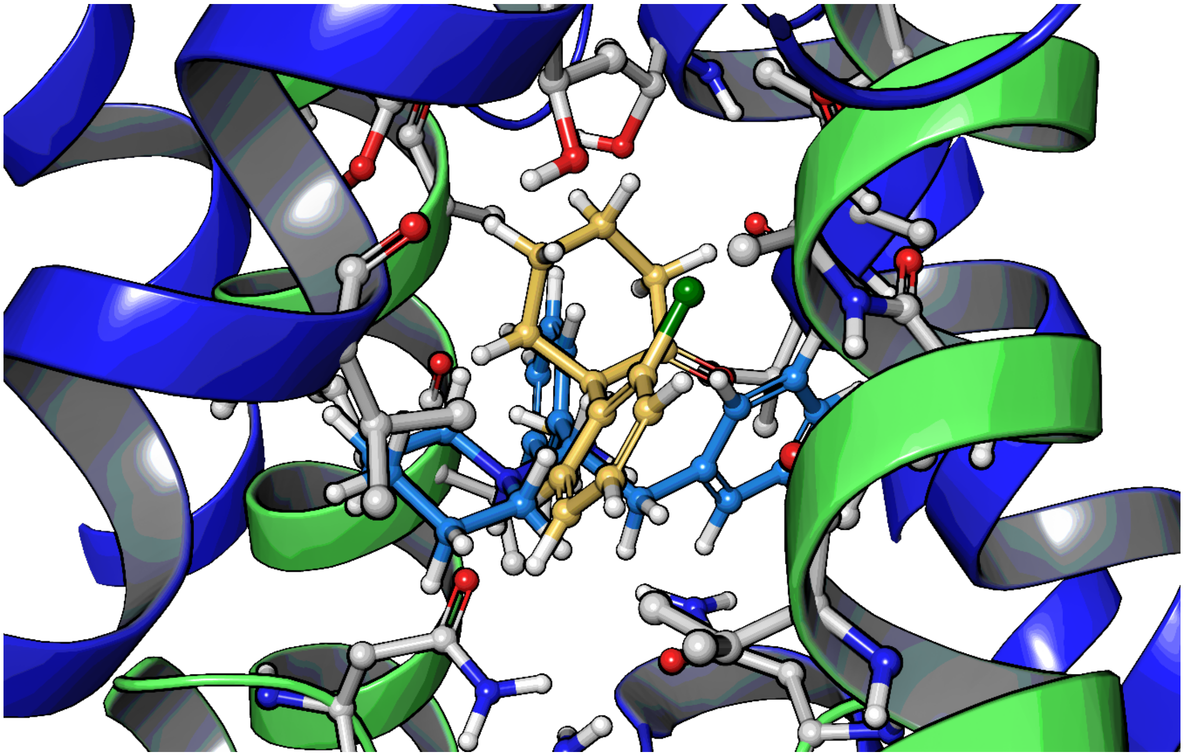

Figure S2D. Induced Fit Docking Pose For (S)-Diphenidine (blue) Overlaid with (S)-ketamine (gold) Bound at experimental NR1/NR2B NMDAR structure (PDB: 7SAC).

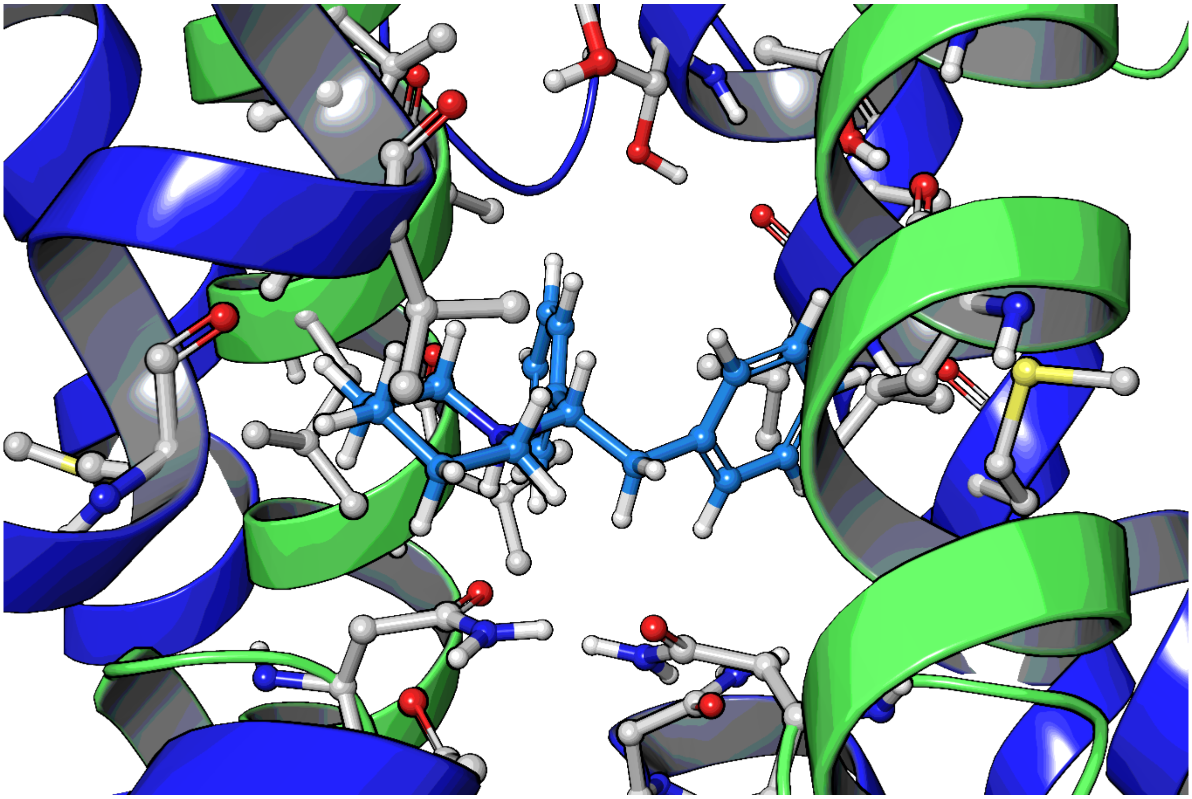

Figure S2E. Induced Fit Docking Pose For (S)-Diphenidine (blue) at NR1/NR2B NMDAR (PDB: 7SAC).

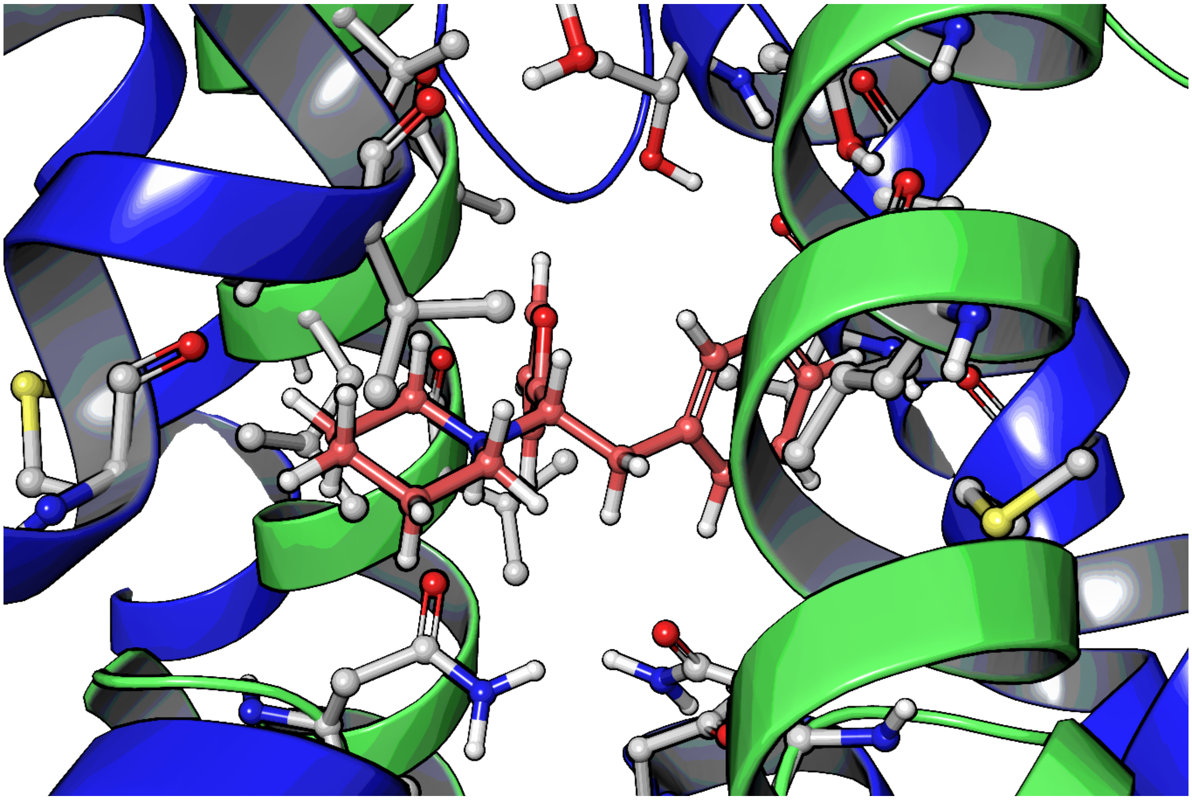

Figure S2F. Induced Fit Docking Pose For (S)-FuPP (red) at NR1/NR2B NMDAR (PDB: 7SAC).

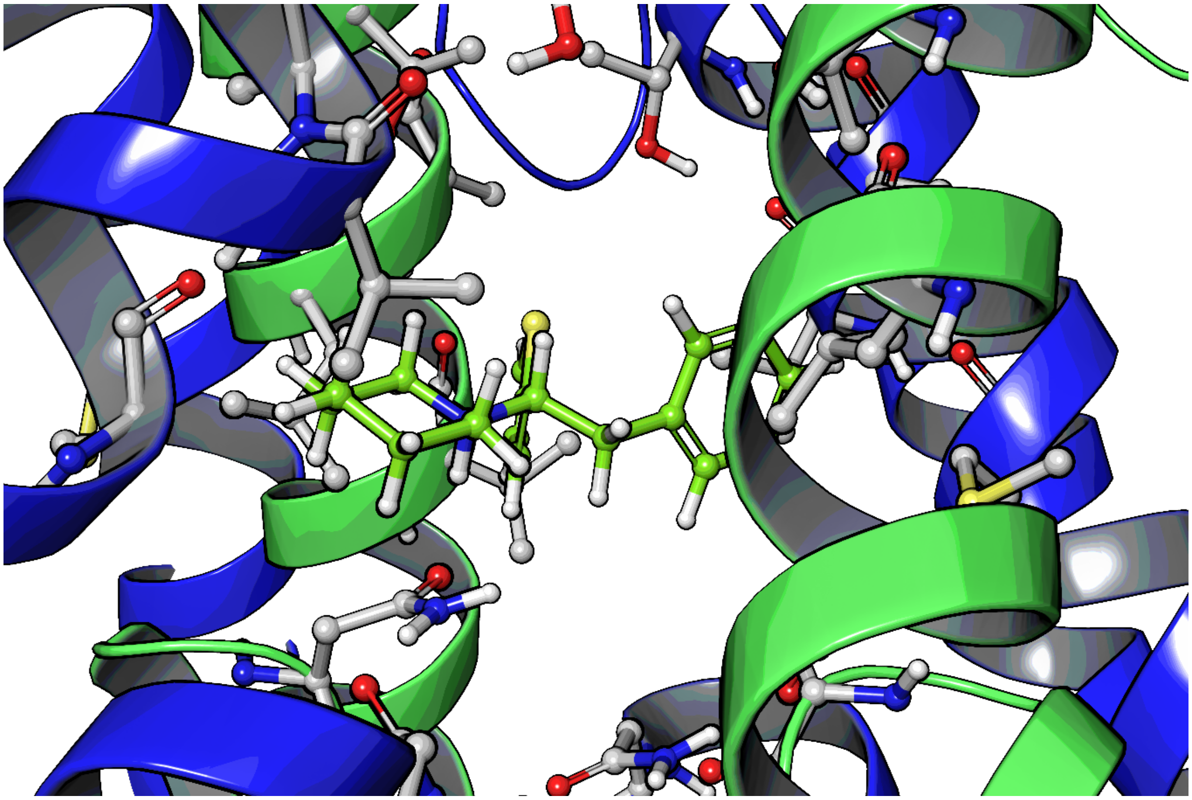

Figure S2G. Induced Fit Docking Pose For (S)-TPP (green) at NR1/NR2B NMDAR (PDB: 7SAC).

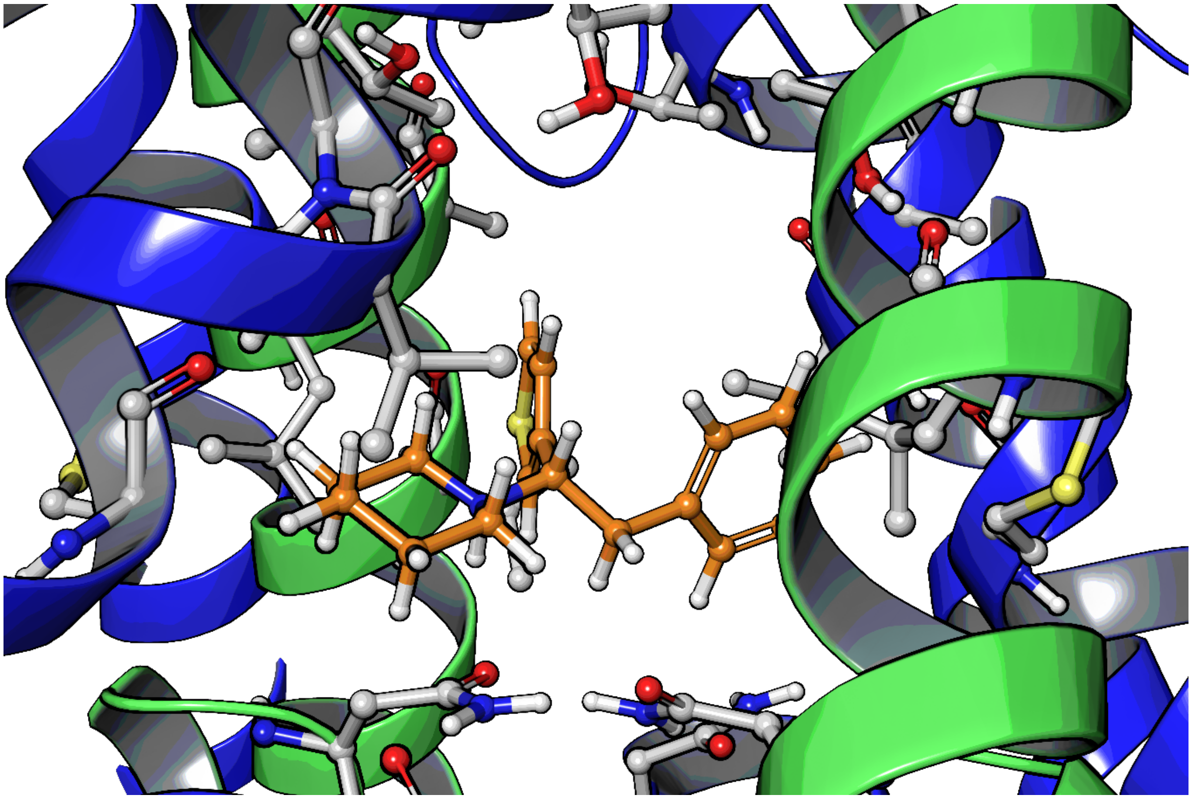

Figure S2H. Induced Fit Docking Pose For (S)-3-TPP (orange) at NR1/NR2B NMDAR (PDB: 7SAC).

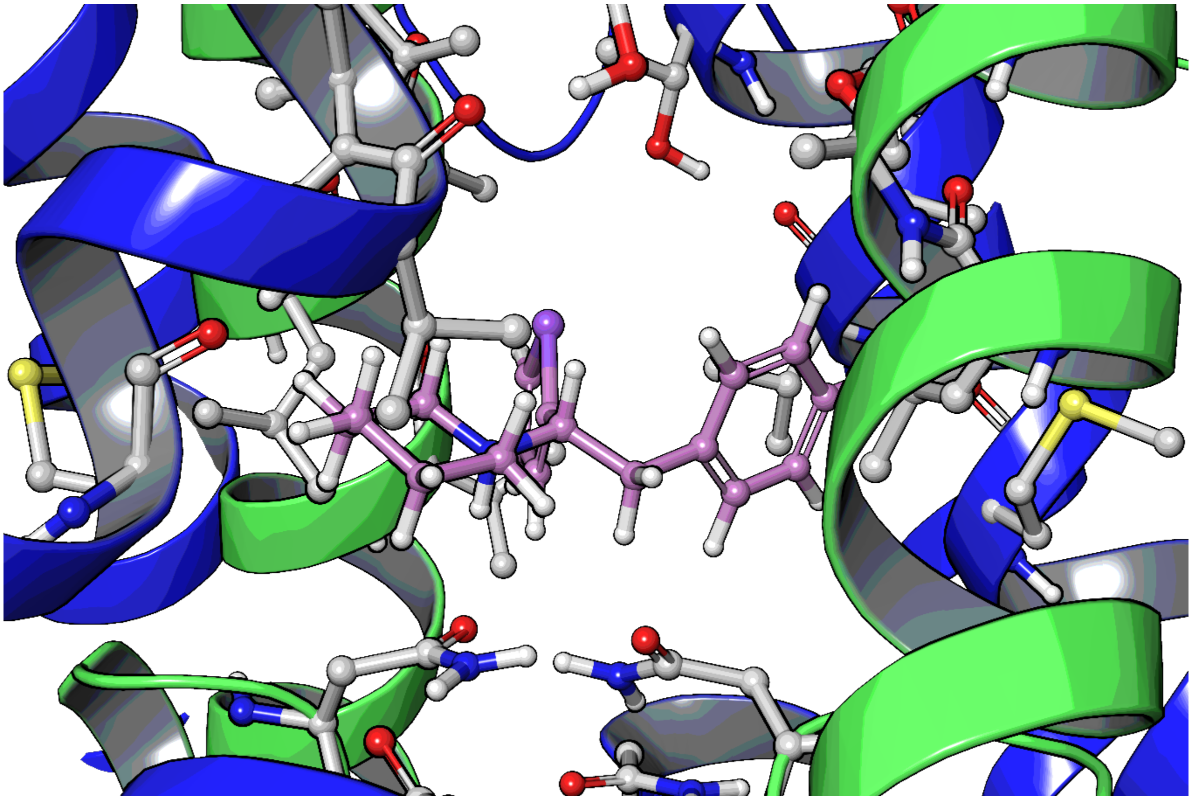

Figure S2I. Induced Fit Docking Pose For (S)-SePP (purple) at NR1/NR2B NMDAR (PDB: 7SAC).

.

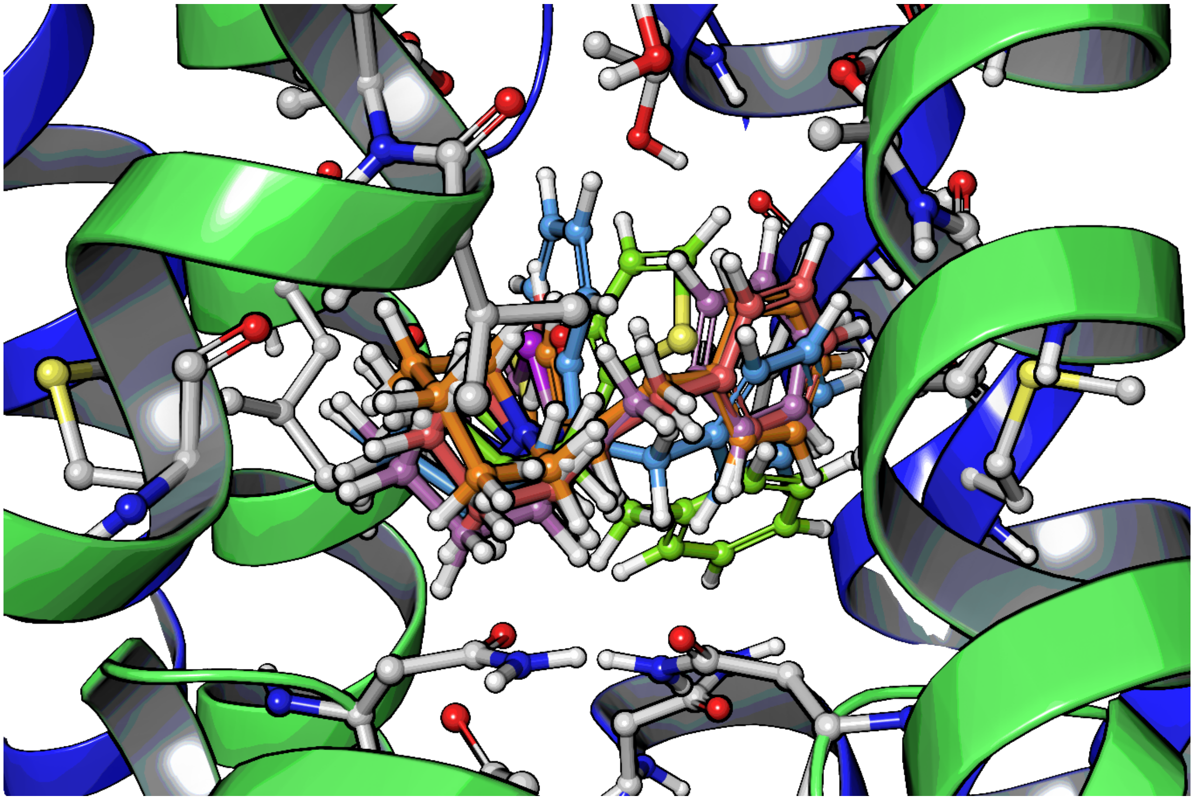

Figure S2J. Overlay of Induced Fit Docking (R)-Diarylethylamines Poses at NR1/NR2B NMDAR (PDB: 7SAC). (R)-Diphenidine (blue), (R)-FPP (red), (R)-TPP (green), (R)-3-TPP (orange) and (R)-SePP (purple).

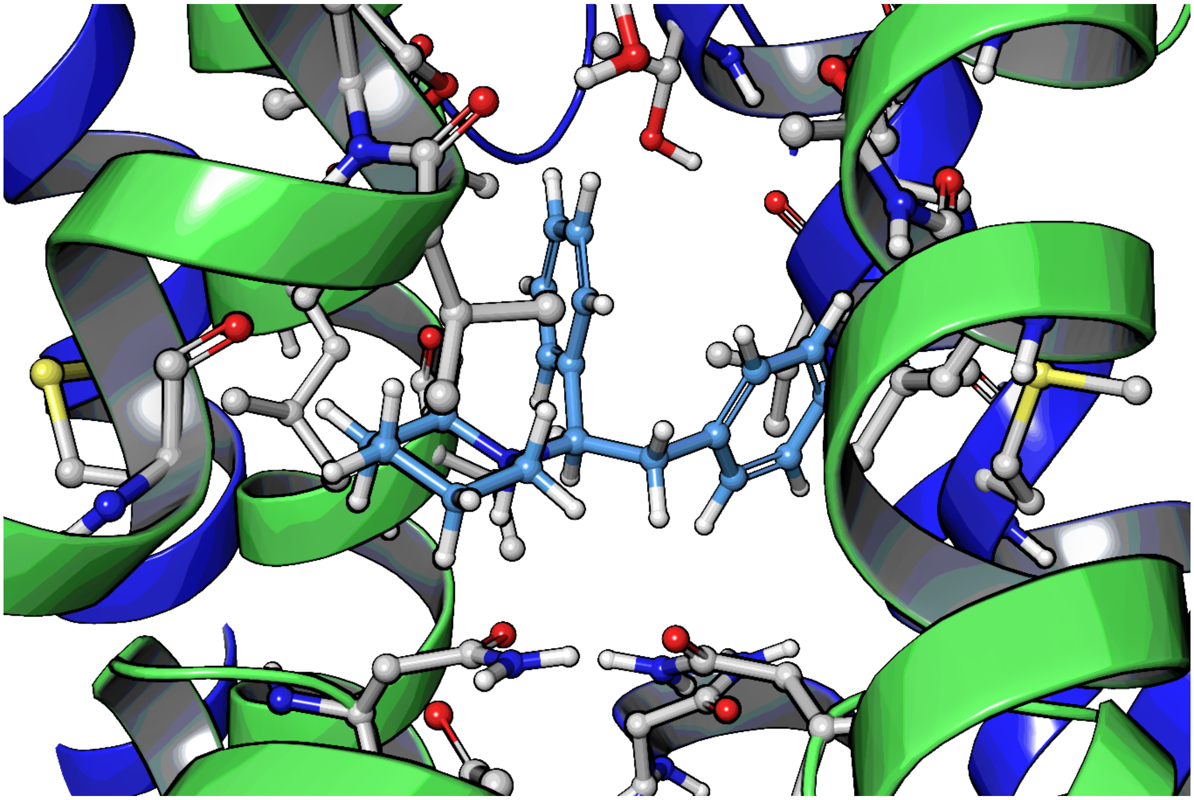

Figure S2K. Induced Fit Docking Pose For (R)-Diphenidine (blue) at NR1/NR2B NMDAR (PDB: 7SAC).

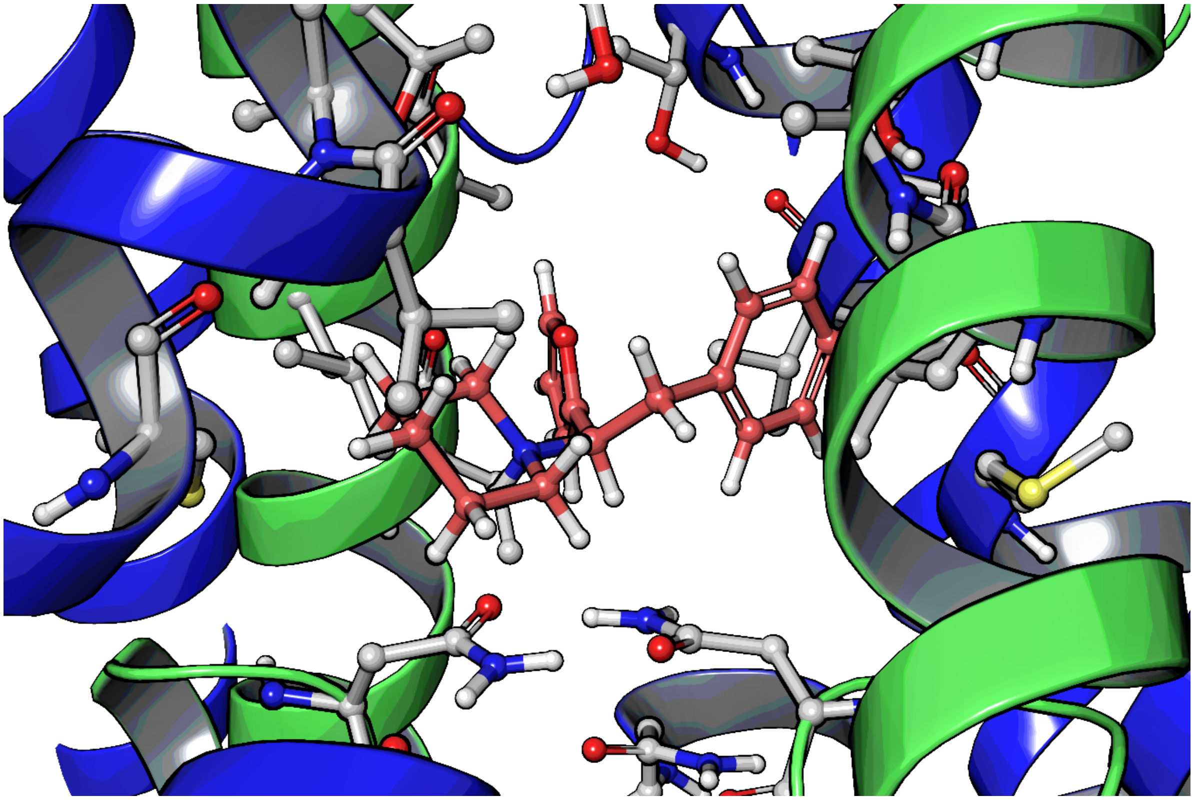

Figure S2L. Induced Fit Docking Pose For (R)-FuPP at NR1/NR2B NMDAR (PDB: 7SAC).

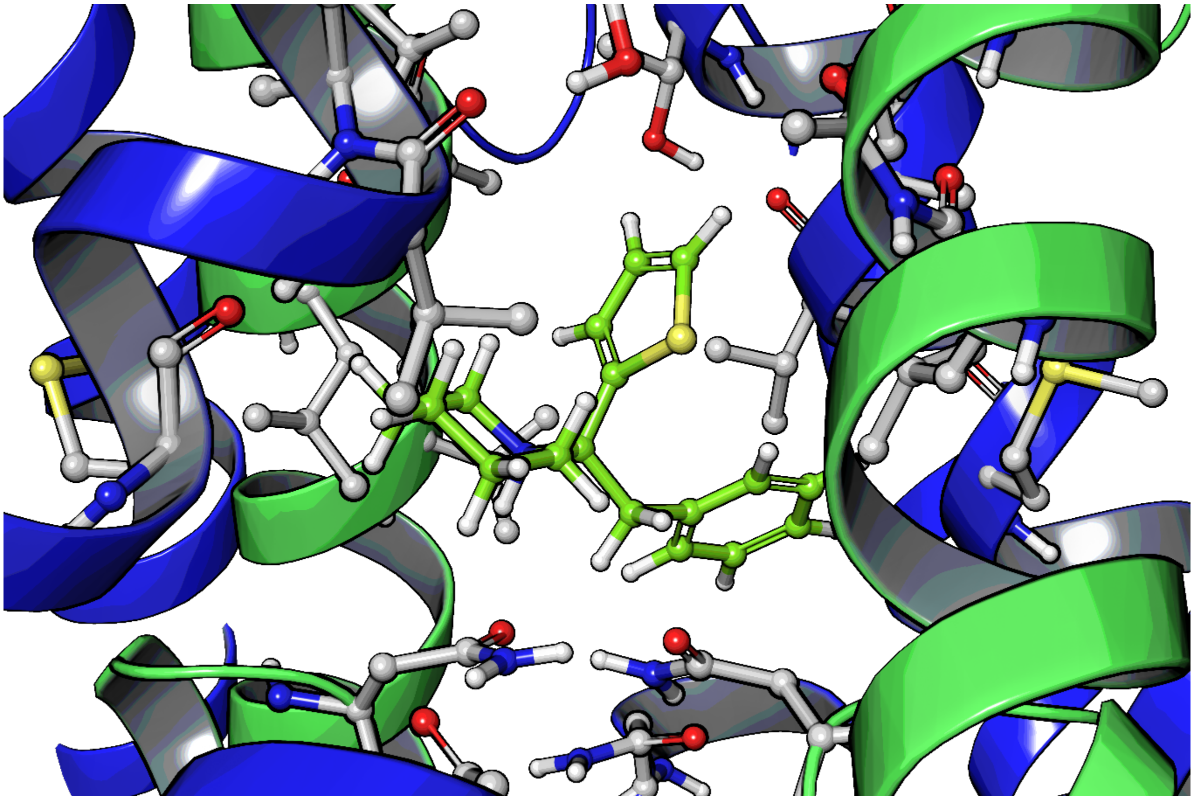

Figure S2M Induced Fit Docking Pose for (R)-TPP at NR1/NR2B NMDAR (PDB: 7SAC).

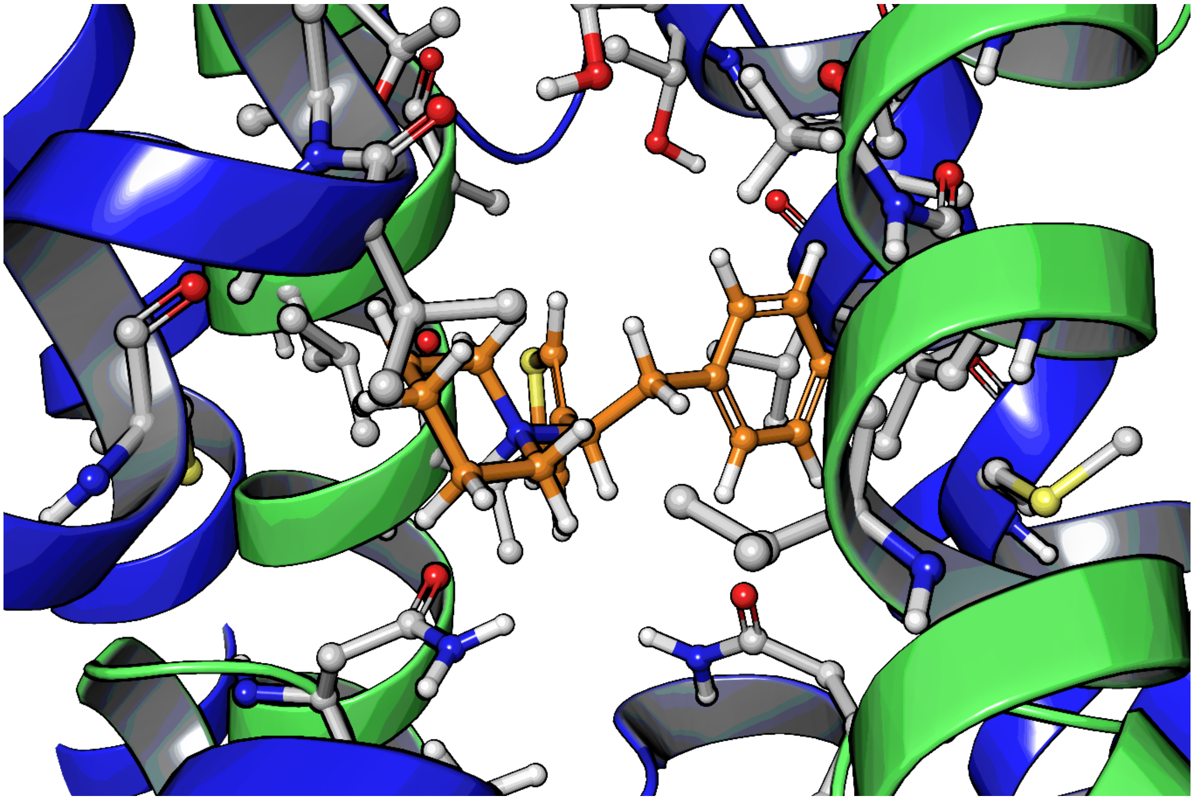

Figure S2N. Induced Fit Docking Pose for (R)-3-TPP at NR1/NR2B NMDAR (PDB: 7SAC).

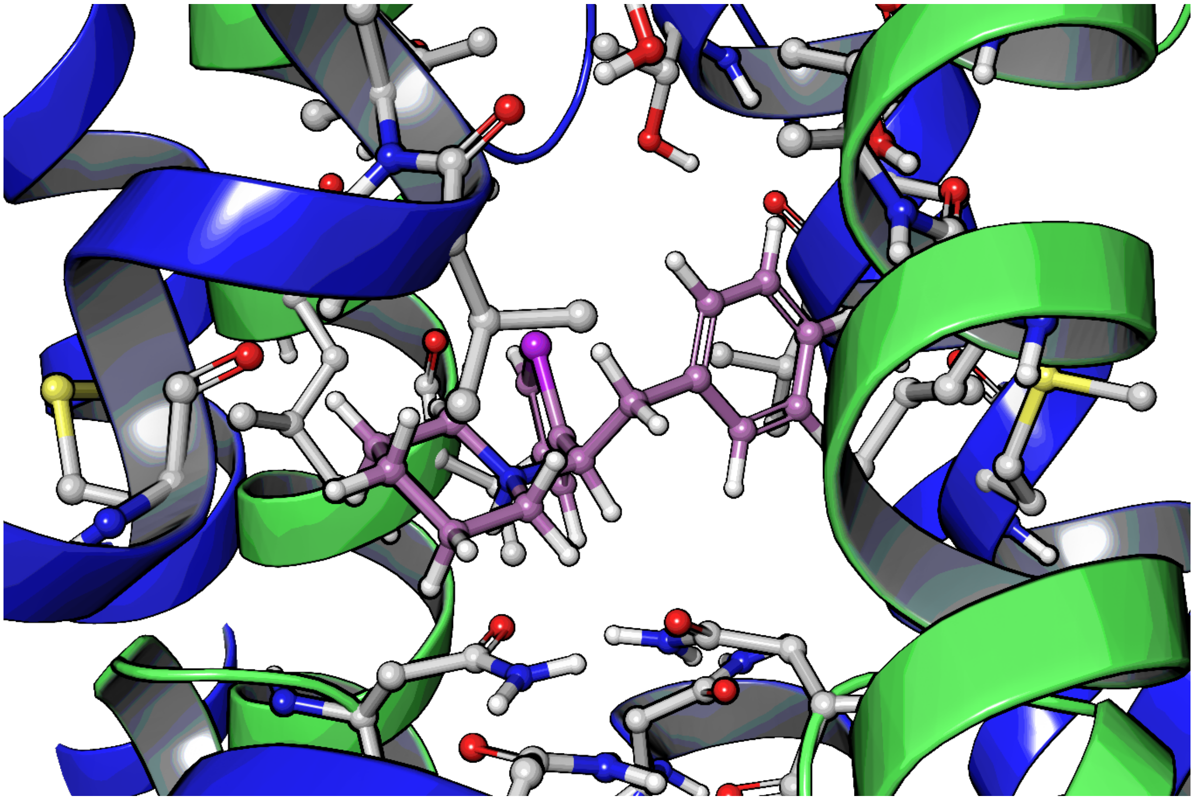

Figure S2O. Induced Fit Docking Pose for (R)-SePP at NR1/NR2B NMDAR (PDB: 7SAC).

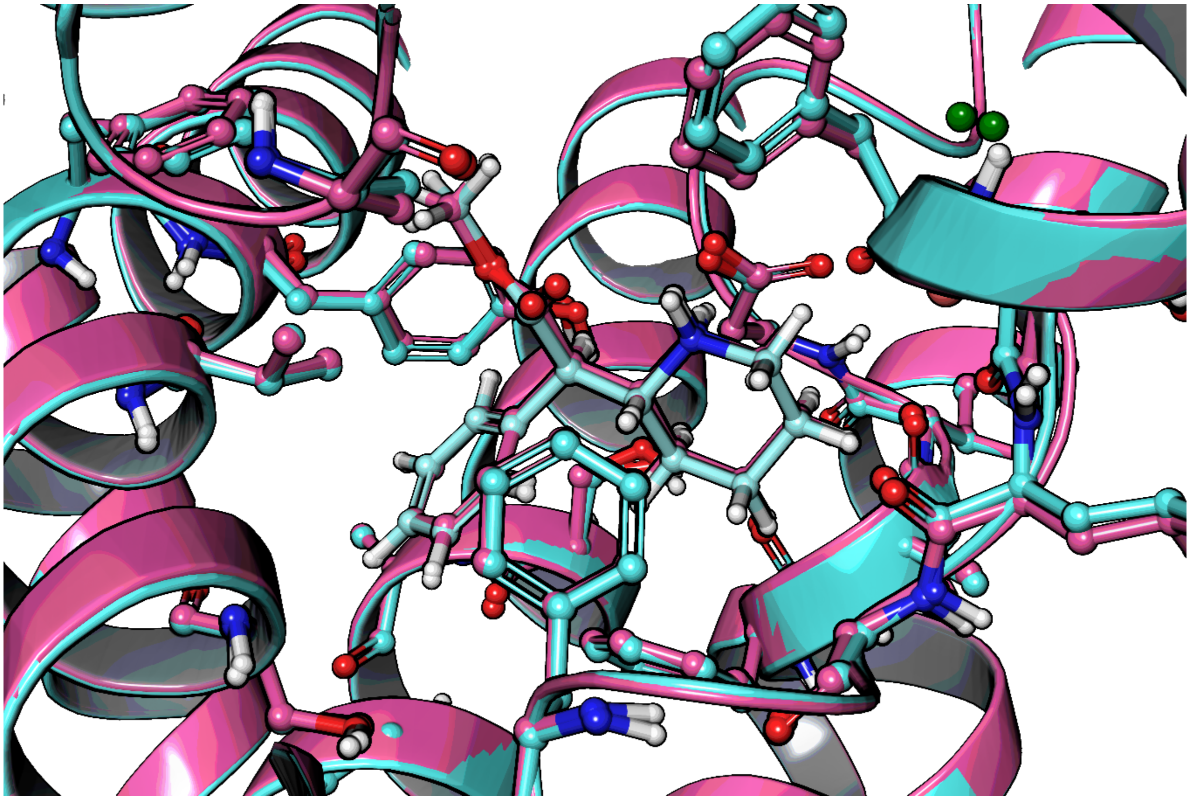

Figure S3A. Induced Fit Docking Pose of methylphenidate (pink) at the DAT (PDB: 8Y2G) Overlaid with its Experimental Structure, RMSD = 0.253 Å.

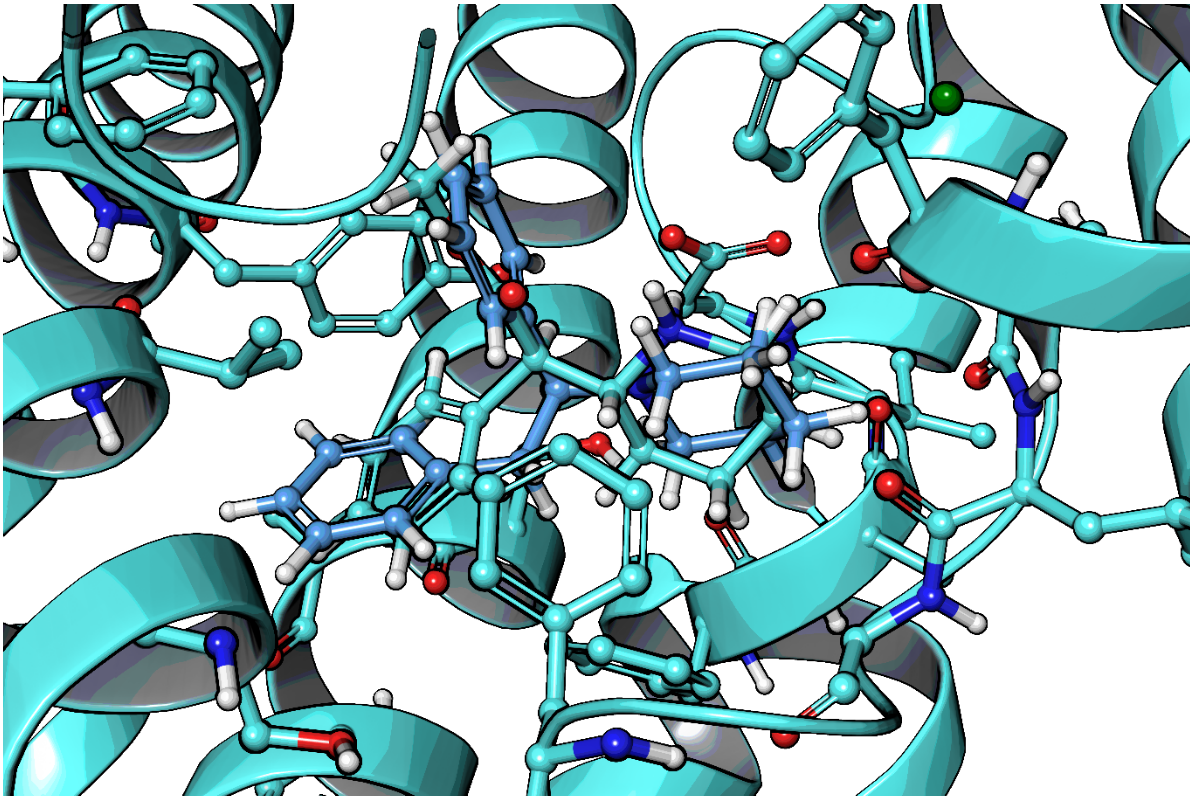

Figure S3B. (S)-Diphenidine IFD Pose (blue) Overlaid with the Experimental Methylphenidate DAT Structure (cyan) (PDB: 8Y2G).

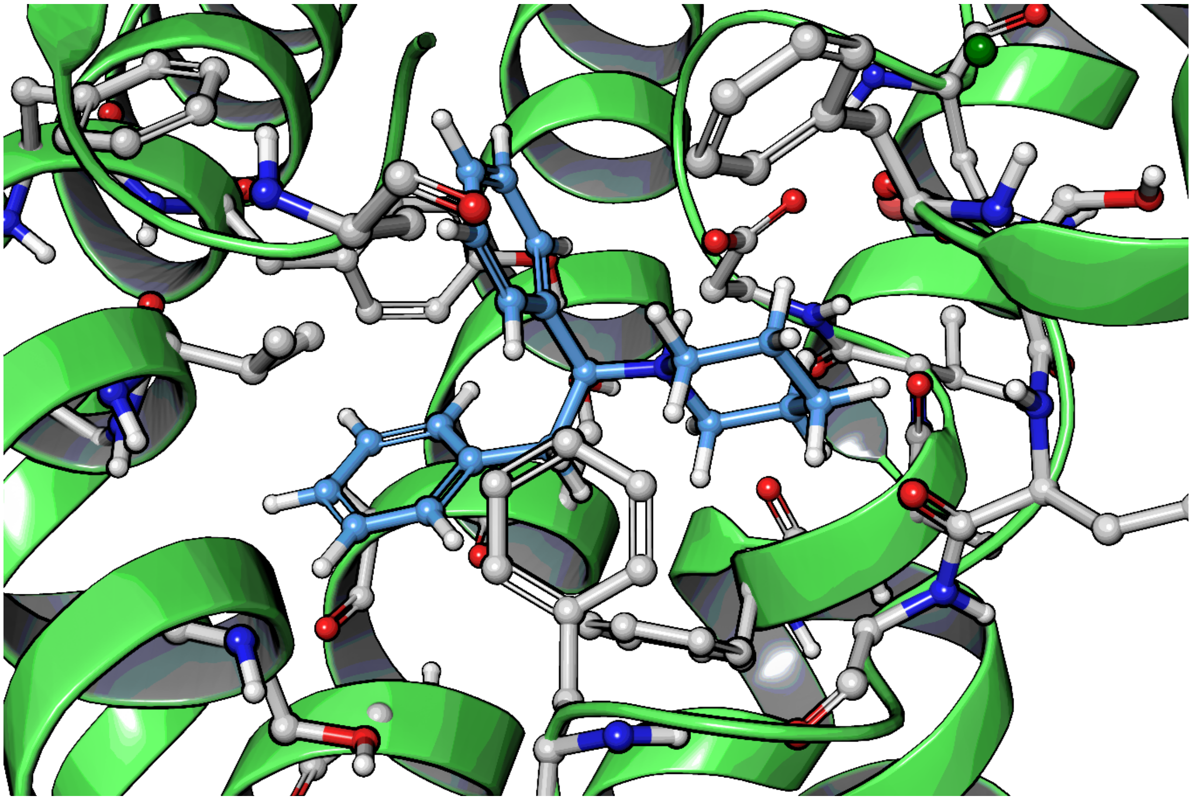

Figure S3C. Induced Fit Docking Pose for (S)-Diphenidine (blue) at the DAT (PDB: 8Y2G).

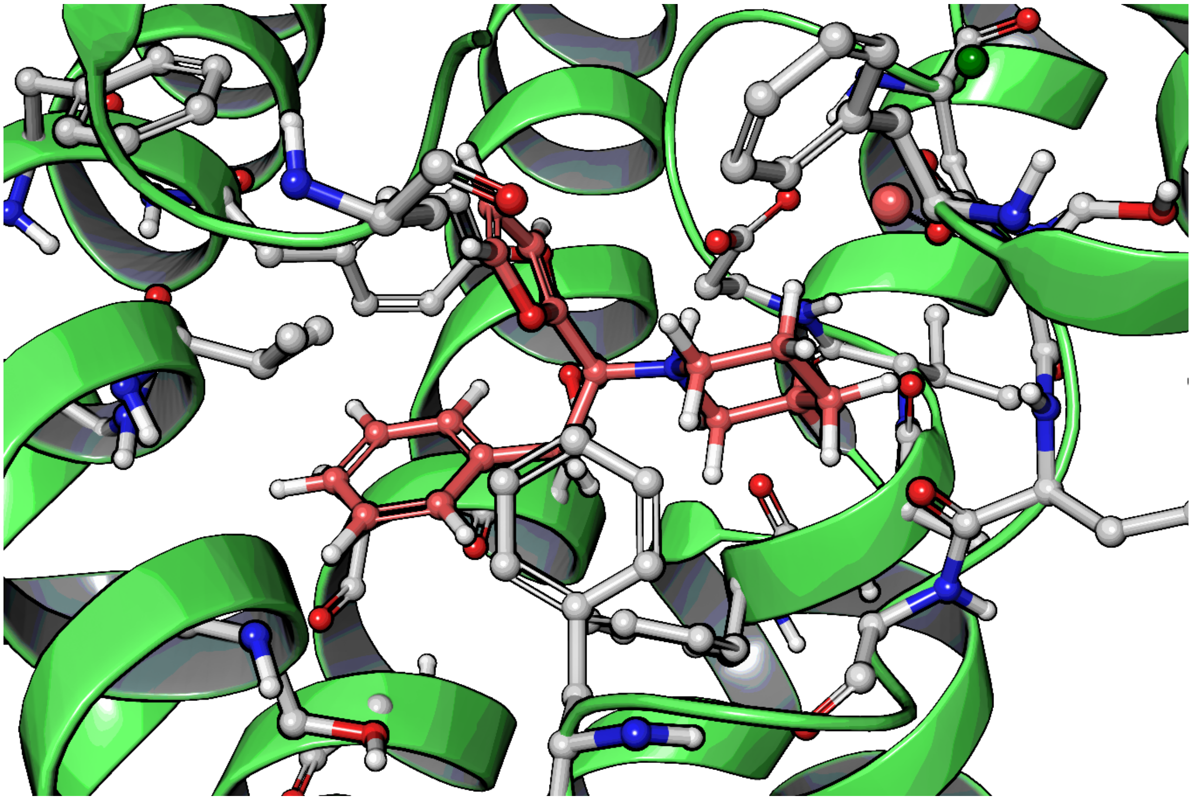

Figure S3D. Induced Fit Docking Pose for (S)-FuPP (blue) at the DAT (PDB: 8Y2G).

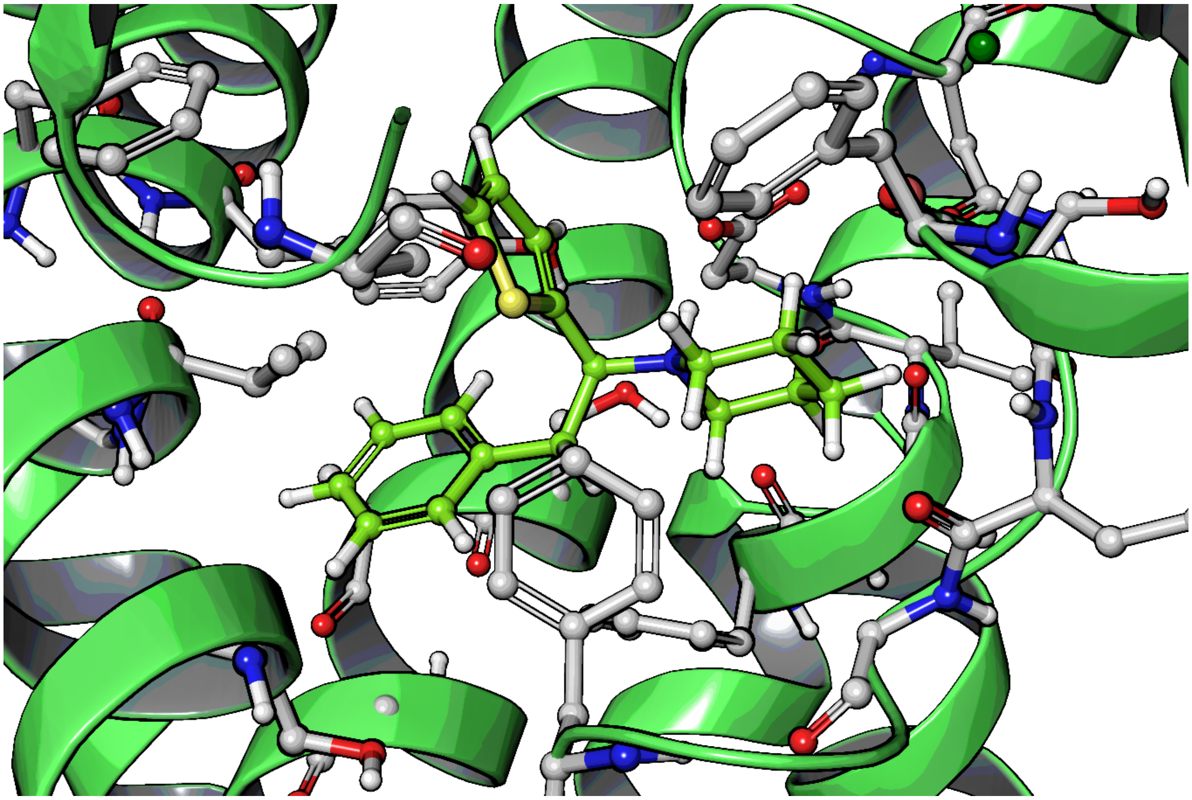

Figure S3E. Induced Fit Docking Pose for (S)-2-TPP (green) at the DAT (PDB: 8Y2G).

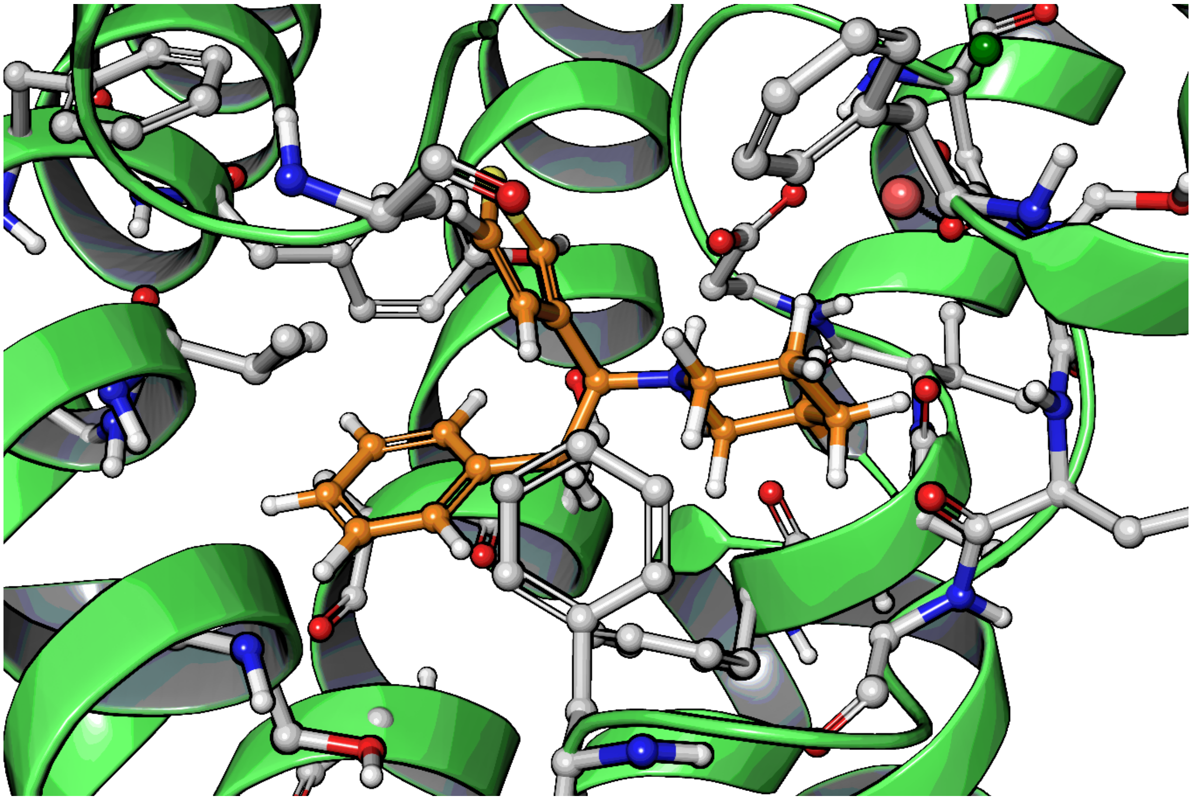

Figure S3F. Induced Fit Docking Pose for (S)-3-TPP (orange) at the DAT (PDB: 8Y2G).

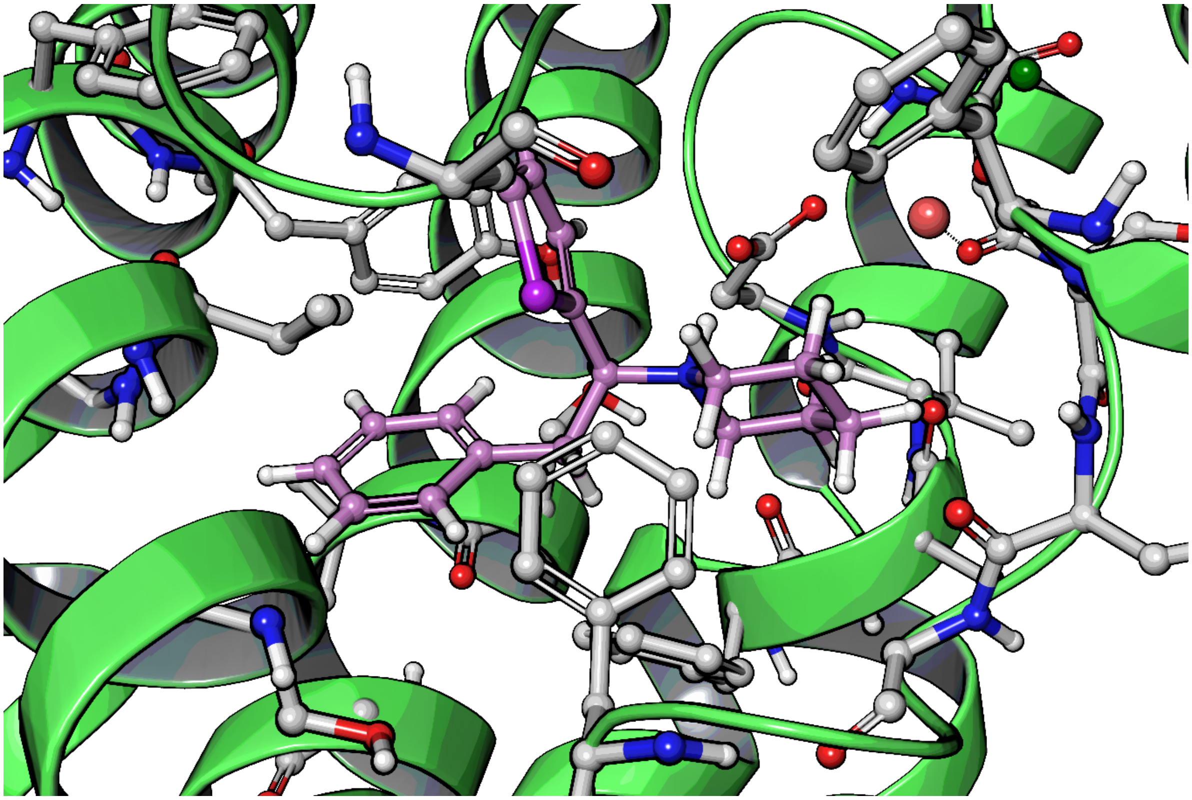

Figure S3G. Induced Fit Docking Pose for (S)-SePP (purple) at the DAT (PDB ID: 8Y2G).

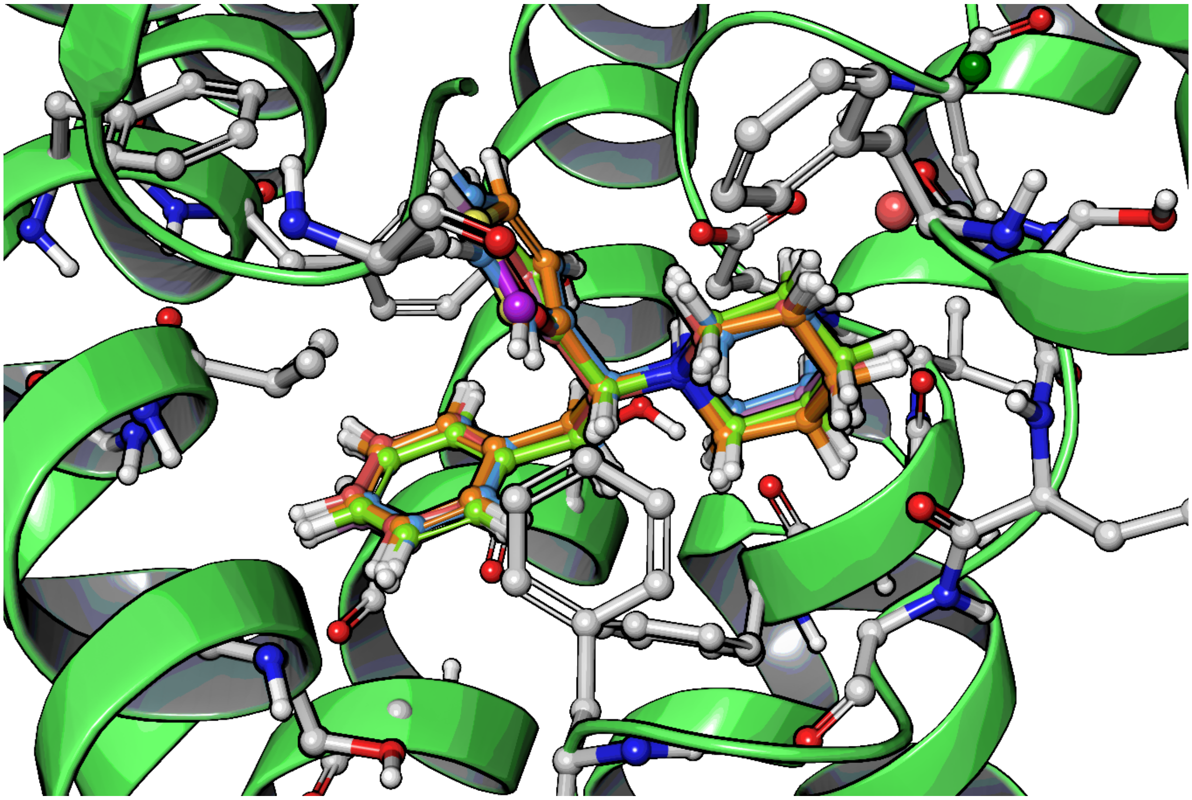

Figure S3H. Overlay of Induced Fit Docking (R)-Diarylethylamines Poses at the DAT (PDB: 8Y2G). (R)-Diphenidine (blue), (R)-FuPP (red), (R)-2-TPP (green), (R)-3-TPP (orange), (R)-SePP (purple).

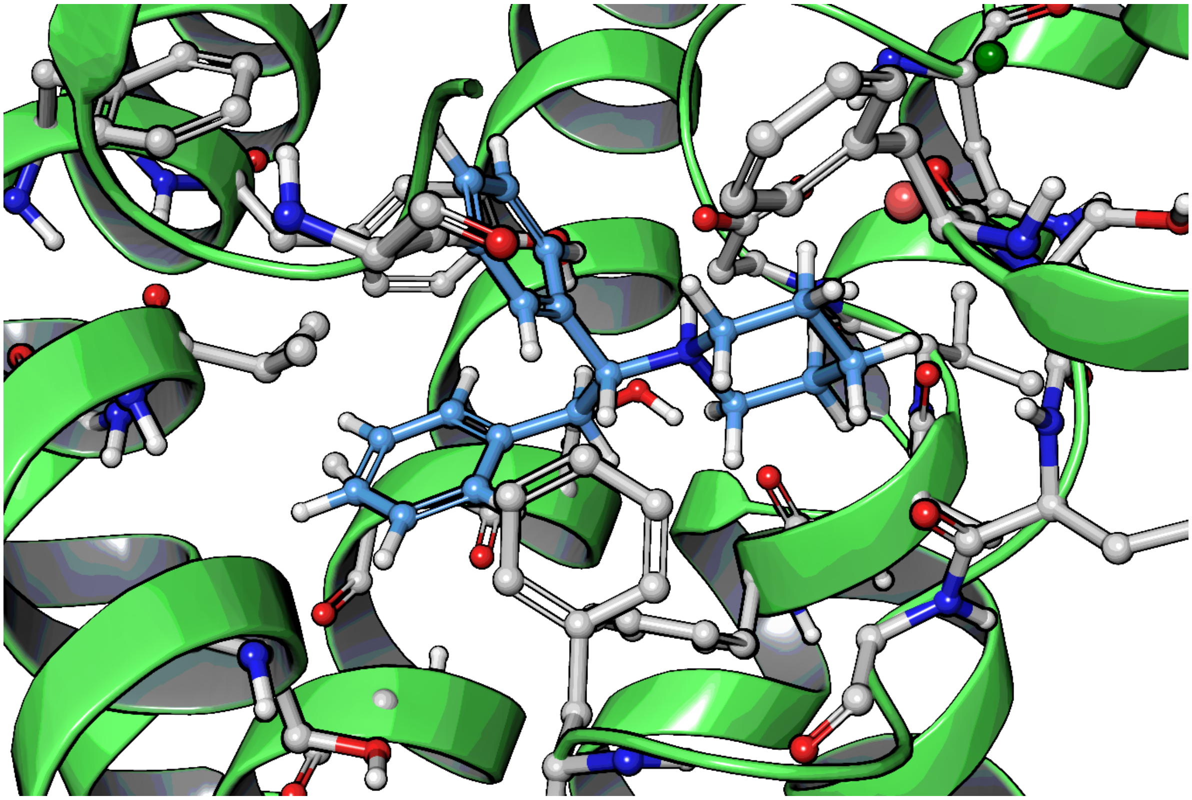

Figure S3I. Induced Fit Docking Pose for (R)-Diphenidine (blue) at the DAT (PDB: 8Y2G).

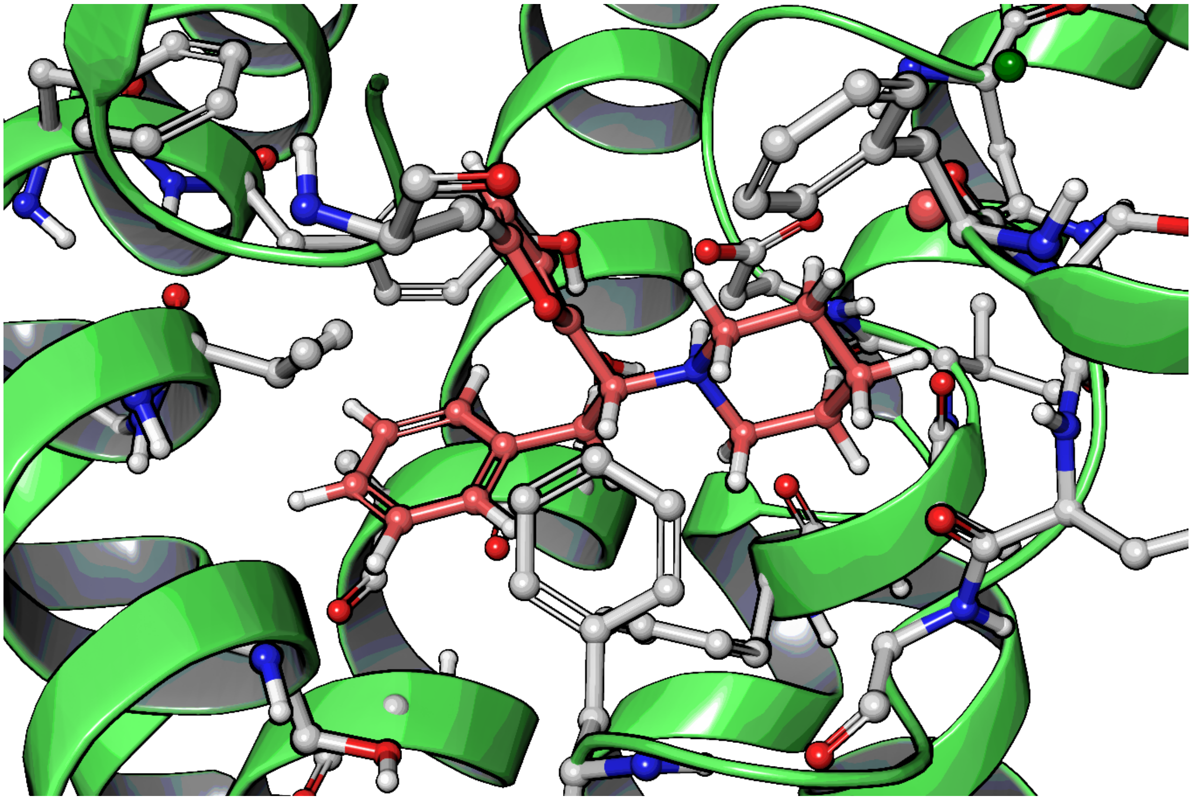

Figure S3J Induced Fit Docking Pose for (R)-FuPP (red) at the DAT (PDB: 8Y2G).

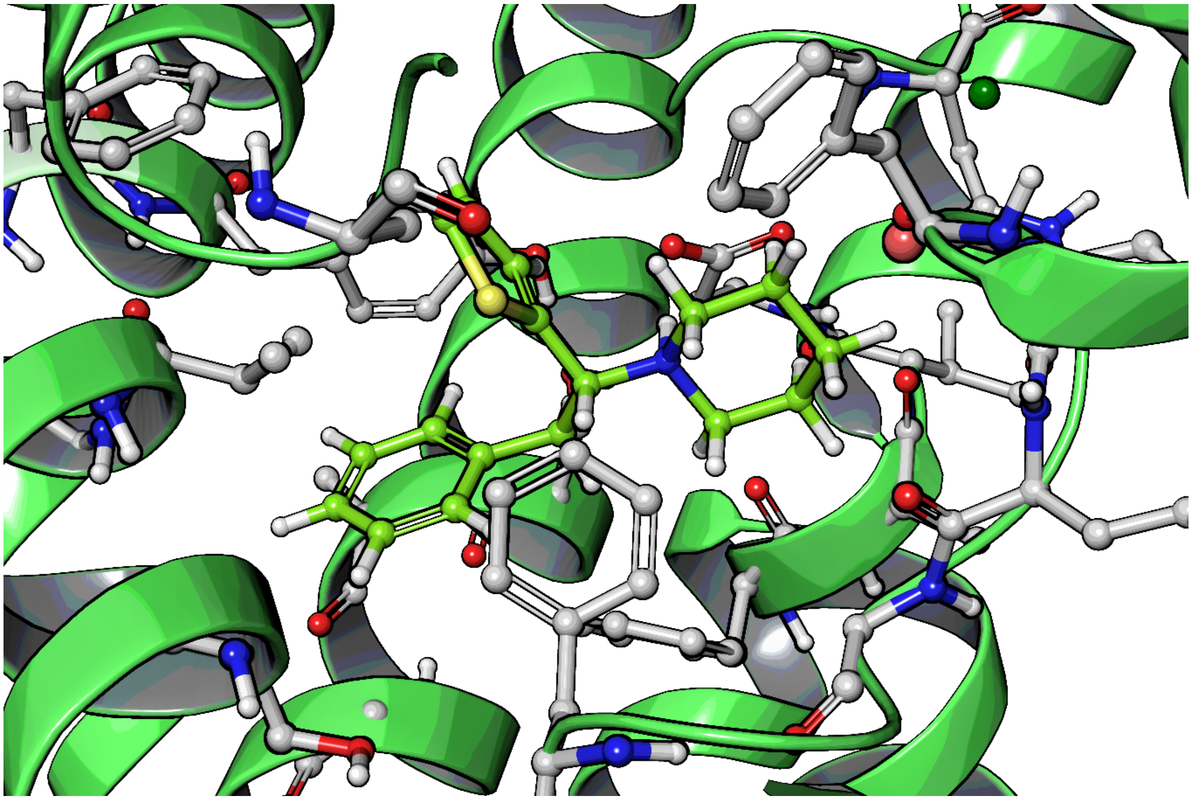

Figure S3K. Induced Fit Docking Pose for (R)-TPP (green) at the DAT (PDB: 8Y2G).

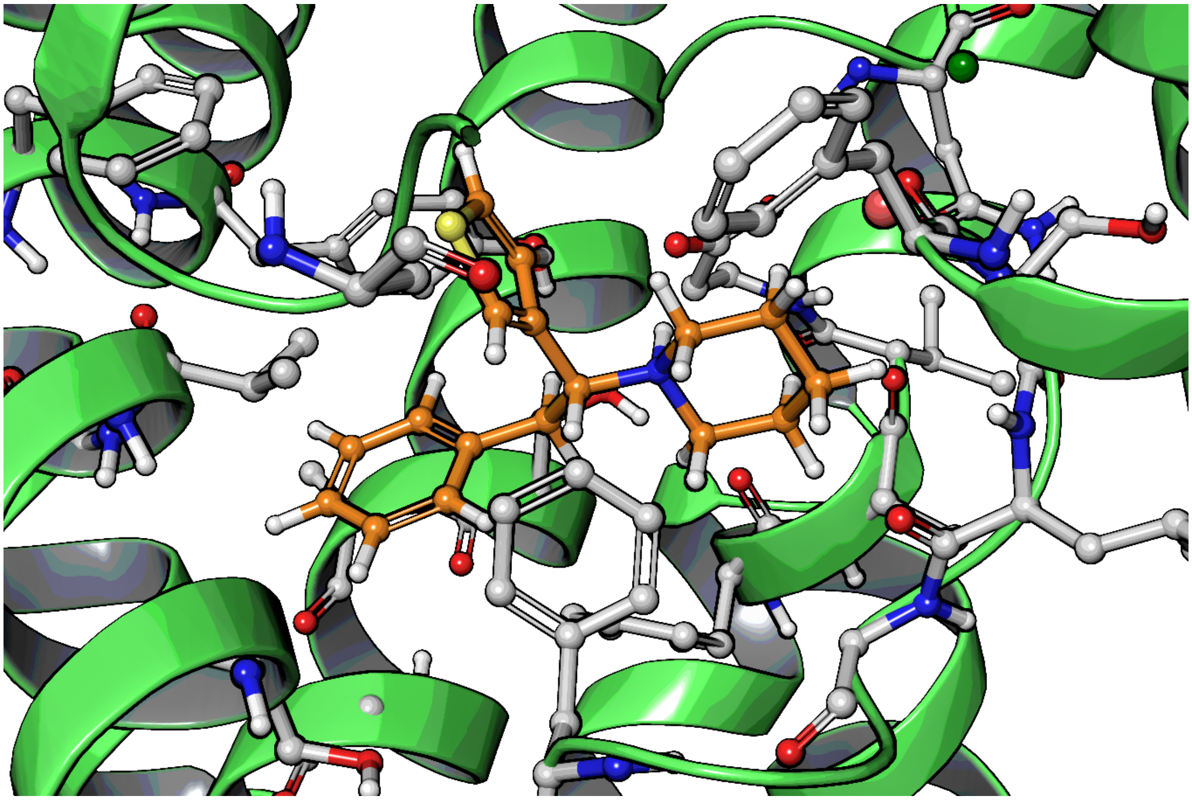

Figure S3L. Induced Fit Docking Pose for (R)-3-TPP (orange) at the DAT (PDB: 8Y2G).

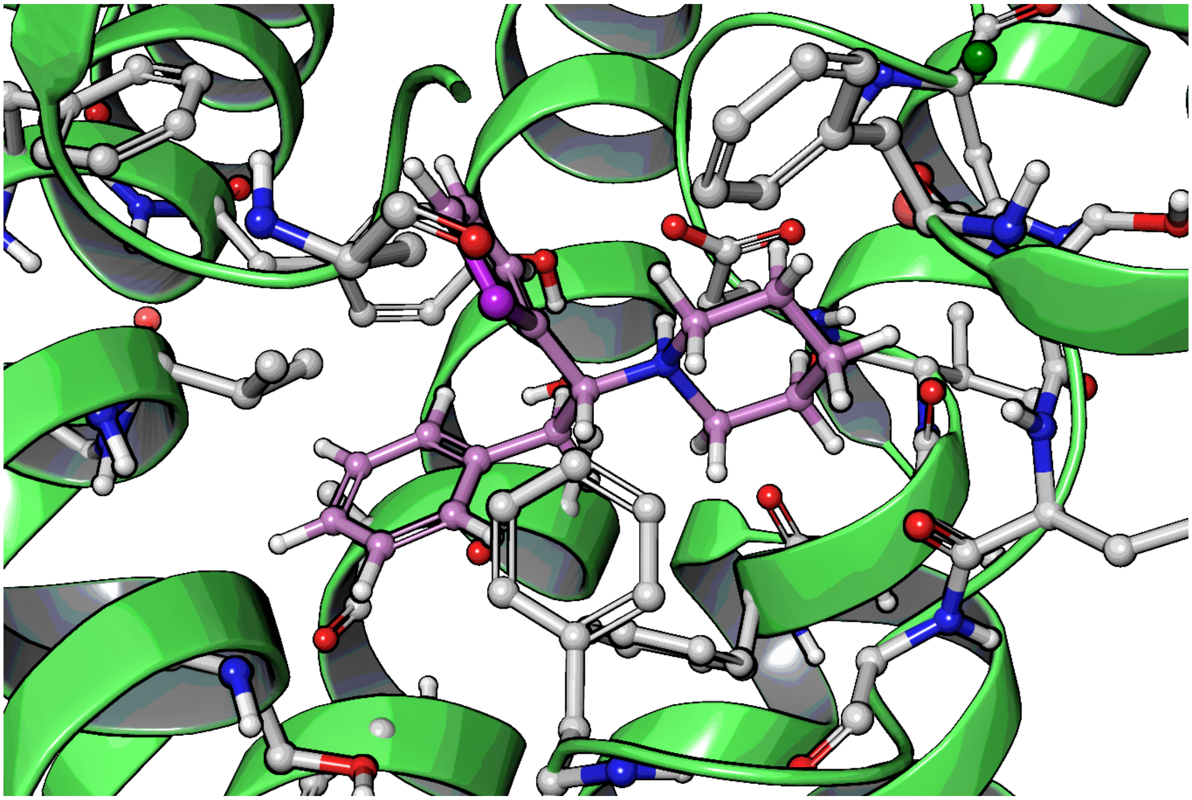

Figure S3M. Induced Fit Docking Pose for (R)-SePP (purple) at the DAT (PDB: 8Y2G).

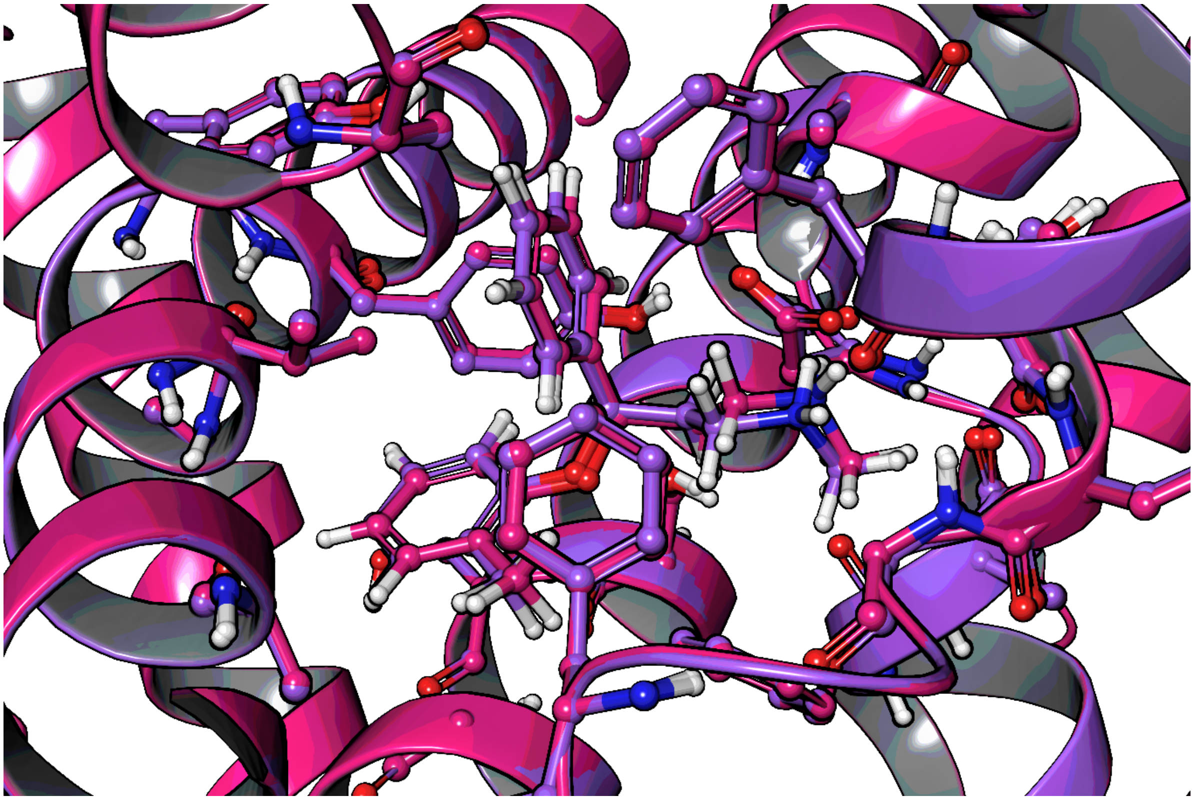

Figure S4A. Induced Fit Docking Pose of Atomoxetine (pink) at the NET (PDB: 8Z1L) Overlaid with its Experimental structure (purple) (PDB: 8Z1L), RMSD = 0.358Å.

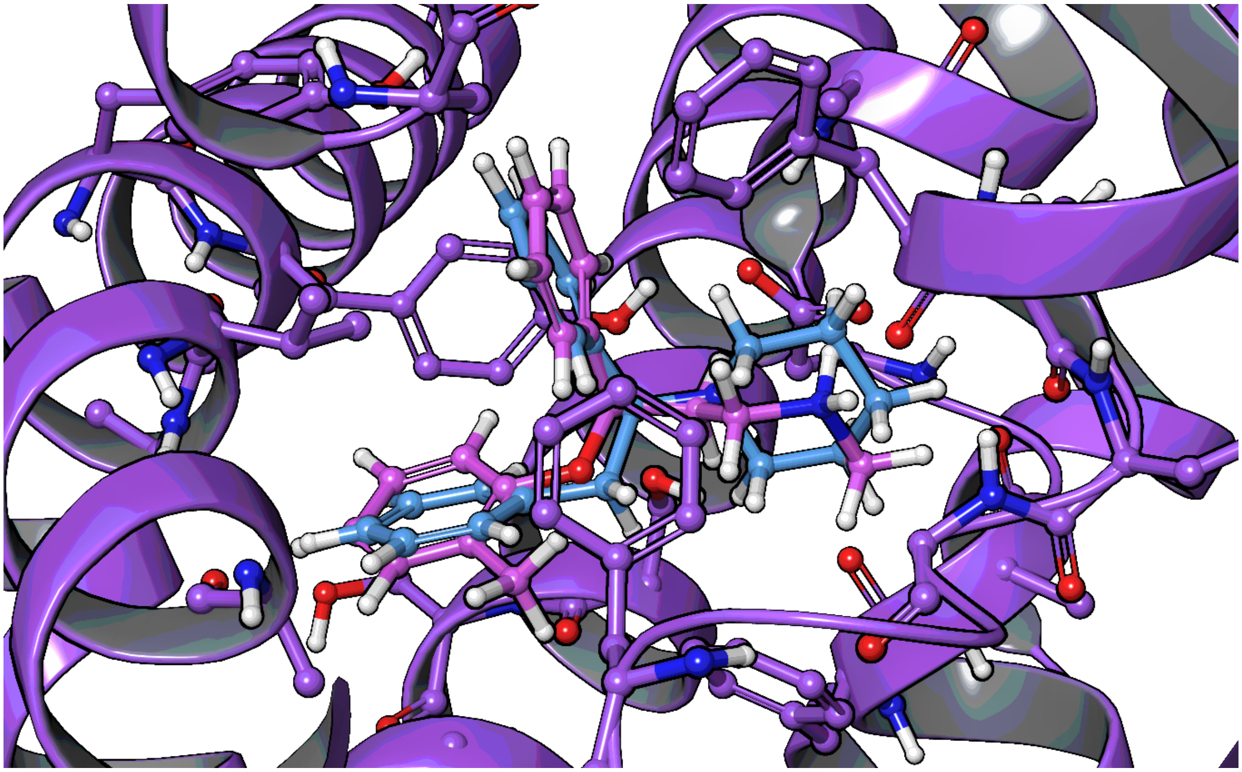

Figure S4B. Induced Fit Docking Pose for (S)-Diphenidine Pose (blue) at the NET (PDB: 8Z1L) Overlaid with the Atomoxetine NET Experimental Structure (purple) (PDB: 8Z1L).

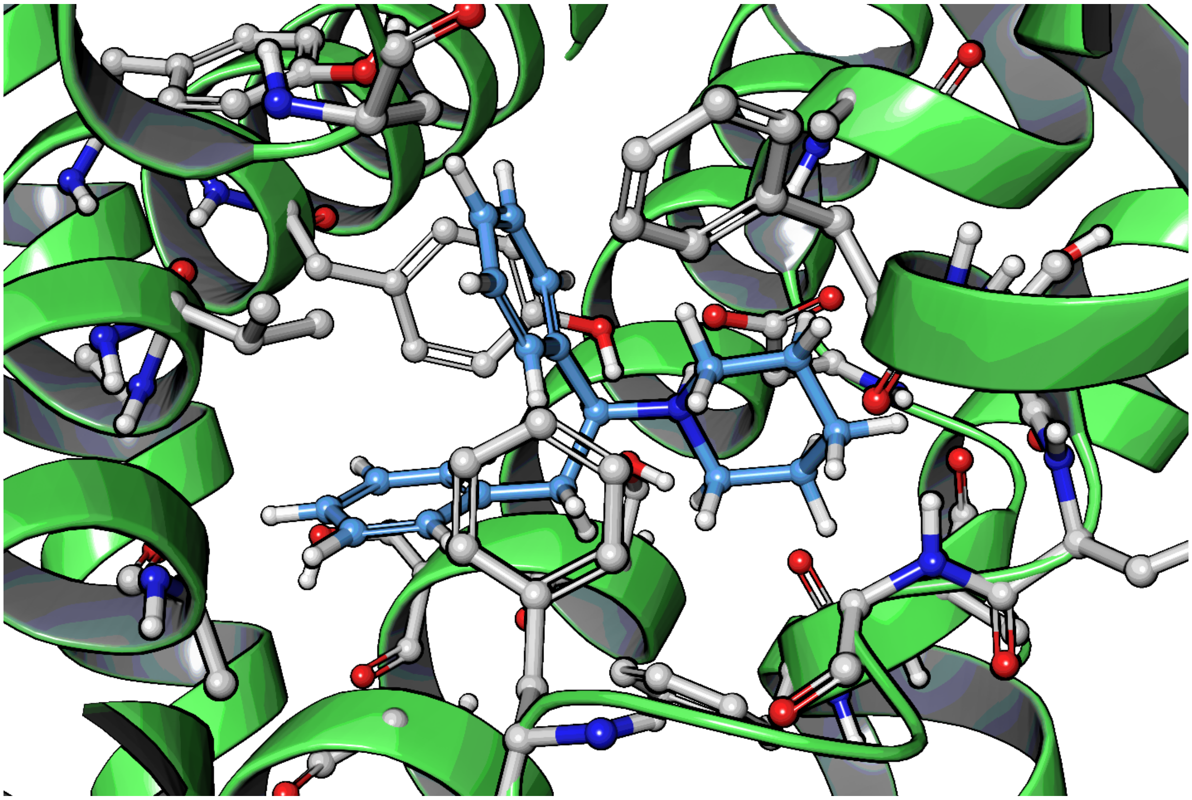

Figure S4C. Induced Fit Docking Pose for (S)-Diphenidine (blue) at the NET (PDB: 8Z1L).

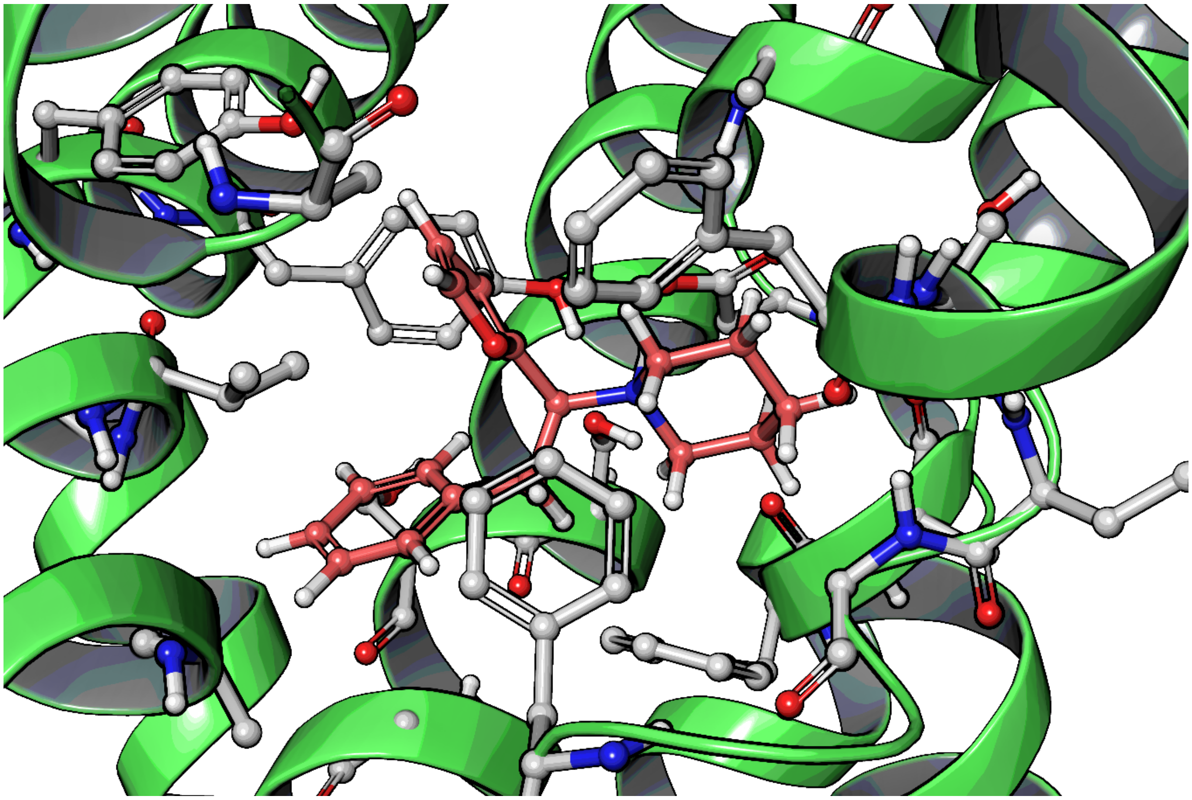

Figure S4D. Induced Fit Docking Pose for (S)-FuPP (red) at the NET (PDB: 8Z1L).

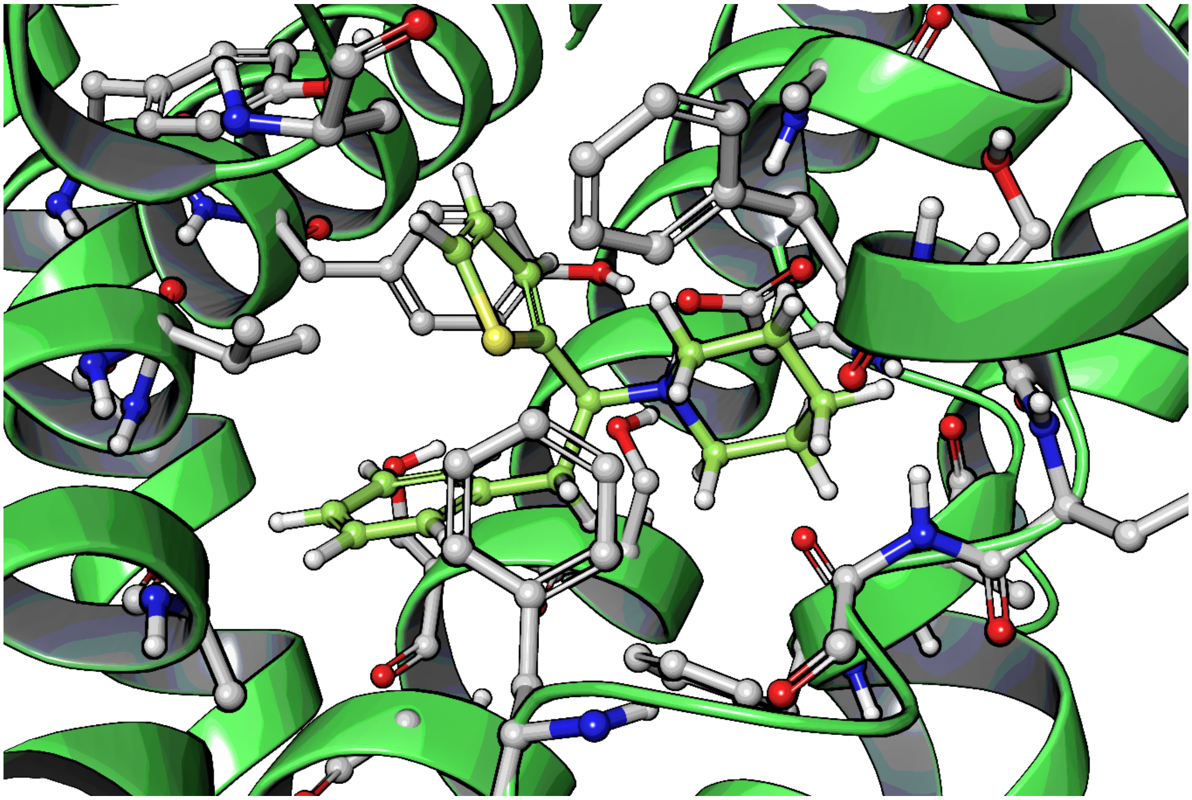

Figure S4E. Induced Fit Docking Pose for (S)-TPP (green) at the NET (PDB: 8Z1L).

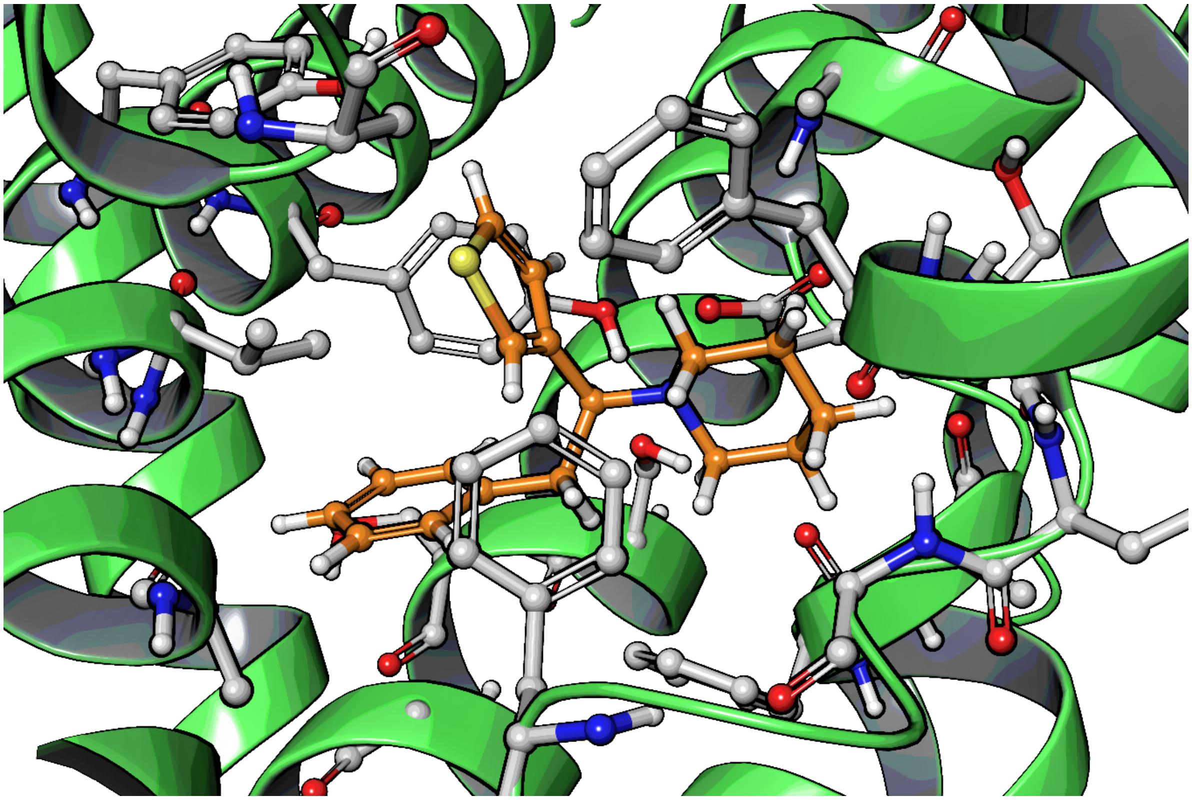

Figure S4F. Induced Fit Docking Pose for (S)-3-TPP (orange) at the NET (PDB: 8Z1L).

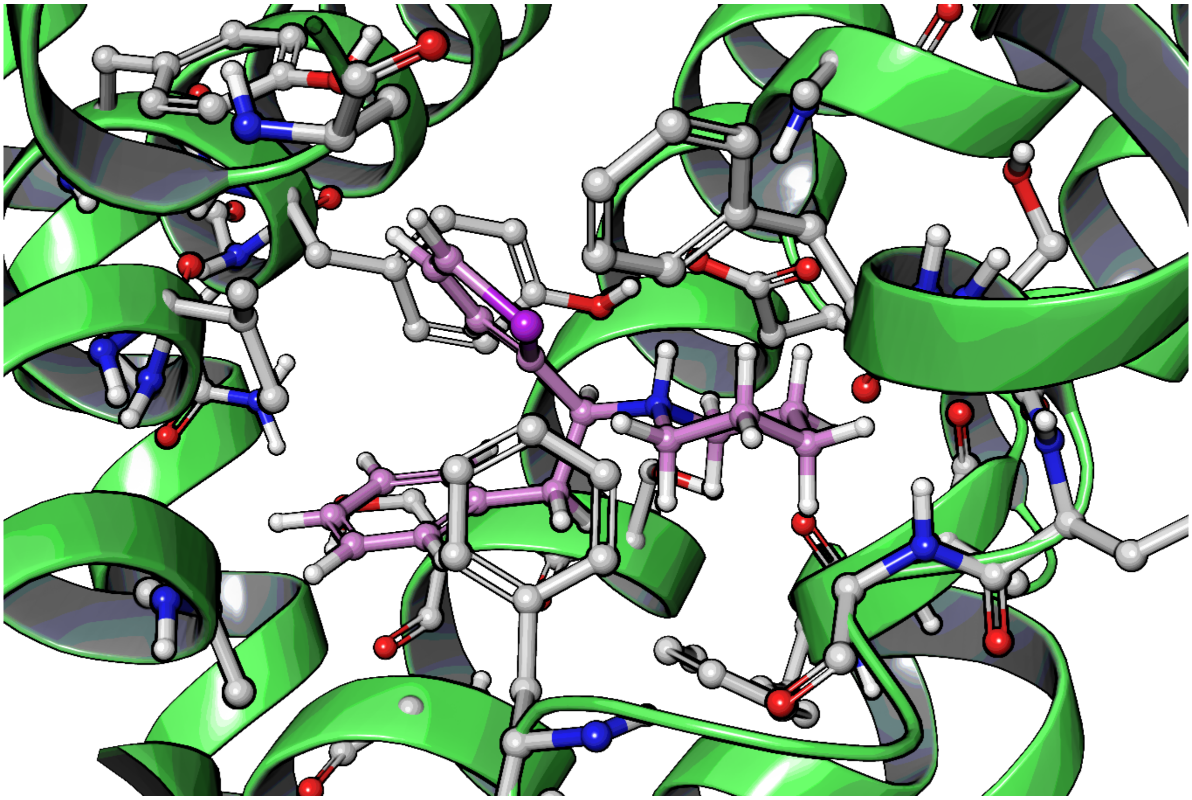

Figure S4G. Induced Fit Docking Pose for (S)-SePP (purple) at the NET (PDB: 8Z1L).

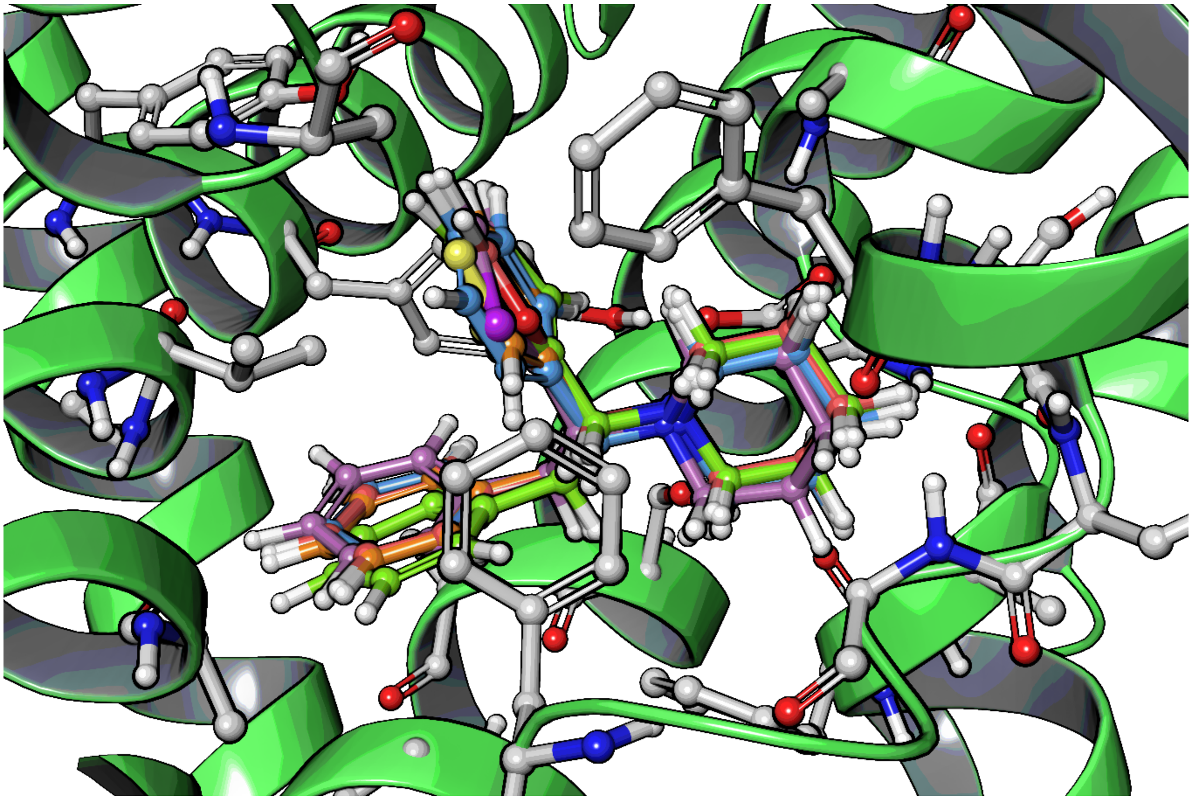

Figure S4H. Overlay of Induced Fit Docking (R)-Diarylethylamines Poses at the NET (PDB: 8Z1L). Diphenidine (blue), (R)-FPP (red), (R)-TPP (green), (R)-3-TPP (orange) and (R)-SePP (purple).

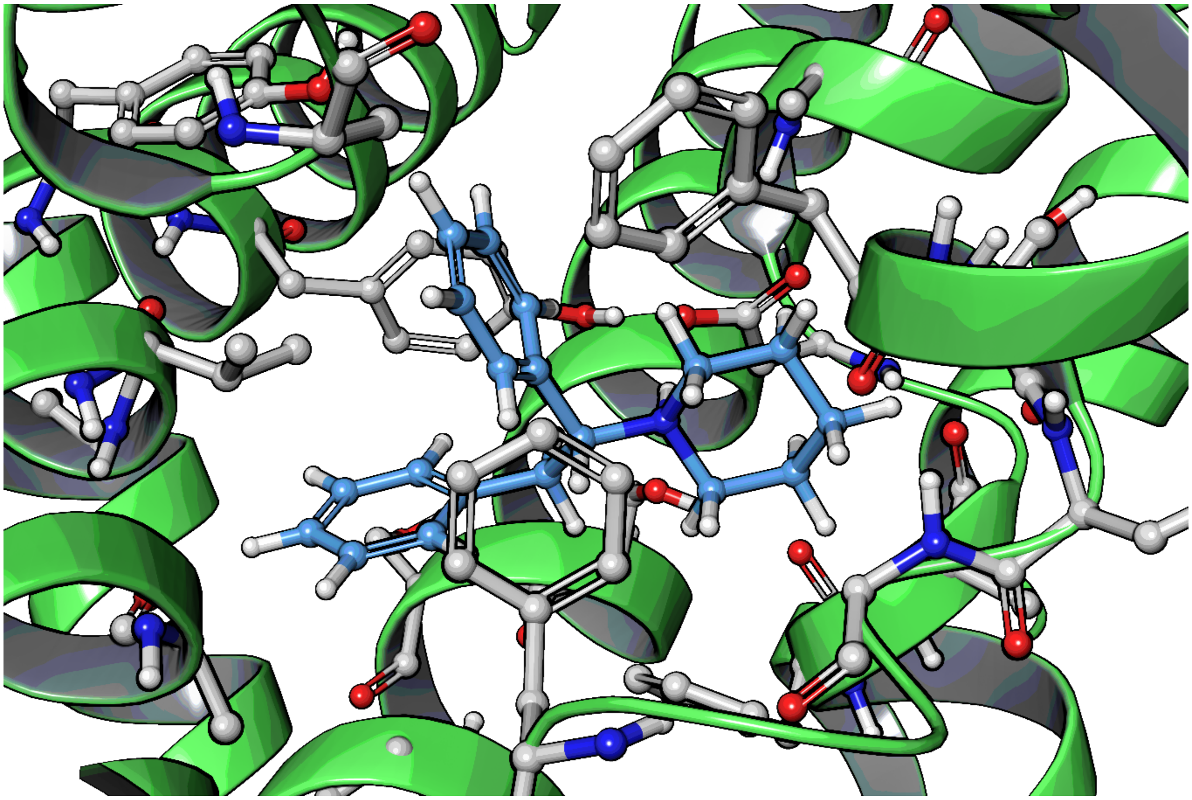

Figure S4I. Induced Fit Docking Pose for (R)-Diphenidine at the NET (PDB: 8Z1L).

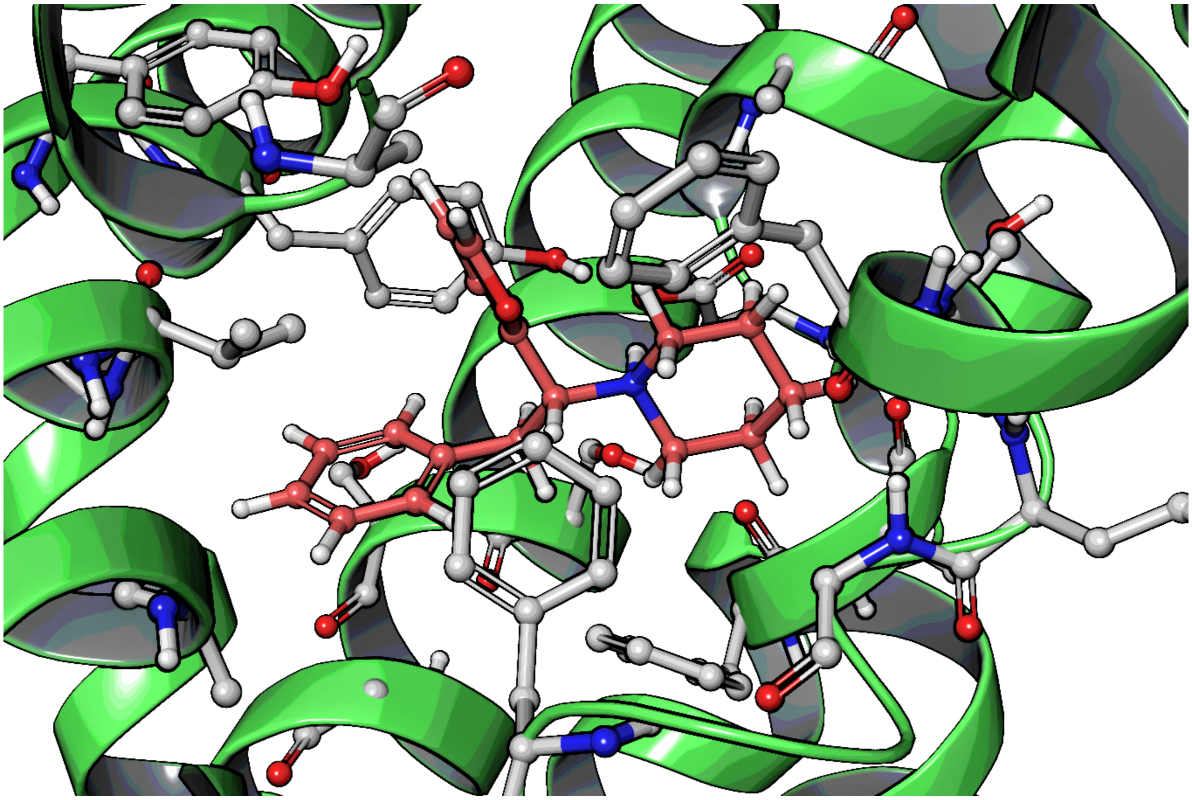

Figure S4J. Induced Fit Docking Pose for (R)-FuPP (red) at the NET (PDB: 8Z1L).

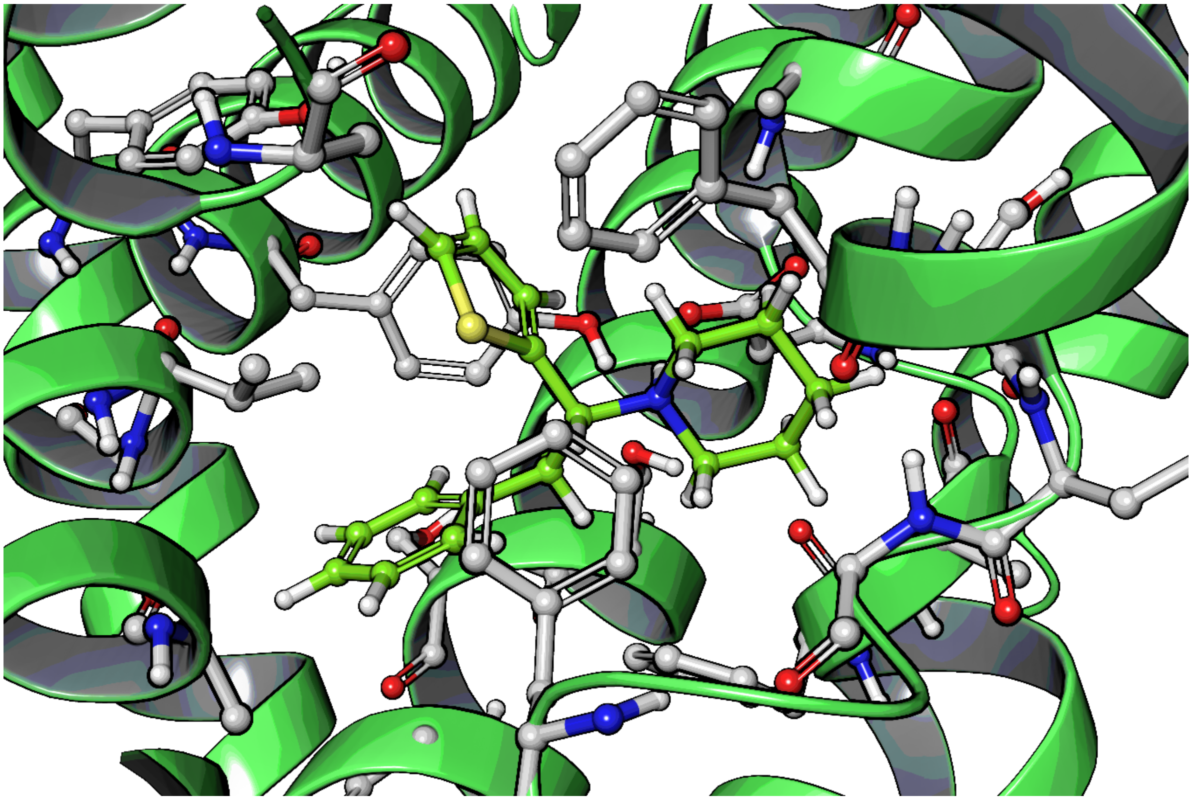

Figure S4K. Induced Fit Docking Pose for (R)-TPP (green) at the NET (PDB: 8Z1L).

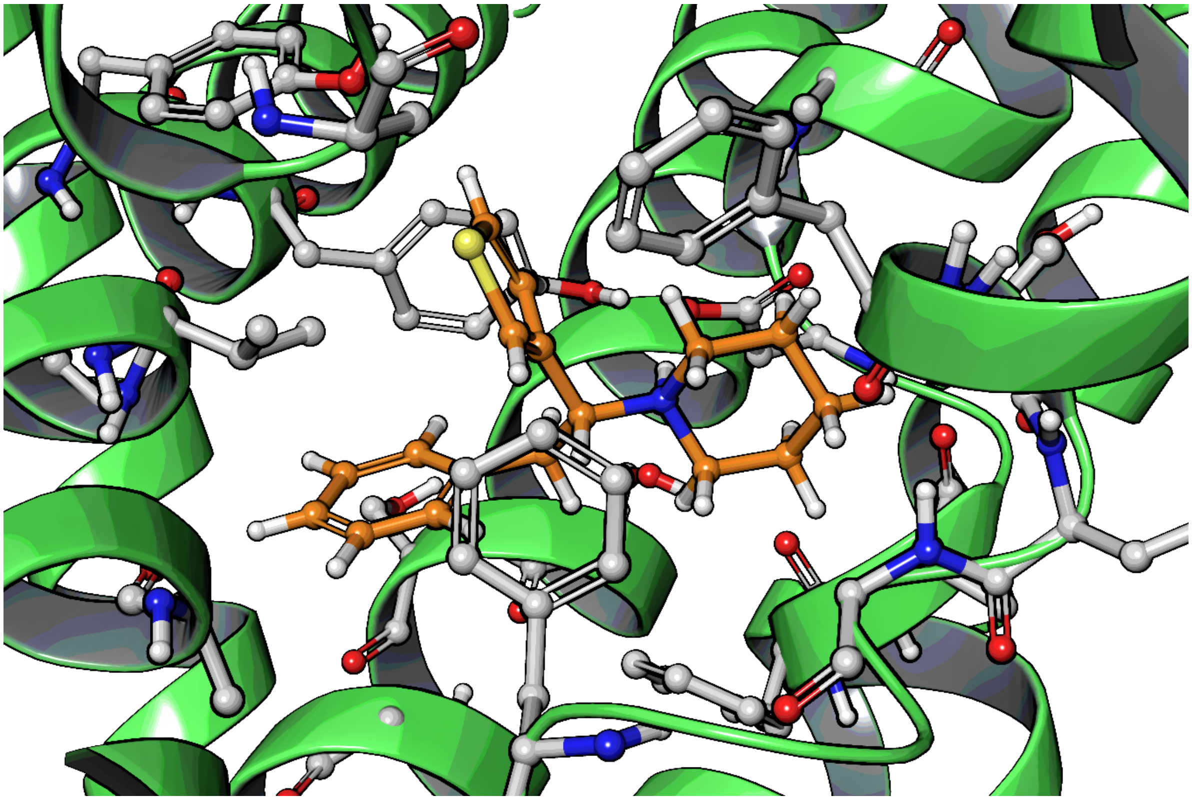

Figure S4L. Induced Fit Docking Pose for (R)-3-TPP (orange) at the NET (PDB: 8Z1L).

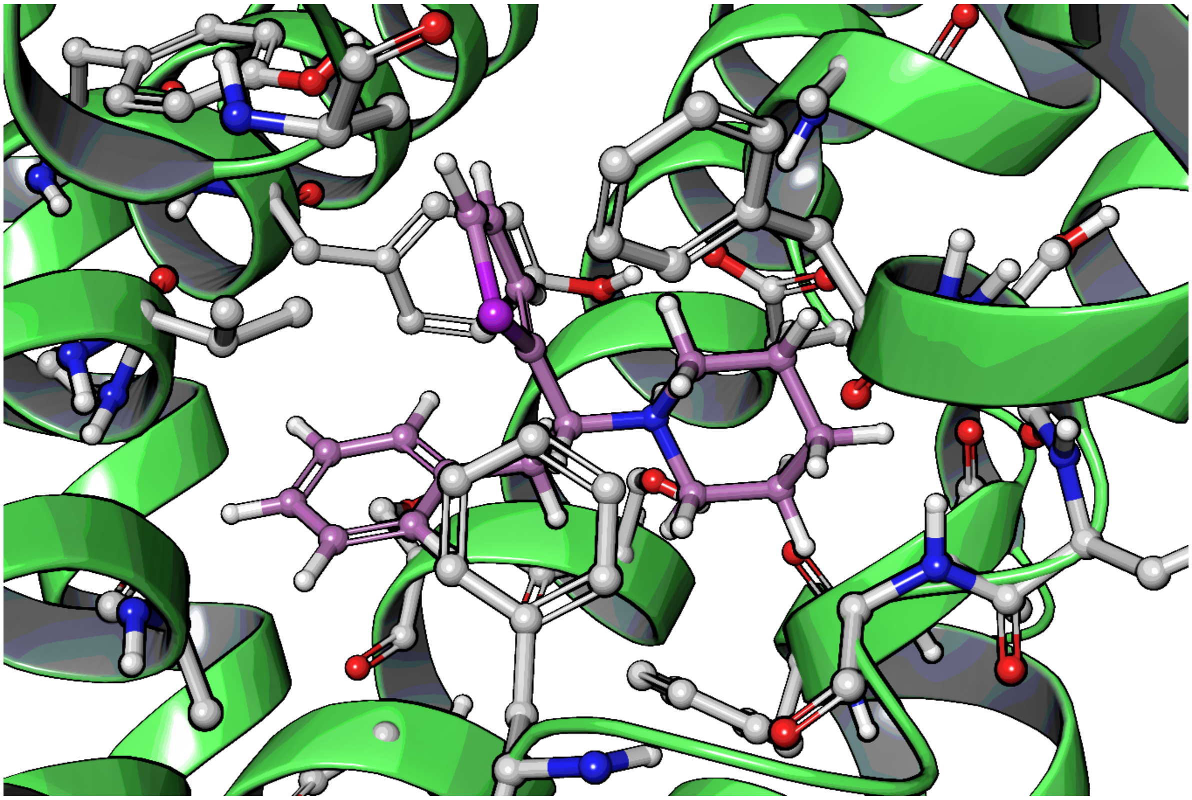

Figure S4M. Induced Fit Docking Pose for (R)-SePP (purple) at the NET (PDB: 8Z1L).

HPLC Traces

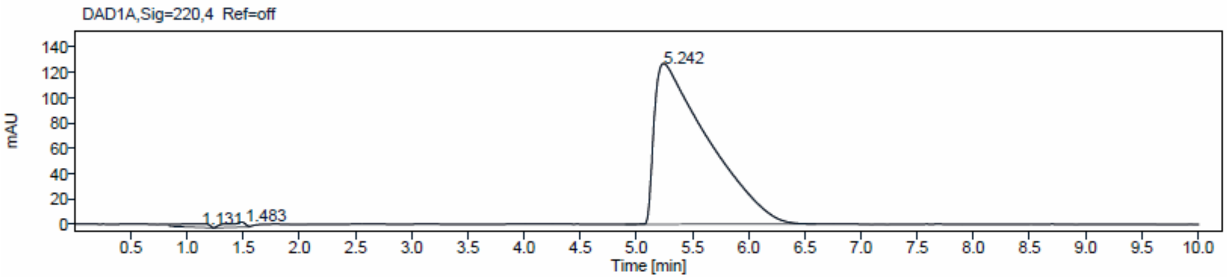

Figure S5A. Diphenidine HPLC Trace. 1 mg/mL Diphenidine HCl with 70% 10 mM aqueous ammonium formate buffer titrated to pH 4.5 with 10 mM formic acid and 30% HPLC grade acetonitrile at 220 nm.

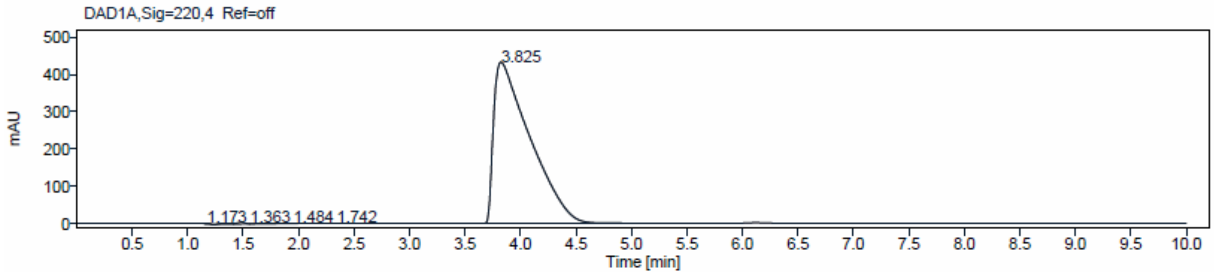

Figure S5B. FuPP HPLC Trace. 1 mg/mL FuPP HCl with 70% 10 mM aqueous ammonium formate buffer titrated to pH 4.5 with 10 mM formic acid and 30% HPLC grade acetonitrile at 220 nm.

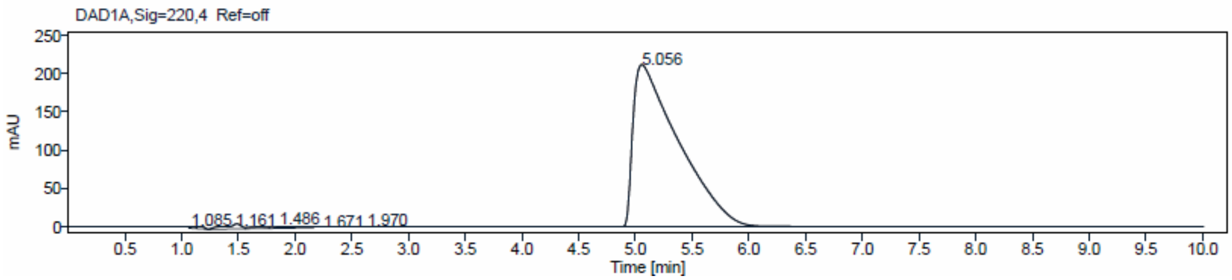

Figure S5C. TPP HPLC Trace. 1 mg/mL TPP HCl with 70% 10 mM aqueous ammonium formate buffer titrated to pH 4.5 with 10 mM formic acid and 30% HPLC grade acetonitrile at 220 nm.

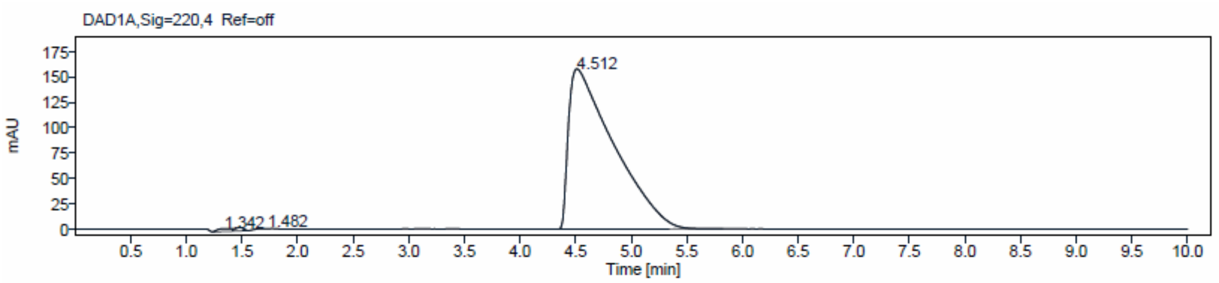

Figure S5D. 3-TPP HPLC Trace. 1 mg/mL 3-TPP HCl with 70% 10 mM aqueous ammonium formate buffer titrated to pH 4.5 with 10 mM formic acid and 30% HPLC grade acetonitrile at 220 nm.

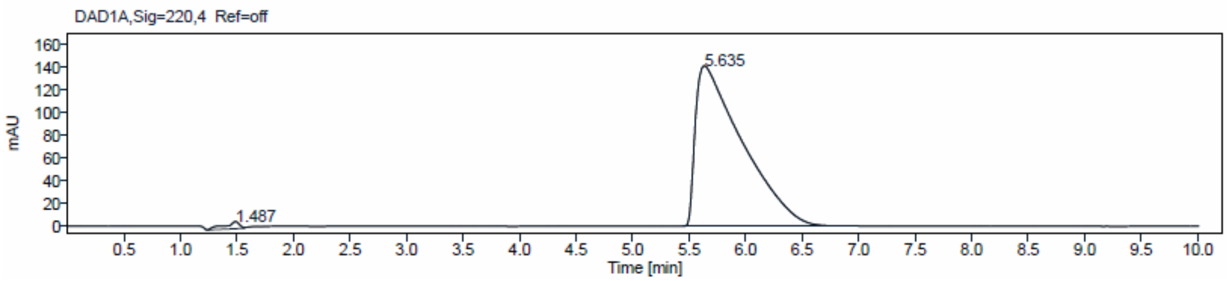

Figure S5E. SePP HCl HPLC Trace. 1 mg/mL SePP HCl with 70% 10 mM aqueous ammonium formate buffer titrated to pH 4.5 with 10 mM formic acid and 30% HPLC grade acetonitrile at 220 nm.

<u>**NMR Spectra**</u>

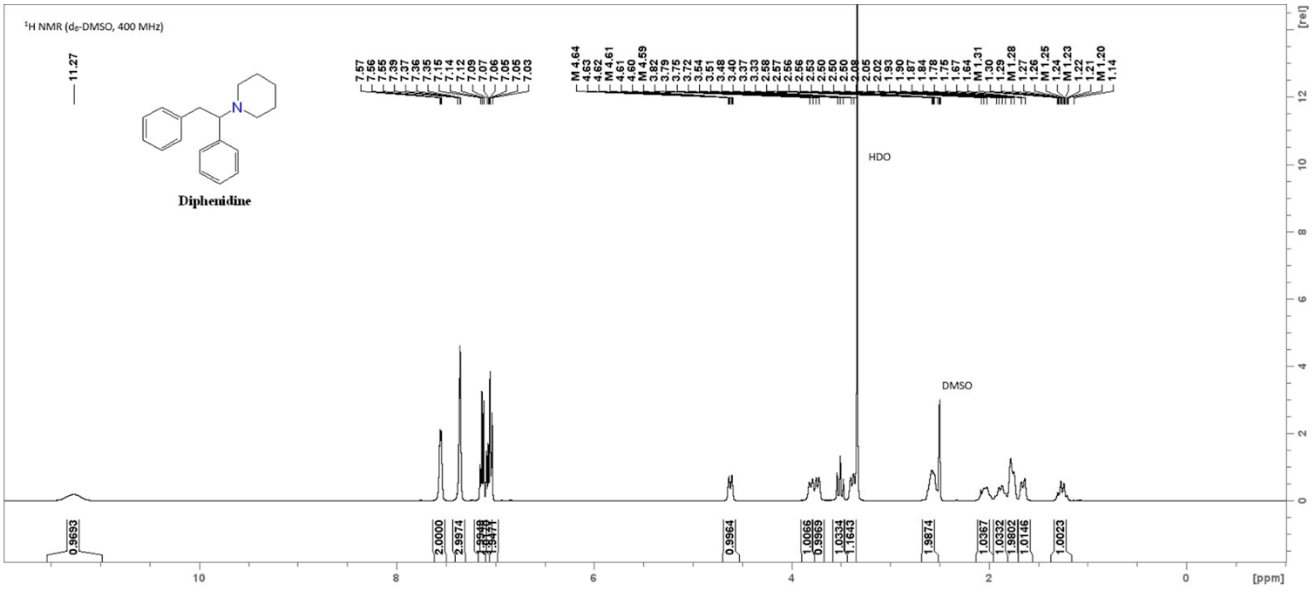

Figure S6A. ^{1}H NMR of Diphenidine HCl (400 MHz, ~20 mg/mL in d_6-DMSO).

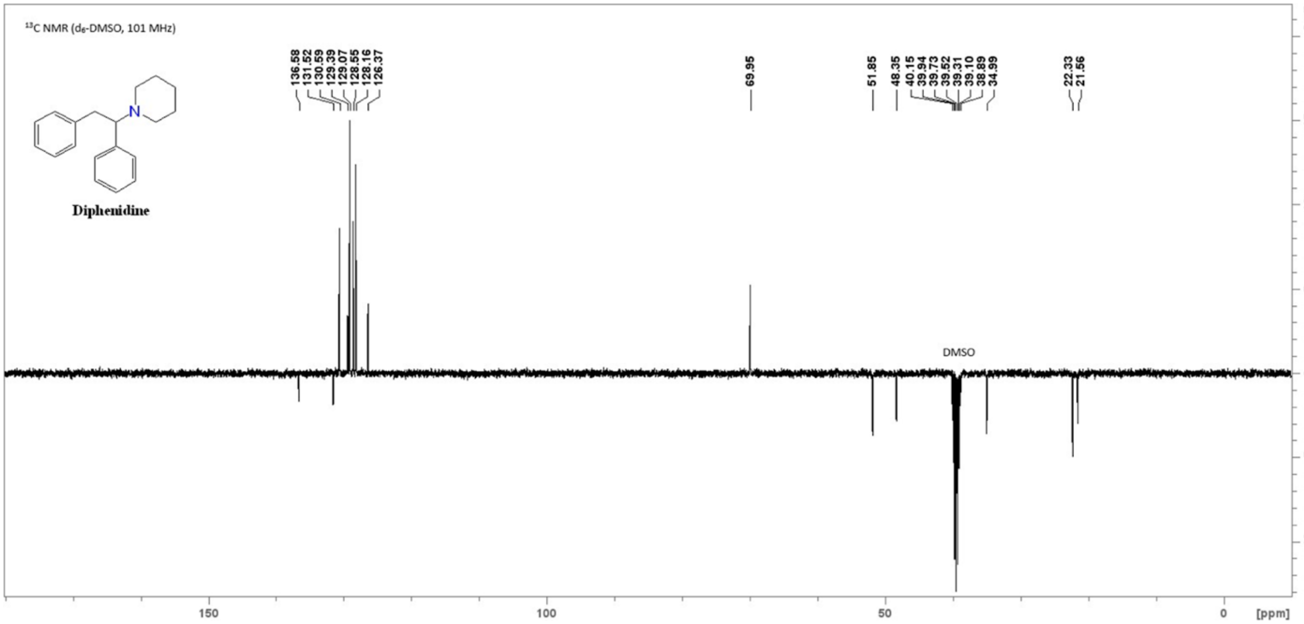

Figure S6B. ^{13}C NMR of Diphenidine HCl (101 MHz, ~20 mg/mL in d_6-DMSO).

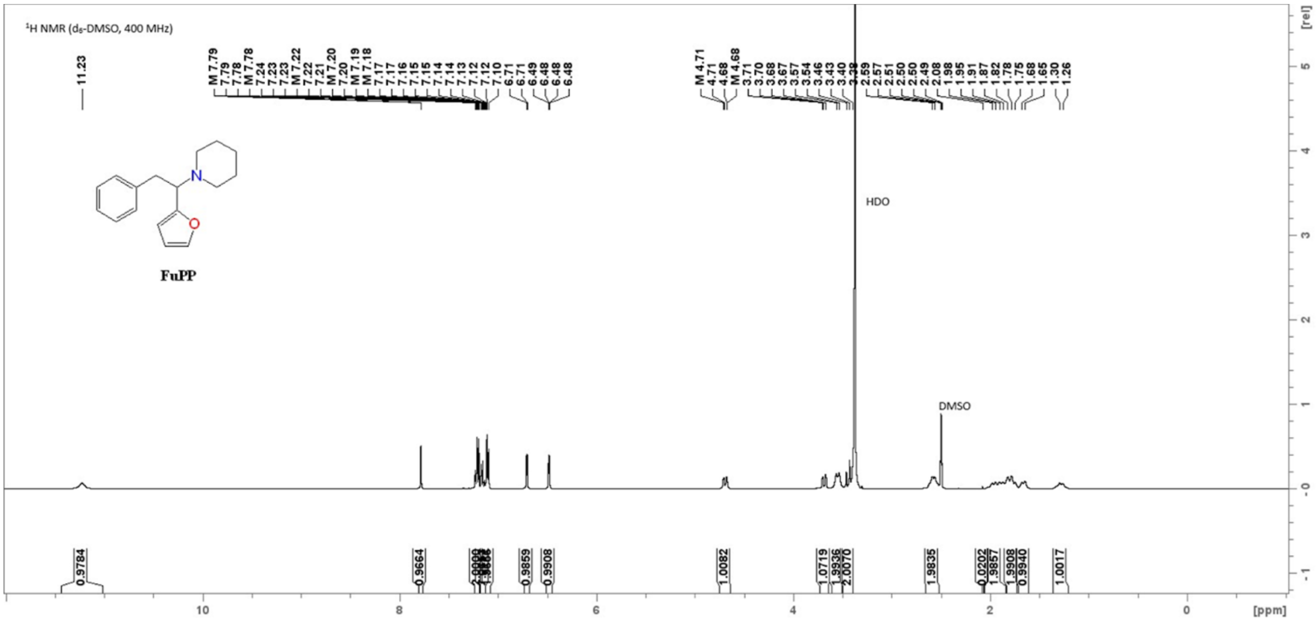

Figure S6C. ^{1}H NMR of FuPP HCl (400 MHz, ~20 mg/mL in d_6-DMSO).

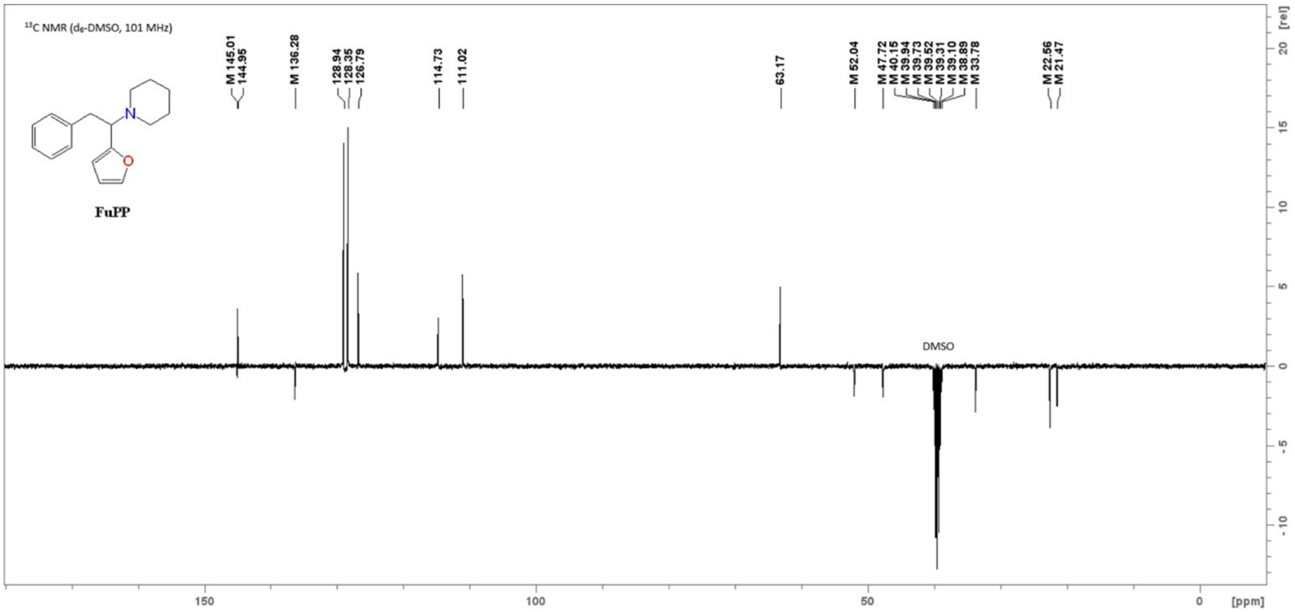

Figure S6D. ^{13}C NMR of FuPP HCl (101 MHz, ~20 mg/mL in d_6-DMSO).

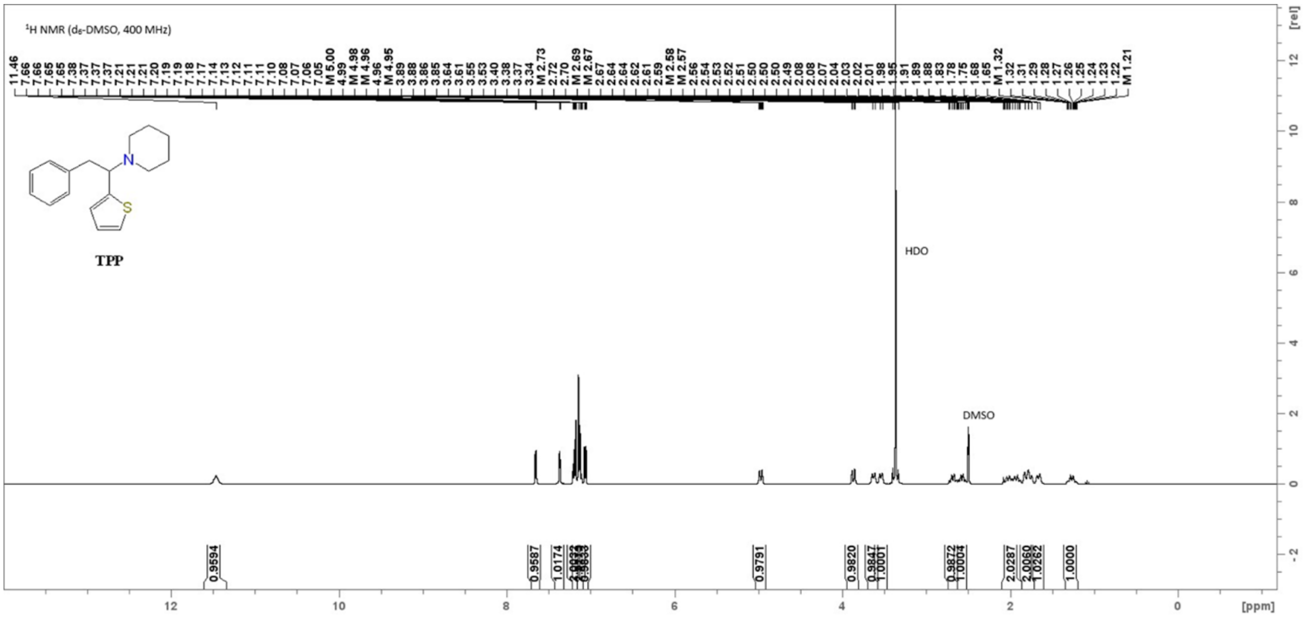

Figure S6E. ^{1}H NMR of TPP HCl (400 MHz, ~20 mg/mL in d_6-DMSO).

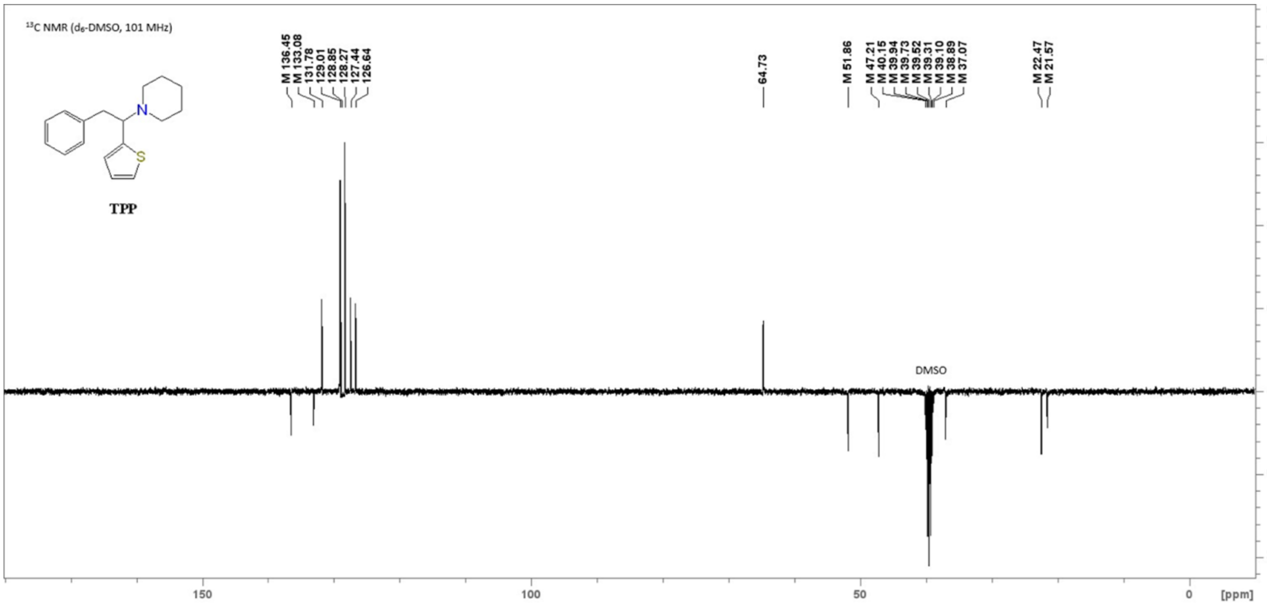

Figure S6F. ^{13}C NMR of TPP HCl (101 MHz, ~20 mg/mL in d$_6$-DMSO).

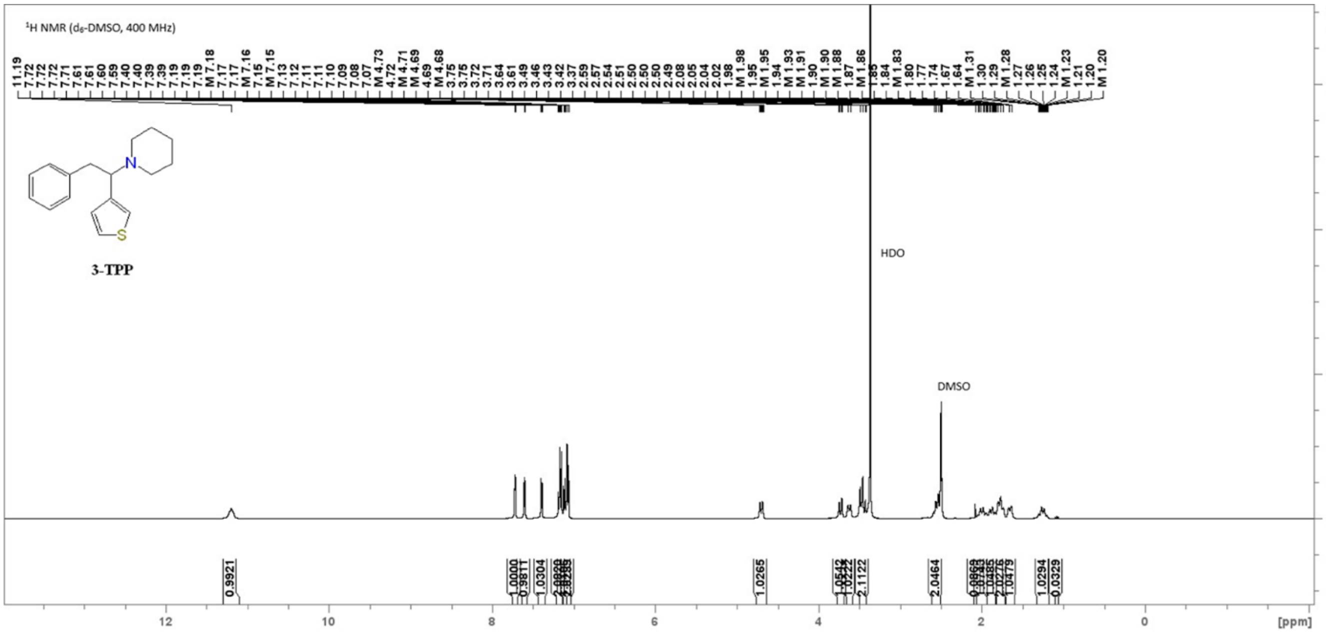

Figure S6G. ^{1}H NMR of 3-TPP HCl (400 MHz, ~20 mg/mL in d$_6$-DMSO).

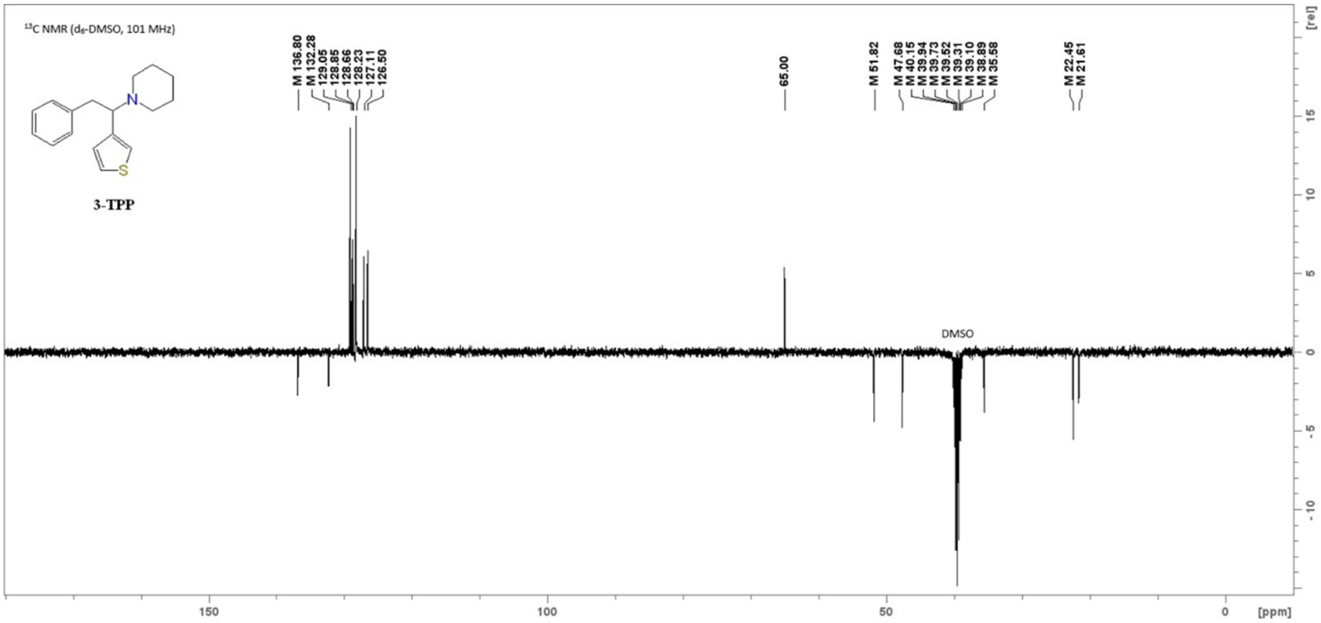

Figure S6H. ^{13}C NMR of 3-TPP HCl (101 MHz, ~20 mg/mL in d_6-DMSO).

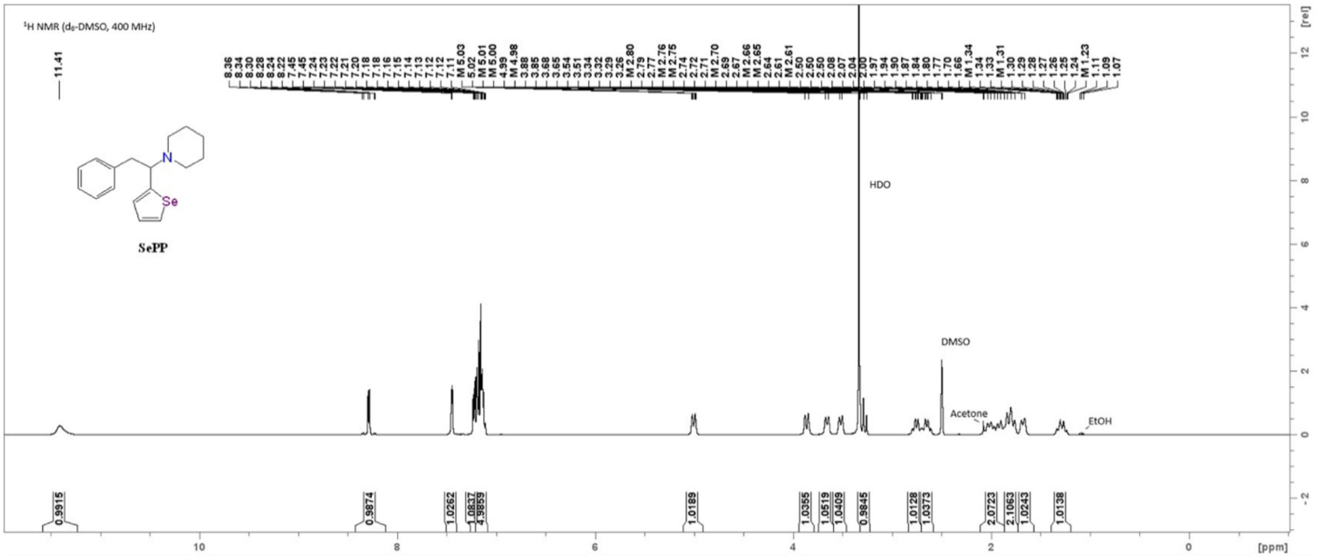

Figure S6I. ^{1}H NMR of SePP HCl (400 MHz, ~20 mg/mL in d_6-DMSO).

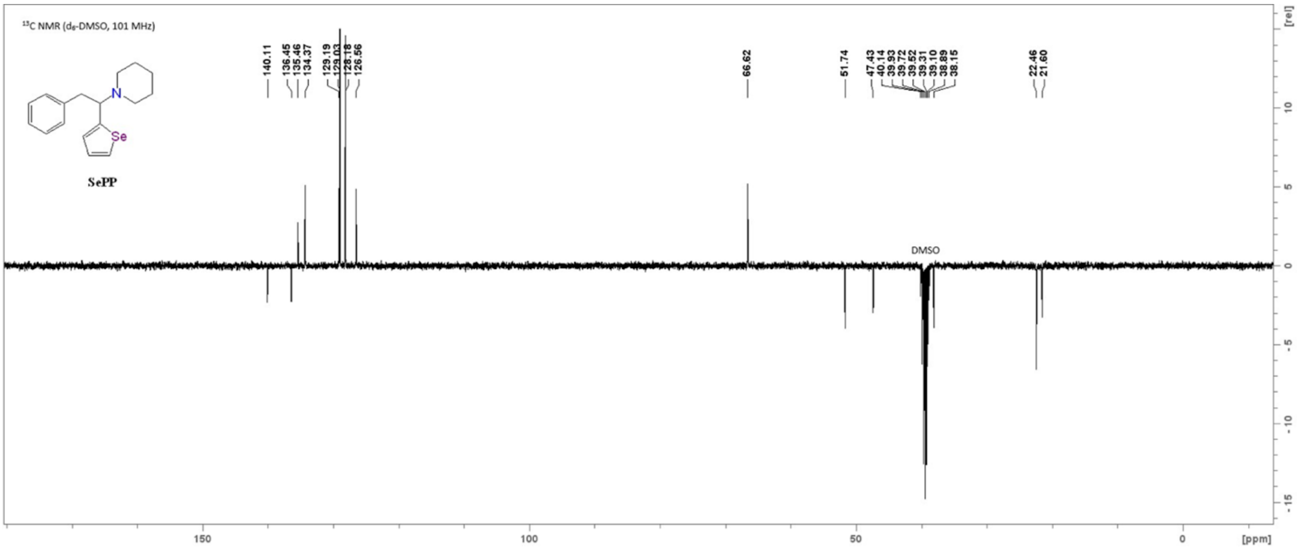

Figure S6J. ^{13}C NMR of SePP HCl (101 MHz, ~20 mg/mL in d_6-DMSO).

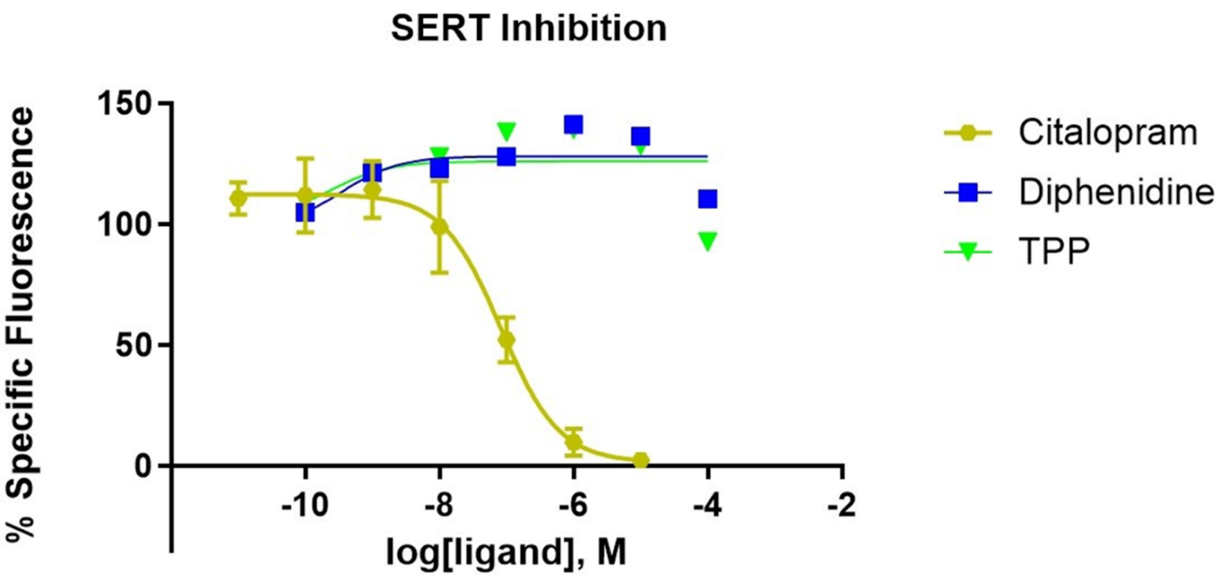

Figure S7. SERT reuptake inhibition Concentration Response Curves for Citalopram (ref, N = 7), diphenidine and TPP (N = 1).

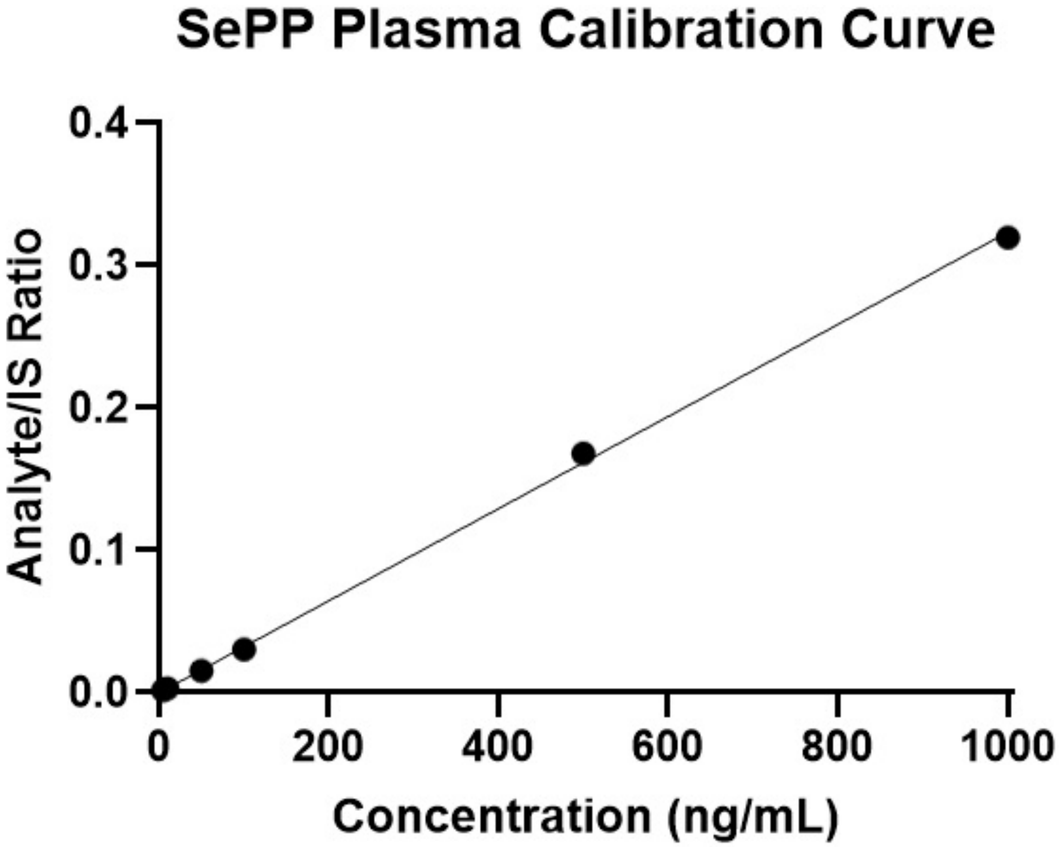

Fig S8. Calibration Curve for SePP in Plasma. The analyte/internal standard (IS) peak area ratio is plotted against concentration (ng/mL) of analyte. Weighted linear regression using a weighing factor of 1/x, where x is the analyte's concentration was used to assess assay linearity across the tested concentration range. $R^2 = 0.9991$, $y = 0.0003236x + 0.00003236$.

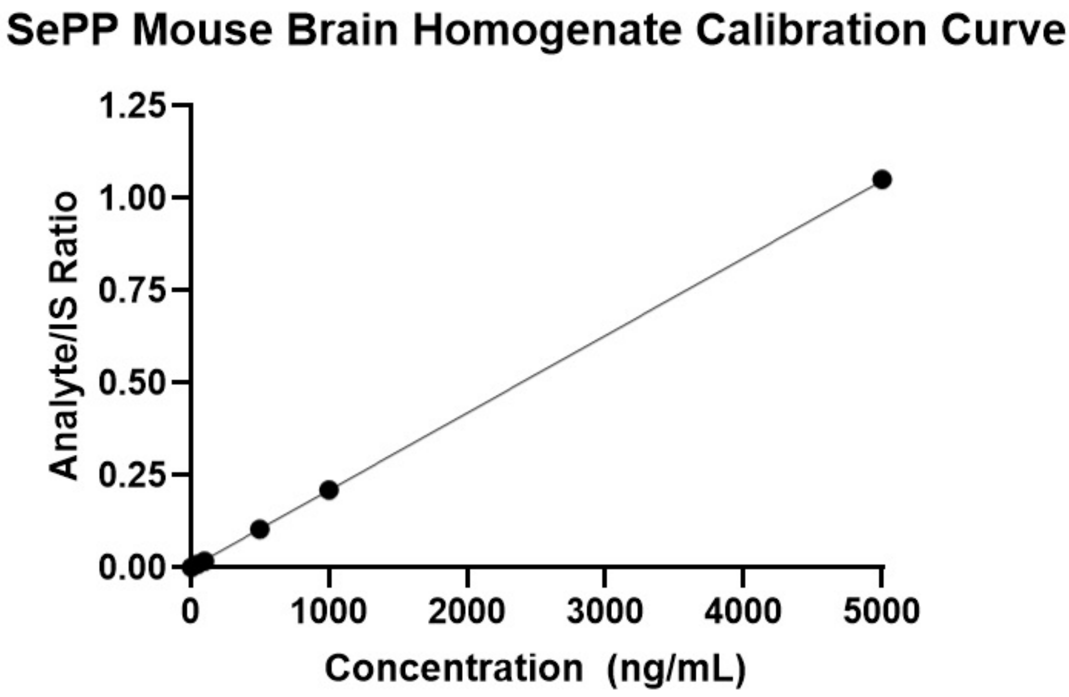

Fig S9. Calibration Curve for SePP in Mouse Whole Brain Homogenate. The analyte/internal standard (IS) peak area ratio is plotted against concentration (ng/mL) of analyte. Weighted linear regression using a weighing factor of 1/x, where x is the analyte's concentration was used to assess assay linearity across the tested concentration range. $R^2 = 0.9996$, $y = 0.0002094x + 0.0006144$.

Supporting Information Tables

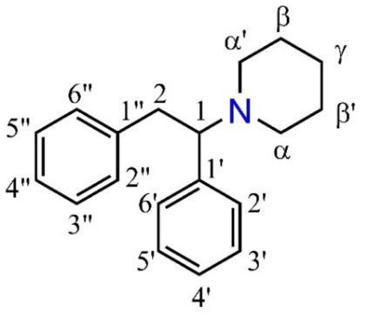

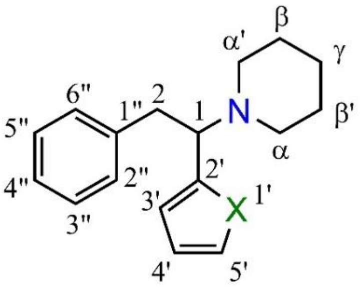

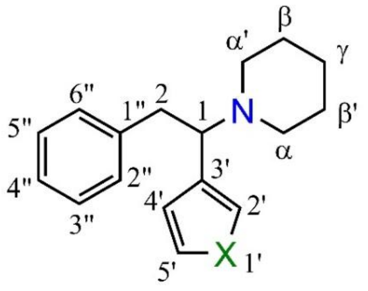

Table S1. ^{1}H NMR Chemical Shift Assignments. Spectra recorded on HCl salts (~20 mg/mL in d_6-DMSO).					
[1]H Shift	DPP	FuPP	TPP	3-TPP	SePP
H_1	4.62 (dt, *J* = 12.1, 3.5 Hz, 1H)	4.69 (dd, *J* = 12.2, 1.7 Hz, 1H)	4.97 (dt, *J* = 12.2, 2.8 Hz, 1H)	4.70 (dt, *J* = 12.2, 2.9 Hz, 1H)	5.01 (dt, *J* = 12.2, 3.2 Hz, 1H)
H_2	3.80 (d, *J* = 12.9 Hz, 1H)<br>3.51 (t, *J* = 12.6 Hz, 1H)	3.69 (dd, *J* = 12.8, 2.6 Hz, 1H)<br>3.43 (t, *J* = 12.7 Hz, 1H)	3.87 (dd, *J* = 13.0, 2.3 Hz, 1H)<br>3.37 (t, *J* = 12.6 Hz, 1H)	3.73 (dd, *J* = 12.8, 2.5 Hz, 1H)<br>3.46 (t, *J* = 12.6 Hz, 1H)	3.87 (dd, *J* = 13.2, 2.9 Hz, 1H)<br>3.29 (t, *J* = 12.5 Hz, 1H)
$H_{\alpha,\alpha'}$	3.73 (d, *J* = 11.8 Hz, 1H)<br>3.39 (d, *J* = 12.1 Hz, 1H)<br>2.68-2.52 (m, 2H)	3.55 (d, *J* = 11.3 Hz, 2H)<br>2.66-2.52 (m, 2H)	3.63 (d, *J* = 11.6 Hz, 1H)<br>3.54 (d, *J* = 11.6 Hz, 1H)<br>2.68 (qd, *J* = 11.5, 2.9 Hz, 1H)<br>2.58 (qd, *J* = 11.4, 2.6 Hz, 1H)	3.62 (d, *J* = 11.7 Hz, 1H)<br>3.53-3.38 (m, 1H) *Coalescing with H_β<br>2.62-2.51 (m, 2H)	3.66 (d, *J* = 12.2 Hz, 1H)<br>3.52 (d, *J* = 12.1 Hz, 1H)<br>2.75 (qd, *J* = 9.2, 3.2 Hz, 1H)<br>2.65 (qd, *J* = 9.3, 3.2 Hz, 1H)
$H_{\beta,\beta'}$	2.13-1.96 (m, 1H)<br>1.96-1.81 (m, 1H)	2.06-1.85 (m, 2H)<br>1.84-1.73 (m, 2H)	2.10-1.86 (m, 2H)<br>1.86-1.72 (m, 2H)	2.00 (qt, *J* = 13.5, 3.9 Hz, 1H)<br>1.89 (qt, *J* = 13.4, 3.6 Hz, 1H)<br>1.83-1.72 (m, 2H)	2.11 – 1.89 (m, 2H)<br>1.88-1.74 (m, 2H)

	1.81-1.71 (m, 2H)				
H_γ	1.65 (d, *J* = 13.0 Hz, 1H)<br><br>1.26 (qt, *J* = 13.0, 3.8 Hz, 1H)	1.66 (d, *J* = 12.4 Hz, 1H)<br><br>1.37-1.21 (m, 1H)	1.66 (ad, *J* = 13.2 Hz, 1H)<br><br>1.27 (qt, *J* = 13.1, 3.8 Hz, 1H)	1.71-1.60 (m, 1H)<br><br>1.25 (qt, *J* = 12.9, 3.8 Hz, 1H)	1.68 (d, *J* = 13.2 Hz, 1H)<br><br>1.29 (qt, *J* = 13.0, 3.8 Hz, 1H)
$H_{1'}$	-	-	-	-	-
$H_{2'}$	7.62-7.50 (m, 1H)	-	-	7.40 (dd, *J* = 5.0, 1.2 Hz, 1H)	-
$H_{3'}$	7.41-7.31 (m, 1H)<br>*coalescing with $H_{2',6'}$	6.71 (d, *J* = 3.0 Hz, 1H)	7.37 (dd, *J* = 3.5, 1.0 Hz, 1H)	-	7.45 (dd, *J* = 3.8, 1.2 Hz, 1H)
$H_{4'}$	7.41-7.31 (m, 1H)<br>*coalescing with $H_{2',6'}$	6.48 (dd, *J* = 3.3, 1.9 Hz, 1H)	7.07 (dd, *J* = 5.1, 3.6 Hz, 1H)	7.72 (dd, *J* = 2.9 Hz, 1.1 Hz, 1H)	7.22 (dd, *J* = 5.4, 3.7, 1H)<br><br>*coalescing with $H_{2'',6''}$ $H_{3'',5''}$ and $H_{4''}$
$H_{5'}$	7.41-7.31 (m, 1H)<br>*coalescing with $H_{2',6'}$	7.78 (dd, *J* = 1.9, 0.8 Hz, 1H)	7.65 (dd, *J* = 5.1, 1.0 Hz, 1H)	7.60 (dd, *J* = 5.0, 2.9 Hz, 1H)	8.29 (dd, *J* = 5.6, 1.2 Hz, 1H)
$H_{6'}$	7.62-7.50 (m, 1H)	-	-	-	-
$H_{1''}$	-	-	-	-	-
$H_{2'',6''}$	7.04 (ad, *J* = 7.4 Hz, 2H)	7.11 (ad, *J* = 7.7 Hz, 2H)	7.14 (ad, *J* = 6.9 Hz, 2H)<br>*Coalescing with $H_{4''}$	7.09 (ad, *J* = 7.7 Hz, 2H)	7.21 – 7.10 (m, 2H)<br>*Coalescing with $H_{3'',5''}$ and $H_{4''}$
$H_{3'',5''}$	7.14 (at, *J* = 7.5 Hz, 2H)	7.21 (att, *J* = 7.1, 1.5 Hz, 2H)	7.23-7.16 (m, 2H)	7.17 (att, *J* = 7.1, 1.4 Hz, 2H)	7.21 – 7.10 (m, 2H)<br>*Coalescing with $H_{2'',6''}$ and $H_{4''}$
$H_{4''}$	7.10-7.06 (m, 1H)	7.18-7.13 (m, 1H)	7.16-7.10 (m, 1H)<br>*Coalescing with $H_{2'',6''}$	7.14-7.10 (m, 1H)	7.21 – 7.10 (m, 1H)<br><br>*Coalescing with $H_{2'',6''}$ and $H_{3'',5''}$
NH^+	11.27 (s, $1NH^+$)	11.23 (s, $1NH^+$)	11.46 (s, $1NH^+$)	11.19 (s, $1NH^+$)	11.41 (s, $1NH^+$)

**Table S2. ^{13}C NMR Chemical Shift Assignments. Spectra recorded on HCl salts (~20 mg/mL in d_6-DMSO).**					
**^{13}C Shift**	**DPP**	**FuPP**	**TPP**	**3-TPP**	**SePP**
C_1	70.0	63.2	64.7	65.0	66.6
C_2	35.0	33.8	37.1	35.6	38.2
C_α	51.9<br>48.4	51.8<br>47.7	51.9<br>47.2	51.8<br>47.7	51.7<br>47.4
C_β	22.3	22.5	22.5	22.5	22.5
C_γ	21.6	21.5	21.6	21.6	21.6
$C_{1'}$	131.5	-	-	-	-
$C_{2'}$	130.6	145.0	133.1	128.7	140.1
$C_{3'}$	128.6	114.7	131.8	132.3	134.4
$C_{4'}$	129.4	111.0	127.4	128.9	129.2
$C_{5'}$	128.6	145.0	128.9	127.1	135.5
$C_{6'}$	130.6	-	-	-	-
$C_{1''}$	136.6	136.3	136.5	136.8	136.5
$C_{2'',6''}$	129.1	128.9	129.0	129.1	129.0
$C_{3'',5''}$	128.2	128.4	128.3	128.2	128.2
$C_{4''}$	126.4	126.8	126.6	126.5	126.6

**Table S3. Molecular volume and topological polar surface area (tPSA) values.** For the bioisostere series these properties were calculated on the protonated ammonium species.		
**Compound**	**Molecular Volume ($Å^3$)**	**tPSA ($Å^2$)**
Benzene	84.04<br>80.3 (Lit) [1]	0.00
Furan	65.61	13.14
Thiophene	74.75	0.00
Selenophene	80.12	0.00
DPP	278.15	4.44
FuPP	259.71	17.58
TPP	268.86	4.44
3-TPP	268.86	4.44
SePP	274.22	4.44

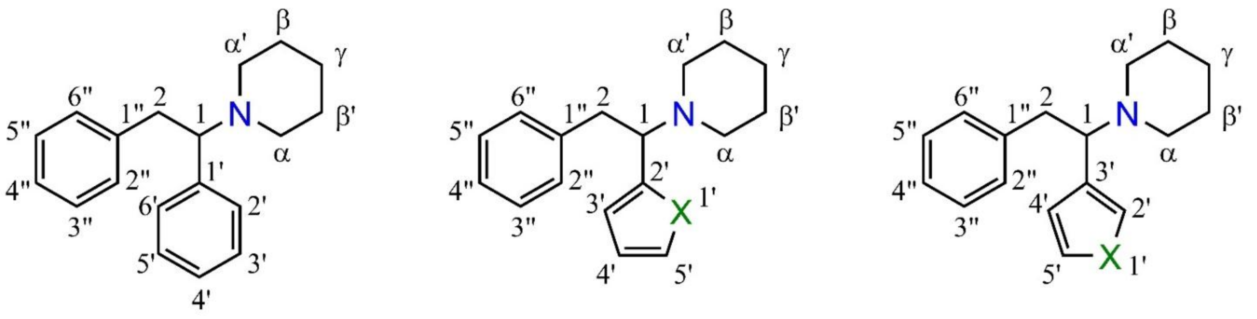

**Table S4. 1,2-Diarylethylamine Bioisostere Series Stockholder Charges for NH^+ Compounds.**					
**Atom Number**	**DPP**	**FuPP**	**TPP**	**3-TPP**	**SePP**
**C1**	0.05667	0.05613	0.05757	0.05848	0.05745
**C2**	-0.04056	-0.04041	-0.03913	-0.04039	-0.03935

**C1"**	-0.00002	-0.00179	-0.00093	-0.00060	-0.00009
**C2"**	-0.04694	-0.04654	-0.04042	-0.04723	-0.04655
**C3"**	-0.02829	-0.02755	-0.02534	-0.02795	-0.02721
**C4"**	-0.02376	-0.02301	-0.02243	-0.02328	-0.02249
**C5"**	-0.02585	-0.02609	-0.02657	-0.02554	-0.02501
**C6"**	-0.04075	-0.04194	-0.04625	-0.04083	-0.03924
**1'**	-0.00663	-0.08146	0.08673	0.11646	0.12582
**C2'**	-0.04565	0.04165	-0.03419	-0.05120	-0.05148
**C3'**	-0.02100	-0.04822	-0.05236	-0.02350	-0.05138
**C4'**	-0.01358	-0.05215	-0.04077	-0.06500	-0.04080
**C5'**	-0.01641	0.03547	-0.03628	-0.04111	-0.05107
**C6'**	-0.02975	-	-	-	-
**N**	0.08529	0.08467	0.08568	0.08538	0.08557
**NH**	0.14086	0.14307	0.14457	0.14432	0.14334
**Cα**	0.02387	0.02177	0.02093	0.02099	0.02371
**Cβ**	-0.03392	-0.03407	-0.03414	-0.03437	-0.03377
**Cγ**	-0.03587	-0.03570	-0.03583	-0.03590	-0.03587
**Cα'**	0.02193	0.02433	0.02397	0.02402	0.02140
**Cβ'**	-0.03433	-0.03357	-0.03371	-0.03386	-0.03420
**H1**	0.04619	0.05596	0.05005	0.04836	0.04858
**H2**	0.03676	0.03903	0.03714	0.03676	0.03767
**H2**	0.03849	0.03964	0.03942	0.03875	0.03863
**H2"**	0.03626	0.03733	0.03752	0.03617	0.03655
**H3"**	0.05153	0.05220	0.05238	0.05181	0.05222
**H4"**	0.05357	0.05400	0.05415	0.05398	0.05412
**H5"**	0.05204	0.05201	0.05234	0.05232	0.05235
**H6"**	0.03731	0.03682	0.03668	0.03692	0.03762
**H2'**	0.03734	-	-	0.06560	-
**H3'**	0.05690	0.06645	0.04800	-	0.04578
**H4'**	0.06005	0.07091	0.06469	0.04675	0.06279
**H5'**	0.05899	0.07709	0.07514	0.07326	0.07426

**H6'**	0.04788	-	-	-	-
**Hαax**	0.05493	0.05306	0.05352	0.05288	0.05499
**Hαeq**	0.05728	0.05020	0.04903	0.05000	0.05725
**Hβax**	0.03886	0.04015	0.03896	0.03899	0.03900
**Hβeq**	0.05827	0.05857	0.05895	0.05859	0.05844
**Hγax**	0.03811	0.03808	0.03842	0.03816	0.03822
**Hγeq**	0.05221	0.05258	0.05250	0.05239	0.05242
**Hα'ax**	0.05307	0.05481	0.05530	0.05495	0.05296
**Hα'eq**	0.05103	0.05825	0.05712	0.05708	0.04941
**Hβ'ax**	0.03923	0.03989	0.03910	0.03906	0.03929
**Hβ'eq**	0.05843	0.05855	0.05868	0.05851	0.05887

Table S5. 1,2-Diarylethylamine Bioisostere Series Stockholder Charges for Unionized Compounds.

Atom Number	DPP	FuPP	TPP	3-TPP	SePP
**C1**	0.03557	0.03309	0.03613	0.03683	0.03628
**C2**	-0.04332	-0.04579	-0.04534	-0.04437	-0.04268
**C1"**	0.01010	0.00897	0.01016	0.01016	0.01009
**C2"**	-0.04164	-0.04575	-0.04171	-0.04801	-0.04795
**C3"**	-0.03914	-0.04079	-0.04181	-0.04390	-0.04285
**C4"**	-0.04418	-0.04450	-0.04460	-0.04511	-0.04393
**C5"**	-0.04275	-0.04276	-0.04357	-0.04245	-0.03887
**C6"**	-0.04733	-0.04752	-0.04779	-0.04263	-0.03954
**1'**	0.01098	-0.07174	0.03632	0.03657	0.05357
**C2'**	-0.04090	0.05525	-0.02053	-0.07091	-0.02781
**C3'**	-0.04211	-0.07937	-0.06444	-0.01310	-0.05840
**C4'**	-0.04394	-0.07733	-0.05948	-0.05898	-0.05682
**C5'**	-0.03994	0.00740	-0.06766	-0.06536	-0.08500
**C6'**	-0.04182	-	-	-	-

**N**	-0.09290	-0.09794	-0.08878	-0.08929	-0.09175
**Cα**	-0.01095	-0.01175	-0.01168	-0.01064	-0.00812
**Cβ**	-0.04514	-0.04552	-0.04534	-0.04549	-0.04411
**Cγ**	-0.04469	-0.04392	-0.04354	-0.04358	-0.04450
**Cα'**	-0.00887	-0.01031	-0.00777	-0.00819	-0.01045
**Cβ'**	-0.04469	-0.04540	-0.04505	-0.04512	-0.04469
**H1**	0.01109	0.03259	0.03257	0.02818	0.01791
**H2**	0.02889	0.03031	0.02797	0.02705	0.02980
**H2**	0.02987	0.02847	0.03017	0.02994	0.03018
**H2''**	0.03493	0.03146	0.03467	0.03429	0.03407
**H3''**	0.03982	0.03904	0.03831	0.03825	0.03874
**H4''**	0.03862	0.03853	0.03845	0.03830	0.03889
**H5''**	0.03845	0.03865	0.03847	0.03801	0.04020
**H6''**	0.03411	0.03569	0.03466	0.03370	0.03527
**H2'**	0.03280	-	-	0.05092	-
**H3'**	0.03809	0.04756	0.04280	-	0.04184
**H4'**	0.03841	0.04963	0.04649	0.04362	0.04383
**H5'**	0.03951	0.05752	0.05425	0.05467	0.05116
**H6'**	0.03496	-	-	-	-
**Hαax**	0.00727	0.00750	0.00804	0.00872	0.00844
**Hαeq**	0.02555	0.02927	0.02557	0.02537	0.03070
**Hβax**	0.02438	0.02330	0.02291	0.02305	0.02547
**Hβeq**	0.02725	0.02642	0.02656	0.02592	0.02712
**Hγax**	0.02036	0.02089	0.02104	0.02104	0.02028
**Hγeq**	0.02590	0.02575	0.02611	0.02608	0.02661
**Hα'ax**	0.00720	0.00643	0.00714	0.00668	0.00820
**Hα'eq**	0.02959	0.02688	0.02860	0.02840	0.02545
**Hβ'ax**	0.02408	0.02351	0.02544	0.02528	0.02556
**Hβ'eq**	0.02650	0.02633	0.02630	0.02636	0.02791

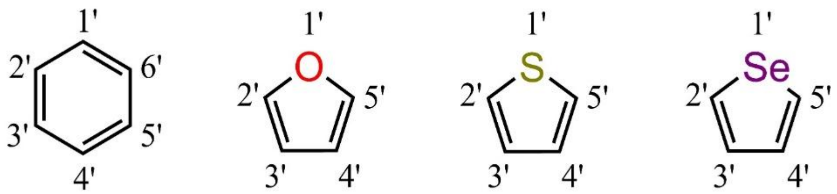

Table S6. Aromatic Ring Stockholder Charges.

Atom Number	Benzene	Furan	Thiophene	Selenophene
**1'**	-0.04009	-0.08003	0.04107	0.06519
**C2'**	-0.04008	0.00954	-0.06562	-0.07907
**C3'**	-0.04010	-0.07829	-0.05764	-0.05487
**C4'**	-0.04014	-0.07830	-0.05765	-0.05488
**C5'**	-0.04015	0.00954	-0.06562	-0.07913
**C6'**	-0.04013	-	-	-
**H1'**	0.04013	-	-	-
**H2'**	0.04013	0.05885	0.05517	0.05485
**H3'**	0.04013	0.04991	0.04758	0.04655
**H4'**	0.04013	0.04991	0.04758	0.04655
**H5'**	0.04013	0.05885	0.05517	0.05485
**H6'**	0.04013	-	-	-

Table S7. Predicted (Jaguar) pK_a for 1,2-Diarylethylamine Bioisosteres.

Compound	pK_a
Diphenidine	8.46
FuPP	8.17
TPP	8.10
3-TPP	8.60
SePP	9.14

**Table S8. Pauling Scale Electronegativity Values for C, O, S, and Se.**[2]	
**Compound**	Electronegativity (Pauling Scale)
C	2.55
O	3.44
S	2.58
Se	2.55

**Table S9. % Inhibition of [^{3}H]-Citalopram SERT Binding 10 µM Screening.**	
**Compound**	**% Inhibition of APP+ Uptake (Average, N =2)**
Citalopram	98.5
Diphenidine	-7.6
FuPP	-18.2
TPP	-0.50
3-TPP	-7.4
SePP	-11.4

**Table S10. Beam Test Scoring.**	
Score	Performance
1	Stable balance (no paw slips)
2	1-2 paw slips, mostly stable
3	3-4 paw slips, somewhat stable
4	5+ paw slips, unstable but did not fall from beam
5	Fell but held onto beam
6	Fell off beam

**Table S11. Concentration of SePP vs. SePP/IS Peak Area Ratio in Mouse Plasma.** Generated using the analyte/IS peak area ratios versus concentration (5-1,000 ng/mL) by LC-MS/MS.	
**Concentration (ng/mL)**	**Analyte/IS Ratio**
5	0.0017
10	0.0032
50	0.0155
100	0.0304
500	0.1681
1,000	0.3197

**Table S12. Plasma concentrations of SePP in individual mice at the 10-min time point as determined by LC-MS/MS.** Values represent determined concentrations (ng/mL) from calibration curve Fig. S8. Summary statistics (mean ± standard deviation) are shown across biological replicates (n = 4).		
**Sample and Summary Statistics**	**SePP Plasma Concentration (ng/mL)**	**SePP Plasma Concentration (nM)**
Mouse 1 - 10 min	379.1	1,190.9
Mouse 2 - 10 min	174.6	548.5
Mouse 3 - 10 min	358.7	1,126.9
Mouse 4 - 10 min	637.7	2,003.2
Mean ± SD	387.5 ± 164.9	1,217.4 ± 518.1

**Table S13. Plasma concentrations of SePP in individual mice at the 120 min time point as determined by LC-MS/MS.** Values represent determined concentrations (ng/mL) from calibration curve in Fig. S8. Summary statistics (mean ± standard deviation) are shown across biological replicates (n = 4).		
**Sample and Summary Statistics**	**Concentration (ng/mL)**	**SePP Plasma Concentration (nM)**
Mouse 1 - 120 min	30.3	95.3
Mouse 2 - 120 min	58.1	182.4
Mouse 3 - 120 min	21.4	67.3
Mouse 4 - 120 min	25.4	79.7
Mean ± SD	33.8 ± 14.4	106.2 ± 45.1

**Table S14. Calibration Curve for SePP in Mouse Brain Homogenate.** Generated using the analyte/IS peak area ratios versus concentration (5-5,000 ng/mL) by LC-MS/MS.	
**Concentration (ng/mL)**	**Analyte/IS Ratio**
5	0.0017
10	0.0031
50	0.0102
100	0.0187
500	0.1043
1,000	0.2108
5,000	1.0513

**Table S15. LC-MS/MS Compound Dependent Parameters for Analyte (SePP) and Internal Standard (Diphenidine).**		
**Parameter**	**SePP**	**Diphenidine**
Precursor ion *(m/z)*	320.09	266.19
Product ion (*m/z*)	234.9, 153.2	181.1, 103.1
Collision energy (V)	14, 39	17, 33
Tube lens off set (V)	43	4
Retention Time (min)	9.74	7.55

Supporting Information References

[1] Zhao, Y.H., Abraham, M.H. and Zissimos, A.M., 2003. Fast calculation of van der Waals volume as a sum of atomic and bond contributions and its application to drug compounds. *The Journal of organic chemistry*, *68*(19), pp.7368-7373.
[2] Tantardini, C. and Oganov, A.R., 2021. Thermochemical electronegativities of the elements. *Nature communications*, *12*(1), p.2087
